# Computational analysis of 140 years of US political speeches reveals more positive but increasingly polarized framing of immigration

**DOI:** 10.1073/pnas.2120510119

**Published:** 2022-07-29

**Authors:** Dallas Card, Serina Chang, Chris Becker, Julia Mendelsohn, Rob Voigt, Leah Boustan, Ran Abramitzky, Dan Jurafsky

**Affiliations:** ^a^Computer Science Department, Stanford University, Stanford, CA 94305;; ^b^School of Information, University of Michigan, Ann Arbor, MI 48109;; ^c^Department of Economics, Stanford University, Stanford, CA 94305;; ^d^Department of Linguistics, Northwestern University, Evanston, IL 60208;; ^e^Department of Computer Science, Northwestern University, Evanston, IL 60208;; ^f^Department of Economics, Princeton University, Princeton, NJ 08544;; ^g^National Bureau of Economic Research, Cambridge, MA 02138;; ^h^Department of Linguistics, Stanford University, Stanford, CA 94305

**Keywords:** immigration, metaphor, dehumanization, framing, Congress

## Abstract

We classify and analyze 200,000 US congressional speeches and 5,000 presidential communications related to immigration from 1880 to the present. Despite the salience of antiimmigration rhetoric today, we find that political speech about immigration is now much more positive on average than in the past, with the shift largely taking place between World War II and the passage of the Immigration and Nationality Act in 1965. However, since the late 1970s, political parties have become increasingly polarized in their expressed attitudes toward immigration, such that Republican speeches today are as negative as the average congressional speech was in the 1920s, an era of strict immigration quotas. Using an approach based on contextual embeddings of text, we find that modern Republicans are significantly more likely to use language that is suggestive of metaphors long associated with immigration, such as “animals” and “cargo,” and make greater use of frames like “crime” and “legality.” The tone of speeches also differs strongly based on which nationalities are mentioned, with a striking similarity between how Mexican immigrants are framed today and how Chinese immigrants were framed during the era of Chinese exclusion in the late 19th century. Overall, despite more favorable attitudes toward immigrants and the formal elimination of race-based restrictions, nationality is still a major factor in how immigrants are spoken of in Congress.

## Significance

In the first comprehensive quantitative analysis of the past 140 y of US congressional and presidential speech about immigration, we identify a dramatic rise in proimmigration attitudes beginning in the 1940s, followed by a steady decline among Republicans (relative to Democrats) over the past 50 y. We also reveal divergent usage of positive (e.g., families) and negative (e.g., crime) frames—over time, by party, and between frequently mentioned European and non-European groups. Finally, to capture more suggestive language, we introduce a method for measuring implicit dehumanizing metaphors long associated with immigration (animals, cargo, etc.) and show that such metaphorical language has been significantly more common in speeches by Republicans than Democrats in recent decades.

Immigration is one of the most important and divisive topics in American public life. From the rise of vocal antiimmigrant politicians in recent years, it is tempting to conclude that attitudes toward immigration are more negative—or at least more polarized—than ever before. However, resistance to newcomers has always been a central part of our public discourse about immigration. From anti-Chinese fearmongering in the 1880s to concerns about Southern and Eastern European immigrants in the 1920s to the antiimmigration rhetoric of the Trump administration (2017 to 2020), claims that certain types of immigrants can never truly join American society have been a perennial part of our discourse. For example, Senator Henry Cabot Lodge, an architect of antiimmigrant legislation, declared a century ago, “[Immigration] is bringing to the country people whom it is very difficult to assimilate” (1, p. 35) because immigrants are from “races most alien to the body of the American people” (1, p. 32).

We seek to move beyond individual anecdotes to ask, how have attitudes toward immigrants in the United States changed over the past century? How does recent political debate over immigration compare to the long sweep of US history? This question is a challenge because public opinion polls that asked about attitudes toward immigration only began in the 1960s and were then only asked about immigration sporadically until recent years. We instead turn to the Congressional Record and other sources of political speech, using quantitative text analysis methods to systematically investigate the language used in congressional and presidential speeches about immigration over the past 140 y.

Our paper considers the full corpus of more than 17 million congressional speeches from 1880 to the present, of which we identify ∼200,000 speeches relevant to the topic of immigration. We also incorporate presidential communications from the same time period, making this a comprehensive quantitative analysis of American political speech about immigration at the federal level, covering the entire time period from the Chinese Exclusion Act of 1882 to the present day.

Numerous studies have analyzed the political history of US immigration using qualitative approaches and historical archives (2–7); quantitative work on immigration has also used data such as migration and census records (8, 9). Rhetorical aspects of immigration debates have been studied qualitatively—especially the use of dehumanizing language and metaphors such as “vermin” and “cargo” (10–13)—but these authors have not rigorously quantified how common such language is over time. Last, other scholars have applied computational methods from natural language processing to study coverage of immigration in news media and Congress (14–18), but none have used these tools to investigate such a long time span or comprehensive corpus of speeches about US immigration with a consistent methodology.

Our analysis is based on a combination of methods. To identify relevant speeches, along with a corresponding tone (proimmigration, antiimmigration, or neutral), we make use of automated text classification based on extensive human annotations. Using a semiautomated process, we also curate and apply a set of lexicons for analyzing relevant frames (i.e., ways of characterizing immigrants and immigration). Finally, to quantify implicit dehumanizing metaphors in speeches, we develop an approach using neural contextual embedding models to measure if references to immigrants are suggestive of various metaphorical categories (*Materials and Methods*).

We find that political speeches about immigration today are far more likely to be positive than in the past, with the shift from negative to positive mostly taking place between World War II (WWII) and the passage of the 1965 Immigration and Nationality Act, and being net positive on average in nearly all sessions of Congress since the early 1950s. Extending this analysis to presidential communications, we find President Trump to be a stark exception, as the first president in modern American history to express sentiment toward immigration that is more negative than the average member of his own party. As with many political issues, the two parties have become increasingly polarized over time, and we find a linear increase in polarization on immigration, beginning in the late 1970s under President Carter. Today, Democrats are unprecedentedly positive about immigration, whereas Republicans are as negative as the average legislator was in the 1920s during the push for strict immigration quotas. This divergence is clearly part of a broader trend toward polarization on many issues (*Discussion*); for immigration specifically, our analysis reveals the beginnings of this, predating the rise in generic political polarization observed in Gentzkow et al. (19) by more than a decade.

Along with the polarization by party, nationality of immigrants continues to matter greatly, with speeches mentioning Mexican immigration being consistently more negative than the average (dramatically so in comparison to European groups). Moreover, there is a striking similarity between how Mexican immigrants are framed today and how Chinese immigrants were framed during the period of Chinese exclusion in the 19th century: more negative in tone; greater explicit emphasis on frames such as “crime,” “labor,” and “legality”; and significantly greater use of implicit dehumanizing metaphors, in comparison to European groups.

Thus, while far more members of Congress today express favorable attitudes toward immigration than in the past, there remains a strong and growing strain of antiimmigration speech, especially among Republicans, along with perennial references to threats, legality, and crime. Despite the elimination of country-specific immigration quotas in the 1960s, expressed opinions toward immigrants still vary greatly by country of origin, and enduring rhetorical strategies continue to be deployed against more marginalized groups.

## Results

### Tone of Immigration Speeches.

**Fig. 1.**
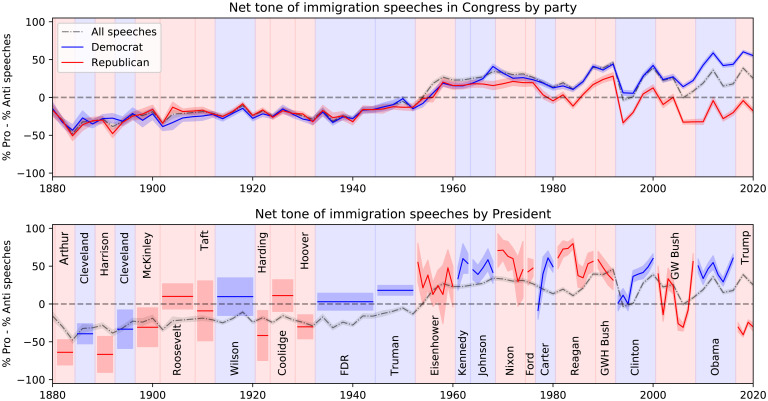
Evolution of attitudes toward immigration expressed in congressional speeches and presidential communications. Average tone is computed as the percentage of proimmigration speeches minus the percentage of antiimmigration speeches, where proimmigration means valuing immigrants and favoring less restricted immigration and vice versa. *Top* and *Bottom* show the overall tone using all congressional speeches about immigration (black dashed line, with bands showing plus or minus two SDs based on the estimated proportions and number of speeches). *Top* also shows separate plots for speeches by Democrats and Republicans in Congress. (Due to limitations of the data, about 15% of speeches do not have a named speaker or party affiliation.) *Bottom* shows the corresponding estimates for each president, showing the overall average for a president’s tenure when there are insufficient data to show annual variation. Note that most modern presidents have been more favorable toward immigration than the average member of Congress. By contrast, Donald Trump appears to be the most antiimmigration president in nearly a century. Similarly, congressional Republicans over the past decade have framed immigration approximately as negatively as the average member of Congress did a century earlier.

Starting with the complete record of 17 million congressional speeches from 1880 to 2020 (*Data*), we collected human annotations and trained machine learning classifiers to identify speeches relevant to immigration, along with an accompanying tone (proimmigration, antiimmigration, or neutral; *Classification*). Both panels of Fig. 1 show the average tone (percent proimmigration minus percent antiimmigration) expressed in congressional speeches over this time period (black line).* The trends for congressional speeches by Democrats and Republicans are also shown in Fig. 1, *Top*. A comparable time series for presidents is shown in Fig. 1, *Bottom*, by applying the same models to all presidential communications collected by the American Presidency Project (20). For alternative models, validity checks, and variation within parties, refer to *SI Appendix*.

We begin by documenting a number of findings about political speech related to immigration. First, average sentiment toward immigration in Congress and the executive branch is negative throughout the late 19th and early 20th centuries, from the passage of the Chinese Exclusion Act (1882) through the advent of strict immigration quotas in the 1920s. The pervasiveness of negative sentiment can help make sense of the political context that gave rise to a suite of increasingly restrictive immigration regulations. It is particularly noteworthy that we do not find a rise in negative speeches leading up to the Emergency Quota Act of 1921. Rather, we find that political sentiment in Congress was staunchly antiimmigration for more than 4 decades, which is consistent with the political history that has recounted the many congressional attempts to pass antiimmigration legislation, all of which were struck down by the president, in the years before the successful passage of quotas (21). Second, attitudes toward immigration became more positive around the start of WWII, rising steadily from 1940 until the end of the Johnson administration (1969). The average tone in Congress has essentially been proimmigration since the beginning of the Eisenhower administration (1953), consistent with efforts by postwar presidents to reframe the public understanding of immigration as positive for the country.

Third, beginning about a decade after the reopening of the border with the 1965 Immigration and Nationality Act, there has been a growing partisan divide, larger year-to-year variations, and an overall decline in sentiment toward immigration among Republicans. Democrats, by contrast, have grown more positive about immigration over time, especially under Presidents Obama and Trump, with the exception of a temporary bipartisan drop in proimmigration speeches in the early 1990s, coinciding with the end of the Cold War and the passage of the North American Free Trade Agreement (NAFTA). By contrast, Republican legislators are now approximately as overtly antiimmigration in their speeches as the average legislator was during the Age of Mass Migration from Europe and the 1920s quota periods.

The trends for presidential attitudes toward immigration should be treated more cautiously as there is less text available from presidents overall and because these estimates involve a slight domain shift (from congressional speeches, on which our models were trained, to more varied types of presidential communications). Nevertheless, we document a similar pattern, whereby early presidents were more antiimmigration than modern presidents. In recent years, presidents have been uniformly more proimmigration than the average member of Congress, including both Republicans like Ronald Reagan and Democrats like Jimmy Carter. In historical comparison, President Trump was a stark exception: by his utterances, he was the most antiimmigration president to sit in office over the past 140 y, relative to the average attitude of the time expressed in Congress.

**Fig. 2.**
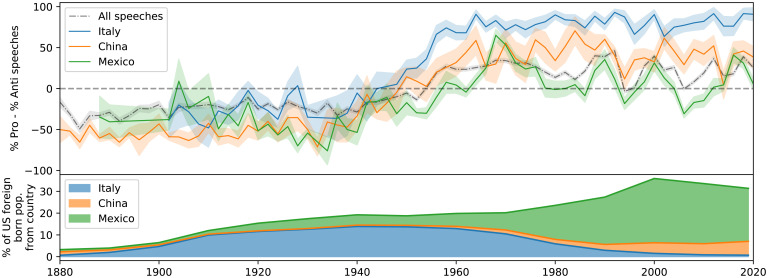
Average tone of immigration speeches when considering only those speeches that mention the country or nationality for each of the three most frequently mentioned nationalities (*Top*) and the percent of the US foreign-born population from each of these countries over time (*Bottom*). Despite the midcentury increase in proimmigration attitudes applying to all groups, a gap in tone by group persists to the present day, with Mexican immigrants being consistently framed more negatively than others and Italian immigrants being framed especially positively. These trends are mirrored in broader regional patterns for Europe, Asia, and Latin American and the Caribbean (*SI Appendix*).

Although the difference in tone between the parties today is larger than at any point in the past, tone also varies dramatically depending on which groups of immigrants are being discussed. Fig. 2 shows the average tone when considering only those speeches that mention each of the three most commonly mentioned nationalities in immigration speeches—Mexican, Chinese, and Italian (*Identifying Groups*).

Speeches mentioning Chinese immigrants were overwhelmingly negative during the period of Chinese exclusion (1882 to 1943), while the tone toward Italian immigrants was slightly more favorable (yet still negative) at the time. Attitudes toward all groups improved from 1940 to 1970, with mentions of Chinese and Mexican immigrants remaining relatively more negative overall. Mentions of Italian immigrants are overwhelmingly positive today, but since the late 1970s, the average gap in tone between speeches mentioning Mexican as opposed to Italian immigrants has remained approximately as large as the gap in tone that exists between Republicans and Democrats today.

Although few countries are mentioned as frequently as these three, this pattern is mirrored in broader regional trends: most European countries are referred to positively on average by the 1960s, Asian countries by the 1980s, with countries in the Caribbean (Haiti and Cuba) remaining negative on average until the 2000s. (See *SI Appendix* for trends for more individual countries, regional averages, and a regression analysis controlling for other factors.)

### Language, Framing, and Dehumanization.

To better understand the language that is suggestive of proimmigration or antiimmigration tone in the full corpus of immigration speeches, we train interpretable logistic regression models to approximate the predictions of our contextual embedding models and determine feature importance using Shapley values (22). Table 1 lists the most important words found using this approach for the early, transitional, and modern periods (see *Measuring Impact* for details).

**Table 1.** Most influential words for proimmigration and antiimmigration speeches, in three time periods, when approximating the predicted tone from our classification models with simpler logistic regression models

	Antiimmigration	Proimmigration
Early (1880 to 1934)	Chinese, undesirable, exclusion, violation, restriction, permit, dangerous, restrict, smuggled, cheap, excluded, deport, laborers	war, country, great, lands, gave, immigrants, entitled, property, relief, agriculture, served, give, rights, protection, glad, industrious
Transitional (1935 to 1972)	aliens, country, illegal, alien, deportation, united, criminals, subversive, fact, deported, America, system, deport, undesirable	life, humanitarian, families, migrant, opportunity, contributions, anniversary, citizens, hope, discriminatory, great, children, migrants
Modern (1973 to 2020)	illegally, control, foreign, policy, enforce, entry, people, national, terrorism, illegal, terrorists, stop, smuggling, INS, dangerous	community, young, immigrant, life, contributions, Hispanic, heritage, dream, victims, Irish, proud, important, Italian, work, treatment, urge

Among early antiimmigration terms, we find words representing threats (“dangerous” and “cheap”), control (“permit” and “violation”), and the targets of early antiimmigration legislation (“undesirable” and “Chinese”). By midcentury and beyond, different threats appear (first “subversive” and eventually “terrorism”), along with themes of legality (“aliens” and “illegal”) and crime (“criminals” and “smuggling”), both of which continue into the present.

Among proimmigration terms, we see an early focus on “desirable” characteristics (“industrious”), land (“property” and “agriculture”), and service (“gave” and “served”). The post-WWII era saw the rise of “humanitarian” concerns (“discriminatory” and “migrants”) and an emphasis on community and belonging (“citizens,” “families,” and “children”). These too continue into the present (“victims” and “community”), along with a celebration of once-vilified communities (“Irish,” “Italian,” and “heritage”).

Interestingly, despite the relatively negative tone associated with Mexican immigrants in the modern period (compared to other groups), we do find strong positive associations with the terms “Hispanic” and “Latino,” which refer to much broader communities. Part of the reason is likely that these terms are much more commonly used by Democrats than Republicans (dramatically so in the case of “Latino”) and hence are indicators of Democratic speeches, which are more likely to be proimmigration. Importantly, however, references to “Mexico” and “Mexican” are still more frequent in our corpus and indeed are mentioned with very similar frequency by Democrat and Republicans in the context of immigration (3.7 vs. 3.5 × 10^−4^, respectively), meaning that the observed tone difference by group is not simply a matter of Mexico primarily being mentioned by Republicans.

In order to understand the rhetorical divergence between parties in terms of how they characterize immigration at a more general level, we focus on several important aspects (i.e., frames) of the debate on immigration. As a direct and transparent way of measuring the prevalence of these frames, we build and share a series of lexicons for this issue. Drawing upon prior work on the framing of immigration in the media (23, 24), we develop 14 of these lexicons using a combination of automated term selection and manual curation (*Curating Frames*).

**Fig. 3.**
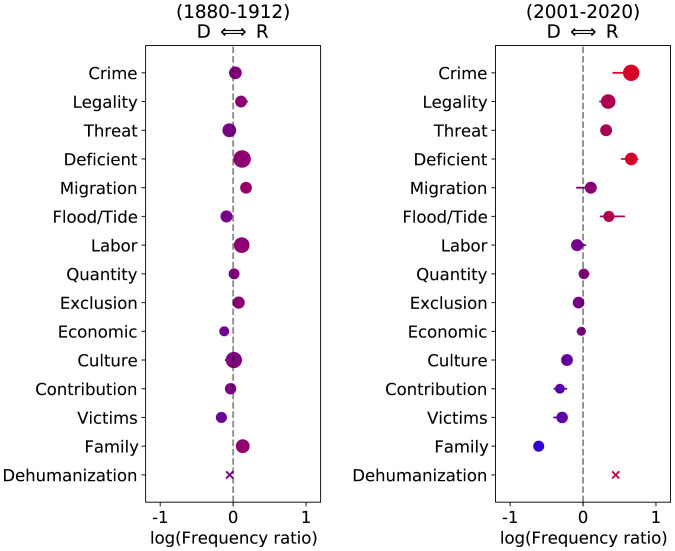
Relative usage frequency for each of 14 frames by Republicans compared to Democrats, both for the late 19th/early 20th century (*Left*) and the past 2 decades (*Right*). Farther to the left on each plot represents more frequent usage by Democrats and vice versa (plotted as log frequency ratio). Circle size represents the overall prominence of the frame in speeches about immigration, relative to all speeches. To ensure the robustness of these findings, we leave out each word in turn from each frame and show the full range of possible values obtained using horizontal lines (not visible when the full range is contained within the circle). “Dehumanization” is an aggregation of metaphorical categories (see *Measuring Dehumanization*). Compared to the absence of polarization a century ago, certain frames today are disproportionately used by Republicans (“crime,” “legality,” “threats,” “deficiency,” and “flood/tide”) and Democrats (“family,” “victims,” “contributions,” and “culture”). Republicans also show significantly higher use of implicit dehumanizing metaphors like “animals” and “cargo.”.

Fig. 3 shows the relative usage of each of the 14 frames by party, for both the past 2 decades (Fig. 3, *Right*) and a century earlier (Fig. 3, *Left*). There is almost no difference in the frames used by the two parties in the earlier time period. By contrast, speakers make strongly divergent use of different frames today, with Republicans more likely to explicitly frame immigration in terms of “crime,” “legality,” “threats,” “deficiency,” and the notion of a “flood/tide” of immigrants. Many of the terms driving this association will be familiar from commonly heard antiimmigration comments, including “flood,” “pouring,” “illegal,” “smuggling,” “stealing,” and “cheap.”

Democrats, by contrast, are more likely to emphasize the positive frames of “family,” “victims,” “contributions,” and “culture,” which is reflected in terms in our lexicons such as “hardworking,” “worthy,” “parents,” “children,” “integrating,” “diverse,” and “contributing.” Although some terms carry more weight than others in these measures, many terms contribute to each frame, and these patterns are robust to the exclusion of any individual term (shown by lines in Fig. 3), as well as to automated lexicon expansion (plots in *SI Appendix*).

Fig. 3 also shows the prominence of each frame, with circle size indicating the frequency of usage of each frame within immigration speeches relative to all speeches. Whereas the most salient aspects of immigration in the earlier time period were “deficiency,” “culture,” and “labor,” today the most salient frames are “crime” and “legality.”^†^ Interestingly, although terms related to “economics” are not uncommon in speeches about immigration (e.g., “fund,” “tax,” and “budget”), they are even more common in nonimmigration speeches, making it the least salient frame in both time periods and also one that is used to equal degree by both parties.

In addition to these frames that appear explicitly in the text, we also measure more implicit dehumanizing metaphors. Among the metaphors that past work has called attention to in coverage of immigration, only the metaphor of a “flood” or “tide” of immigrants emerged from our semiautomated frame construction process. To be able to study more subtle dehumanizing language, we develop a way of measuring metaphors based on how probable such terms are as substitutes, according to contextual embedding models (*Identifying Mentions* and *Measuring Dehumanization*). Using this method, we measure the extent to which mentions of immigrants in speeches “sound like” a mention of several metaphorical categories that have been previously discussed in the literature on immigration: “animals,” “cargo,” “disease,” “flood/tide,” “machines,” and “vermin” (10–13, 25).^‡^

For example, the following sentence is detected as strongly cueing the “cargo” metaphor: “I voted last week for an antidumping bill to prevent the dumping of manufactured products into this country, and I will vote for any bill to prevent the dumping of undesirable […] into this country.” Similarly, the following sentence strongly cues the “animal” metaphor: “the herding of these […] into stockades is pictured.”

As shown at the bottom of Fig. 3, Republicans over the past 2 decades show significantly greater usage of implicit dehumanizing metaphors than Democrats.^§^ This difference also holds for most metaphorical categories considered individually (higher usage by Republicans than Democrats in recent decades). Additional examples and validity checks are included in *SI Appendix*.

### Differences by Country of Origin.

As shown above, the differences in tone between mentions of immigrants of different nationalities can be as large as the modern differences between parties. To better understand these differences, we focus on the most frequently mentioned immigrant groups in the past (from China) and today (from Mexico). In particular, we focus on Chinese immigrants during the period of Chinese exclusion up to the start of WWI and Mexican immigrants over the past 2 decades, comparing each to mentions of immigrants from European countries during the same time period.

**Fig. 4.**
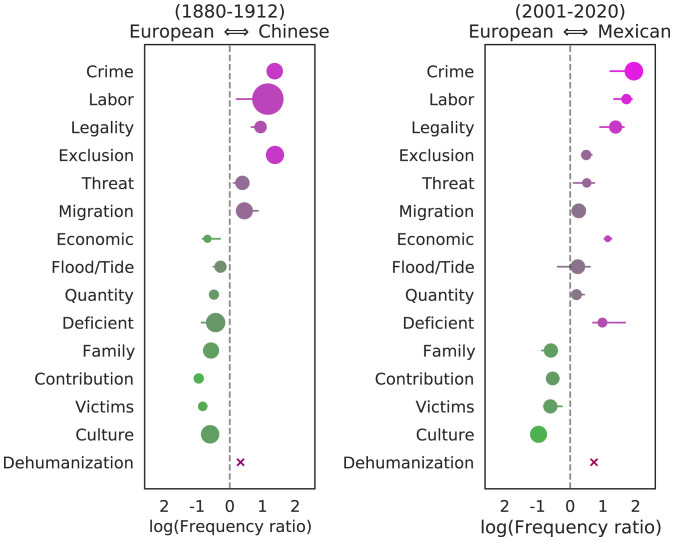
Relative usage frequency for each of 14 frames in speeches mentioning Chinese vs. European immigrants in the late 19th/early 20th century (*Left*) and those mentioning Mexican vs. European immigrants in the 21st century (*Right*). Farther to the left on each plot represents greater usage in speeches mentioning European groups. Circle size represents the overall frequency of the frame in the relevant speeches relative to all speeches. Horizontal lines show the minimum and maximum values of the log ratio obtained when leaving out each term in the corresponding lexicon in turn. “Dehumanization” is an aggregation of the six metaphorical categories. There is a strong correlation between how Mexican immigrants are framed today and how Chinese immigrants were framed a century earlier, relative to European immigrants of the corresponding time period, in terms of both the explicit frames emphasized and a significantly higher usage of dehumanizing metaphors for mentions of the non-European groups.

Fig. 4 shows the usage of each of the 14 frames for both groups. There is a strong similarity between how Mexican immigrants are being framed by politicians today and how Chinese immigrants were framed a century earlier, relative to European immigrants of the corresponding time periods. In particular, the frames of “crime,” “labor,” and “legality” are deployed vastly more in sentences mentioning the non-European group. Similarly, the four most positive frames (those that are prominently emphasized by Democrats today: “culture,” “victims,” “contributions,” and “family”) are all used far more in sentences mentioning European immigrants than the non-European groups. In addition, implicit dehumanizing language is slightly but significantly more common for mentions of the non-European group in both cases.^¶^

## Discussion

Much historical attention has been devoted to the period leading up to the immigration quotas in the 1920s and the nativist opposition to so-called “new” immigrants from Southern and Eastern Europe (2). Consistent with work which has focused specifically on earlier Chinese immigration (26, 27), we find that congressional antagonism to immigration started much earlier than the quota period. China occupied an especially prominent place in 19th century congressional debates on the issue, being mentioned in more than 20% of the immigration related speeches in Congress over the years 1880 to 1900. As discussion of the issue increased and broadened in the 20th century, with repeated attempts to pass legislation to restrict immigration (including a literacy test and country-specific quotas), our results show that attitudes toward immigration in Congress remained consistently negative from 1880 to 1940.

The negative tone toward Chinese immigrants is entirely consistent with the many pieces of anti-Chinese legislation introduced into Congress during this time. Despite representing less than 1% of the foreign-born population in 1900, the Chinese were subject to numerous restrictions, including the 1875 Page Act, the 1882 Chinese Exclusion Act, and the 1888 Scott Act. It is notable that mentions of Chinese immigration remained frequent and negative until just before the Chinese Exclusion Act was repealed, in 1943. Moreover, by comparing mentions of Chinese immigrants to those of Europeans, we find that the language used to describe the former showed significantly greater use of implicit dehumanizing language and greater explicit emphasis on the threatening aspects of immigration (“crime” and “threats”), as well as related aspects like “labor” and “legality.”

This combination of frames underscores the dual nature of how Chinese immigrants were perceived—both as a threatening, immoral outsider and as a potential source of cheap labor (6)—which is a pattern we see reproduced in the discussion of Mexican immigration today. By contrast, the framing of Europeans was relatively more sympathetic (“victims,” “contributions,” etc.), although still negative until the middle of the 20th century.

America saw a gradual loosening of immigration laws in the 1940s—issuance of a small number of special visas during WWII, the repeal of the Chinese Immigration Act in 1943, and the Displaced Persons Act of 1948—laying the groundwork for efforts by President Truman, and later Presidents Kennedy and Johnson, to redefine America as a “nation of immigrants” (7). We find this trend mirrored by congressional tone toward immigration, which began improving in the 1940s, eventually becoming net positive on average in the 1950s, and building toward a bipartisan peak in the late 1960s.

The causes of these changes in policy and expressed attitudes are complex—past work has pointed to the practical problem of labor shortages in the midcentury economy and humanitarian reactions to the Nazi genocide (5, 6). Our work primarily shows evidence for the latter explanation, with increasing prominence of humanitarian concerns signaling positive attitudes from the 1940s onward and a significantly increasing association with the “victims” frame during this time, as well as a significant decrease in the prominence of “deficiency” and “threats” (see temporal analysis of frames in *SI Appendix*).

It is particularly striking that for nearly 30 y after the border reopened in 1965, the positive sentiment toward immigration did not fully erode, even as immigration from developing countries like Mexico, China, and India increased greatly. Instead, an enduring partisan divide on immigration emerged in the late 1970s, although the Republican party in Congress remained neutral or positive toward immigration, on average, until the election of Bill Clinton and the creation of NAFTA in the early 1990s. The timing of this polarization predates that found in Gentzkow et al. (19), who found overall polarization (based on how identifiable parties are based on their language) increased sharply after 1990, and for immigration after 2000, using unsupervised methods.

A broad literature in political science has documented rising partisan polarization in the United States. Much of this literature focuses on differentiating sources of polarization and unpacking mechanisms underlying its emergence. Examples include differentiating between affective vs. ideological polarization (28, 29), comparing local vs. national polarization (30), understanding the relationship between elite vs. public polarization (31, 32), and understanding the role of exposure to other views (33, 34) or economic inequality (35) in driving polarization. Our results are consistent with these broader patterns with a focus on one especially divisive issue. Importantly, our contribution focuses on comparing the tone of immigration speeches, which allows us not only to say that attitudes have polarized but also to compare the overall positivity/negativity of attitudes toward immigrants in the past vs. the present. By focusing on language we are able to go beyond a simple characterization of positive vs. negative sentiment and unpack the framings used in immigration debates between the past and present.

Understanding the causes of this polarization, and whether attitudes on immigration are being driven in a top-down or bottom-up manner, is largely beyond the scope of this paper. However, additional analyses in *SI Appendix* reveal that legislators’ tone on immigration is (weakly) correlated with public opinion on the issue at the state level, after correcting for year fixed effects. On the other hand, we do not find any evidence of systematic differences in tone among House members in election vs. nonelection years (*SI Appendix*), although this question is worthy of further investigation.

When considering the groups being mentioned, we find stark differences in framing between European and non-European groups, both for Chinese immigrants in the late 19th and early 20th century and for Mexican immigrants today. In both cases, more implicitly dehumanizing metaphors are used to describe the non-European group. There is also a striking similarity in the use of explicit frames, with a greater emphasis on “crime,” “labor,” and “legality” for the non-Europeans and less on “family,” “contributions,” “victims,” and “culture.” Moreover, although immigrants of nearly all of the most frequently mentioned nationalities are now mentioned in overwhelmingly positive terms on average, this emerged more slowly for those from Asia, the Caribbean, and Latin America. Mexico remains the strongest outlier, with a persistent gap in tone between mentions of European (e.g., Italian) and Mexican immigrants, equivalent to the modern gap between Democrats and Republicans.

Today, people from Mexico represent the largest proportion of entrants to the United States, and the country or nationality was mentioned in more than 10% of immigration speeches over the past 2 decades. As scholars have documented, many modern immigration laws and the rhetoric of “illegal” immigrants were crafted specifically to target immigration from Mexico, including seasonal workers and other temporary workers without full citizenship (36, 37). As such, it is in line with our expectations that there would be associations with “crime,” “legality,” and “labor,” but the extent of the differences by nationality are striking.

Much like China, people from Mexico were the target of early discrimination and institutionalized inequality in the United States, including the delayed statehood of New Mexico due to its large Mexican and Indian population (6). Mexico was exempt from the quota system, but despite the reliance of the United States on labor from Mexico, widespread opposition to Mexican immigrants began around the same time, and large numbers were subject to deportation or strongly encouraged to repatriate in the 1920s and 1930s (38, 39). Although we find the tone of speeches mentioning Mexican immigrants increased at a similar rate as other nationalities during the period after WWII, these gains were largely eroded in the early 1970s, leading to the persistent nationality-based gap that exists today.

Complementary insights can be derived from public opinion polls, which also reflect the increase in proimmigrant sentiment that we observe in our series from 1965 to the present. For the past 50 y, Gallup has asked Americans, “On the whole, do you think immigration is a good thing or a bad thing for this country today?” In 2019, a staggering 77% of Americans answered that immigration was a good thing. This was up from the low point of 52% in 2002, the year after the September 11 attacks. Respondents are also asked, “In your view, should immigration be kept at its present level, increased or decreased?” In the mid-1990s, a full 65% of respondents said that immigration should be decreased; in 2020, that number fell to just 28%—the lowest share to ever answer this question in the affirmative (40).

However, our analysis of congressional and presidential speeches—which provide a consistent measurement over a much longer period of time than is available from opinion polls—shows the picture is more complicated. Not only has party polarization been growing steadily over time, attitudes among Republican legislators are as negative toward immigration as members of Congress were during the push for restrictive quotas. Moreover, although Chinese immigrants are spoken of in largely favorable terms today, they are still discussed more negatively than immigrants from Europe.

In addition, recent years have seen a resurgence in anti-Asian sentiment and hate crimes (41–43), anti-Chinese rhetoric related to COVID-19, and greater restrictions on movement (including both pandemic-related restrictions and country-specific travel bans). Despite the proimmigration attitudes among the general population and the formal elimination of restrictions based on country and race, tone differences in Congress based on nationality are as strong as ever, with a difference between the parties that continues to grow. The United States truly is a nation of immigrants, with a complicated history that is both celebrated and condemned, but attitudes in Congress reveal that nationality and geography remain important factors in who is considered, by the US government, to be a desirable as opposed to undesirable part of the population.

## Materials and Methods

### Data.

For the 43rd through the 111th sessions of Congress, we used a digitized copy of the Congressional Record from Gentzkow et al. (44). For the 112th through the 116th Congress, we used the “congressional-record” tool provided by the @unitedstates project to download and extract the text of the Congressional Record from public HTML files (45). Both of these sources provide a speaker, party, state, and date for most speeches. Procedural speeches were automatically identified and excluded, as described in *SI Appendix*. For presidential communications, we downloaded all available presidential documents from The American Presidency Project (20). For immigration statistics, we combined data from table Ad354-443 of the Historical Statistics of the United States Millennial Edition Online (46) and census data compiled by the Migration Policy Institute (47). (Additional details are given in *SI Appendix*.)

### Classification.

We hired research assistants at Princeton University to label a random sample of speeches from the Congressional Record as 1) being about immigration or not and 2) for those relevant to immigration, being proimmigration, antiimmigration, or neutral. Because of the relative rarity of speeches about immigration (about 1% of all speeches), an extensive set of keywords was used to select possible segments for annotation, although all speeches were eventually classified as relevant or not, as described below.

A team of five annotators provided judgements on a total of 7,626 segments (of which 3,643 were judged relevant), with at least two annotations for most segments. Although not all segments can easily be classified as proimmigration, neutral, or antiimmigration, annotators showed reasonable levels of agreement on both relevance and tone (average Krippendorff’s alpha was 0.76 for relevance and 0.48 for tone; details in *SI Appendix*) and comparable agreement rates across time, indicating that they did not have substantially more difficulty with data from the earlier time period, despite having less familiarity with the language and politics of that time. These judgements were aggregated using a Bayesian item response model to obtain a probability distribution over labels for each segment, while accounting for individual annotator biases (48) (details in *SI Appendix*).

The annotated segments with inferred labels were then used to train relevance and tone classifiers, building on a recent neural language model, RoBERTa (49). As is common practice, we first fine-tuned the pretrained roberta-base model to congressional speeches in a self-supervised fashion (to adapt it to the domain) and then further fine-tuned it to be a classifier using our annotated examples. Overall, the classifiers achieve ∼90% accuracy on relevance and 65% accuracy on tone, although the vast majority of tone errors are between neutral and one of the extremes (proimmigration or antiimmigration). Moreover, models trained separately on the earlier and later parts of the data produce similar aggregate results in the intervening years (plots in *SI Appendix*).

Finally, these classifiers were used to identify relevant segments of congressional speeches, along with a predicted tone, aggregating predictions on segments into predictions on speeches. The raw annotations in aggregate show very similar patterns to the predicted labels on the full set of speeches, further demonstrating the validity of our findings (plots in *SI Appendix*). The same classifiers were also applied to presidential communications, treating paragraphs as individual segments for classification. To encourage replication and further research, we make available both the raw annotations and relevant speeches with predicted labels as part of the accompanying online materials.

### Identifying Groups.

To identify the most prominent immigrant nationalities, we began with historical data on the countries of origin of the foreign-born US population over time. Based on decadal counts, we identify 45 countries that accounted for at least 1% of the foreign-born population in at least 1 decade. For each, we then manually identified the country name and variations (e.g., Ireland), the associated nationality (e.g., Irish), other common ways of referring to these groups (e.g., Irishman and Irishmen), and modern hyphenated forms (e.g., Irish-American and Irish-Americans). The average tones of speeches that include any of these references are shown for the 3 and 14 most frequently mentioned nationalities in Fig. 2 and in *SI Appendix*, respectively.

### Measuring Impact.

To identify the terms that are most important for proimmigration or antiimmigration tone in our corpus (Table 1), we trained L1 regularized logistic regression models to fit the predicted tone labels on all congressional segments classified as relevant (based on the RoBERTa classifiers), thus approximating the influence of individual words. The vocabulary was restricted to words that occur at least 20 times, excluding numbers, punctuation, and stop words from Mallet, and counts were binarized.^ǁ^ Shapley values were computed for each term using the shap Python package (22). For both proimmigration and antiimmigration, Table 1 shows the top terms with the highest Shapley scores in each of three time periods in Congress.

### Curating Frames.

To identify and measure the prevalence of certain key aspects of immigration speeches, we curated lexicons for 14 immigration frames (thematic groups of words). To do so, we began by identifying terms (along with part of speech tags) that occurred significantly more frequently in reference to mentions of immigrants compared to mentions of generic people (“man,” “woman,” etc.). Based on a combination of initial exploration, comments from annotators, and prior literature on mass media coverage of immigration (23, 24, 50), we identified 14 relevant categories. Finally, the authors of this paper made independent judgments about which frame(s) each selected term should be part of (along with “other”), and these individual judgements were aggregated using majority votes. The full lists of terms, along with additional details, are given *SI Appendix*.

### Identifying Mentions.

To identify references to foreign-born people in our corpus, we collect direct mentions (e.g., “immigrants” and “displaced persons”), as well as group terms (e.g., “Germans”), and more generic person references with an associated nationality (e.g., “German laborers”), in speeches that we have classified as being about immigration. The sentences in which these mentions appear were then used to measure dehumanizing metaphorical language for each group (see below). For the comparison of sentences mentioning European vs. non-European immigrants (Fig. 4), we also include slang and derogatory terms to identify groups (e.g., “coolies” as a reference to early Chinese immigrants). For complete lists of mention terms and phrases, please refer to the online replication code.

### Measuring Dehumanization.

Past work has attempted to measure dehumanization using static word vectors (51) but has done so in a way that is insensitive to context. To improve upon this, we introduce a method that is based purely on context, measuring how much mentions of immigrants sound like particular types of dehumanizing metaphors, based on the sentences in which they occur.

To do so, we make use of a masked language model called BERT (Bidirectional Encoder Representations from Transformers), which was pretrained to predict the identity of missing words given the surrounding context (52). Rather than fine-tuning the model to act as a classifier (as we did when training classifiers for relevance and tone), we make use of the fact that the model can assign a probability to each word in its vocabulary given the surrounding context.

In order to repurpose this model to detect implicit metaphorical language, we began with several metaphors that have been previously described in the literature in immigration and dehumanization, such as “animals” and “cargo” (10–13, 25). For each one, we started with a list of terms that are representative of that category (“animal,” “animals,” types of animals, etc.) and used static vectors to find many similar terms. We then kept all the words from these lists that are part of BERT’s vocabulary, attempting to find all the words in the vocabulary that are representative of each metaphor.**

For each sentence that mentions an immigrant or immigrant group (*Identifying Mentions*), we remove the mention (e.g., “foreigners”) from the sentence, replacing it with a special “[MASK]” token—indicating a gap to be filled—producing a sentence with a masked word (e.g., “the tendency of [MASK] to flock together”). We then process the masked sentences through the model and compute how likely it is—according to the model—that the gap would be filled by each term in each of our metaphorical categories. We then add up the probabilities for each word in each category to get an overall score for each category for that sentence. The overall “dehumanization” scores in Figs. 3 and 4 sum the probabilities for all words in all categories and show the (log) ratio of the mean probability for one set of mentions (e.g., by Republicans) to the mean probability for the other (e.g., by Democrats). The significance of the difference of means is computed using a permutation test given the full set of mentions. Figures in *SI Appendix* show trends over time for each individual category, as well as examples.

To validate this method, we collected human judgements on a sample of masked contexts. For each such context, three of the authors of this paper independently rated whether an animal term would be a plausible replacement for the mask token, given the surrounding context. The annotations showed reasonably strong agreement (Krippendorff’s alpha = 0.59) and correlated strongly with the log probabilities assigned by the model (*r* = 0.73), showing that this method is a reliable way of measuring metaphorical language at scale (details in *SI Appendix*).

## Supplementary Material

Supplementary File

## Data Availability

As described in *Data* in *Materials and Methods*, congressional speeches were downloaded from two sources, which are available at https://data.stanford.edu/congress_text (44) and https://github.com/unitedstates/congressional-record/ (45). Presidential speeches were downloaded from The American Presidency Project and are available at https://www.presidency.ucsb.edu/ (20). As also described in *Data*, two sources of historical immigration statistics were used and are available from https://hsus.cambridge.org/ (46) and https://www.migrationpolicy.org/ (47). As described in *Details of Annotations for Relevance and Tone* in the *SI Appendix*, additional annotations from ref. 53 were used in this work, and have been included in our online data repository. As also described in *SI Appendix*, additional analyses not included in the main paper made use of DW-NOMINATE scores from https://voteview.com (54), Gallup polling data from https://ropercenter.cornell.edu (55), and demographic data from https://www.ipums.org (56), and all of these data have been included in our online data repository. All additional data and replication code for this project, including annotations and model predictions, have been deposited in our publicly accessible online repository, and are available for download at https://github.com/dallascard/us-immigration-speeches/ (57).

## Footnotes

^*^Many speakers hold nuanced positions on immigration, which resist easy categorization; nevertheless, our classifiers identify the extent to which speeches sound like proimmigration or antiimmigration speeches, as identified by our annotators. Additional plots in *SI Appendix* show the frequency of immigration speeches over time, relative frequency of neutral speeches, estimated tone for individual members of Congress, and validity checks on these findings using models which incorporate differences across regions and chambers.

^†^The modern prominence of the “crime” and “legality” frames is partly due to frequent mentions of “legal” and “illegal” immigrants but is also influenced by other legal terms (e.g., “laws” and “visas”) and crime terms (e.g., “criminals” and “terrorism”). Complete lists of terms can be found in *SI Appendix*.

^‡^The Congressional Record does contain a few examples of explicit expression of these ideas—for example, Rep. Hemenway (R, IN) in 1902 referred to Chinese immigrants “living in holes and swarming like vermin”—but these are rare. The sole exception is “water” terms (“flood,” “tide,” “flow,” etc.), which are very common in reference to immigration.

^§^Terms corresponding to the combined dehumanizing categories are about 1.6 times more probable as replacements in speeches by Republicans compared to Democrats (*P* < 0.001 by a permutation test).

^¶^Terms corresponding to the combined metaphorical categories are about 1.4 and 1.9 times more probable for mentions of Chinese and Mexican immigrants compared to Europeans, respectively (*P* < 0.001 by a permutation test in each case). Running a comparable analysis comparing recent European immigrants to those from Latin America more broadly shows a similar pattern of frames but no significant difference between these groups in our dehumanization metric (*SI Appendix*).

^ǁ^See https://github.com/mimno/Mallet/blob/master/stoplists/en.txt.

^**^BERT uses a vocabulary of approximately 30,000 words and “word pieces”—sequences of letters which can be concatenated to form any word in English. We only keep terms that are included as whole words in the BERT vocabulary. The complete list of terms is included in *SI Appendix*.

## References

[1] H. C. Lodge, The restriction of immigration. North Am. Rev. 152, 27–36 (1891).

[2] J. Higham, Strangers in the Land: Patterns of American Nativism, 1860-1925 (Rutgers University Press, New Brunswick, NJ, 1955).

[3] D. King, Making Americans: Immigration, Race, and the Origins of the Diverse Democracy (Harvard University Press, Cambridge, MA, 2002).

[4] D. J. Tichenor, Dividing Lines: The Politics of Immigration Control in America (Princeton University Press, Princeton, NJ, 2002).

[5] R. Daniels, Guarding the Golden Door: American Immigration Policy and Immigrants Since 1882 (Hill and Wang, New York, 2004).

[6] K. M. Parker, Making Foreigners: Immigration and Citizenship Law in America, 1600–2000, New Histories of American Law. (Cambridge University Press, 2015).

[7] J. L. Yang, One Mighty and Irresistible Tide: The Epic Struggle Over American Immigration, 1924-1965 (W. W. Norton, 2020).

[8] R. Abramitzky, L. Boustan, K. Eriksson, Do immigrants assimilate more slowly today than in the past? Am. Econ. Rev. Insights 2, 125–141 (2020). 32968736 10.1257/aeri.20190079 PMC7508458

[9] R. Abramitzky, L. Boustan, E. Jacome, S. Perez, Intergenerational mobility of immigrants in the united states over two centuries. Am. Econ. Rev. 111, 580–608 (2021).

[10] O. S. Ana, Brown Tide Rising (University of Texas Press, 2002).

[11] G. V. O’Brien, Indigestible food, conquering hordes, and waste materials: Metaphors of immigrants and the early immigration restriction debate in the United States. Metaphor Symb. 18, 33–47 (2003).

[12] N. Haslam, Dehumanization: An integrative review. Pers. Soc. Psychol. Rev. 10, 252–264 (2006). 16859440 10.1207/s15327957pspr1003_4

[13] K. Cunningham-Parmeter, Alien language: Immigration metaphors and the jurisprudence of otherness. Fordham Law Rev. 79, 1545 (2011).

[14] V. A. Nguyen, J. Boyd-Graber, P. Resnik, K. Miler, “Tea party in the House: A hierarchical ideal point topic model and its application to Republican legislators in the 112th Congress” in Proceedings of the 53rd Annual Meeting of the Association for Computational Linguistics and the 7th International Joint Conference on Natural Language Processing (Volume 1: Long Papers). C. Zong, M. Strube, Eds. (Association for Computational Linguistics, 2015), pp. 1438–1448.

[15] D. Card, J. Gross, A. Boydstun, N. A. Smith, “Analyzing framing through the casts of characters in the news” in Proceedings of the 2016 Conference on Empirical Methods in Natural Language Processing. J. Su, K. Duh, X. Carreras, Eds. (Association for Computational Linguistics, 2016), pp. 1410–1420.

[16] A. Webson, Z. Chen, C. Eickhoff, E. Pavlick, “Are “undocumented workers” the same as “illegal aliens”? Disentangling denotation and connotation in vector spaces” in Proceedings of the 2020 Conference on Empirical Methods in Natural Language Processing (EMNLP). B. Webber, T. Cohn, Y. He, Y. Liu, Eds. (Association for Computational Linguistics, 2020), pp. 4090–4105.

[17] S. Roy, D. Goldwasser, “Weakly supervised learning of nuanced frames for analyzing polarization in news media” in Proceedings of the 2020 Conference on Empirical Methods in Natural Language Processing (EMNLP). B. Webber, T. Cohn, Y. He, Y. Liu, Eds. (Association for Computational Linguistics, 2020), pp. 7698–7716.

[18] J. Mendelsohn, C. Budak, D. Jurgens, “Modeling framing in immigration discourse on social media” in Proceedings of the 2021 Conference of the North American Chapter of the Association for Computational Linguistics: Human Language Technologies, K. Toutanova ., Eds. (Association for Computational Linguistics, 2021), pp. 2219–2263.

[19] M. Gentzkow, J. M. Shapiro, M. Taddy, Measuring Group Differences in High Dimensional Choices: Method and Application to Congressional Speech. Econometrica 87, pp. 1307–1340 (2019).

[20] J. Woolley, G. Peters, *The American Presidency Project* (2021). https://www.presidency.ucsb.edu/. Accessed 2 May 2021.

[21] C. Goldin, The Political Economy of Immigration Restriction in the United States, 1890 to 1921 (University of Chicago Press, 1994), pp. 223–258.

[22] S. M. Lundberg, S. I. Lee, “A unified approach to interpreting model predictions” in Advances in Neural Information Processing Systems 30 (NIPS 2017). I. Guyon ., Eds. (Curran Associates, Inc., 2017), pp. 4765–4774.

[23] R. Benson, Shaping Immigration News: A French-American Comparison (Cambridge University Press, New York, 2013).

[24] J. M. Eberl ., The European media discourse on immigration and its effects: A literature review. Ann. Int. Commun. Assoc. 42, 207–223 (2018).

[25] O. Santa Ana, ‘Like an animal I was treated’: Anti-immigrant metaphor in US public discourse. Discourse Soc. 10, 191–224 (1999).

[26] L. E. Salyer, Laws Harsh As Tigers: Chinese Immigrants and the Shaping of Modern Immigration Law (University of North Carolina Press, Chapel Hill, 2000).

[27] B. Lew-Williams, The Chinese Must Go: Violence, Exclusion, and the Making of the Alien in America (Harvard University Press, Cambridge, MA, 2021).

[28] S. Iyengar, Y. Lelkes, M. Levendusky, N. Malhotra, S. J. Westwood, The origins and consequences of affective polarization in the United States. Annu. Rev. Polit. Sci. 22, 129–146 (2019).

[29] N. Dias, Y. Lelkes, The nature of affective polarization: Disentangling policy disagreement from partisan identity. Am. J. Polit. Sci. 66, 775–790 (2022).

[30] A. Jensen, W. Marble, K. Scheve, M. J. Slaughter, City limits to partisan polarization in the American public. Polit. Sci. Res. Methods 9, 1–19 (2021).

[31] J. N. Druckman, E. Peterson, R. Slothuus, How elite partisan polarization affects public opinion formation. Am. Polit. Sci. Rev. 107, 57–79 (2013).

[32] N. E. Leonard, K. Lipsitz, A. Bizyaeva, A. Franci, Y. Lelkes, The nonlinear feedback dynamics of asymmetric political polarization. Proc. Natl. Acad. Sci. U.S.A. 118, e2102149118 (2021). 34876513 10.1073/pnas.2102149118 PMC8685731

[33] C. A. Bail ., Exposure to opposing views on social media can increase political polarization. Proc. Natl. Acad. Sci. U.S.A. 115, 9216–9221 (2018). 30154168 10.1073/pnas.1804840115 PMC6140520

[34] R. Axelrod, J. J. Daymude, S. Forrest, Preventing extreme polarization of political attitudes. Proc. Natl. Acad. Sci. U.S.A. 118, e2102139118 (2021). 34876506 10.1073/pnas.2102139118 PMC8685667

[35] A. J. Stewart, J. B. Plotkin, N. McCarty, Inequality, identity, and partisanship: How redistribution can stem the tide of mass polarization. Proc. Natl. Acad. Sci. U.S.A. 118, e2102140118 (2021). 34876507 10.1073/pnas.2102140118 PMC8685720

[36] N. D. Genova, The legal production of Mexican/migrant “illegality”. Lat. Stud. 2, 160–185 (2004).

[37] M. M. Ngai, Impossible Subjects: Illegal Aliens and the Making of Modern America—Updated Edition (Princeton University Press, Princeton, NJ, 2014).

[38] J. R. García, Operation Wetback: The Mass Deportation of Mexican Undocumented Workers in 1954 (Greenwood Press, Westport, CT, 1980).

[39] J. Nevins, Searching for security: Boundary and immigration enforcement in an age of intensifying globalization. Soc. Justice 28, 132–148 (2001).

[40] Gallup, Immigration (Gallup, Inc., 2021). https://news.gallup.com/poll/1660/immigration.aspx. Accessed 1 October 2021.

[41] H. Tessler, M. Choi, G. Kao, The anxiety of being Asian American: Hate crimes and negative biases during the COVID-19 pandemic. Am. J. Crim. Justice 45, 636–646 (2020). 32837158 10.1007/s12103-020-09541-5 PMC7286555

[42] A. R. Gover, S. B. Harper, L. Langton, Anti-Asian hate crime during the COVID-19 pandemic: Exploring the reproduction of inequality. Am. J. Crim. Justice 45, 647–667 (2020). 32837171 10.1007/s12103-020-09545-1 PMC7364747

[43] B. Vidgen ., “Detecting East Asian prejudice on social media” in Proceedings of the Fourth Workshop on Online Abuse and Harms. S. Akiwowo, B. Vidgen, V. Prabhakaran, Z. Waseem, Eds. (Association for Computational Linguistics, 2020), pp. 162–172.

[44] M. Gentzkow, J.M. Shapiro, M. Taddy, Congressional Record for the 43rd-114th Congresses: Parsed Speeches and Phrase Counts (Stanford Libraries, Palo Alto, CA, 2018). https://data.stanford.edu/congress_text/. Accessed 9 June 2021.

[45] N. Judd, D. Drinkard, J. Carbaugh, and L. Young. congressional-record: A parser for the Congressional Record (2017). https://github.com/unitedstates/congressional-record/. Accessed 1 August 2021.

[46] S. B. Carter ., Eds., Historical Statistics of the United States (Cambridge University Press, Cambridge, United Kingdom, 2006). https://hsus.cambridge.org/. Accessed 1 October 2021.

[47] Migration Policy Institute, Countries of birth for U.S. immigrants, 1960-present. (2021). https://www.migrationpolicy.org/programs/data-hub/charts/immigrants-countries-birth-over-time. Accessed 16 June 2021.

[48] Y. Luo, D. Card, D. Jurafsky, “Detecting stance in media on global warming” in Findings of the Association for Computational Linguistics: EMNLP 2020. T. Cohn, Y. He, Y. Liu, Eds. (Association for Computational Linguistics, 2020), pp. 3296–3315.

[49] Y Liu, ., *RoBERTa: A robustly optimized BERT pretraining approach.* arXiv [Preprint] (2019). https://arxiv.org/abs/1907.11692. Accessed 1 October 2021.

[50] C. Haynes, J. L. Merolla, S. K. Ramakrishnan, Framing Immigrants: News Coverage, Public Opinion, and Policy. (Russell Sage Foundation, New York, NY, 2016).

[51] J. Mendelsohn, Y. Tsvetkov, D. Jurafsky, A framework for the computational linguistic analysis of dehumanization. Front Artif Intell 3, 55 (2020). 33733172 10.3389/frai.2020.00055 PMC7861242

[52] J. Devlin, M. W. Chang, K. Lee, K. Toutanova, “BERT: Pre-training of deep bidirectional transformers for language understanding” in Proceedings of the 2019 Conference of the North American Chapter of the Association for Computational Linguistics: Human Language Technologies, Volume 1 (Long and Short Papers). J. Burstein, C. Doran, T. Solorio, Eds. (Association for Computational Linguistics, 2019), pp. 4171–4186.

[53] D. Card, A. E. Boydstun, J. H. Gross, P. Resnik, N. A. Smith, “The media frames corpus: Annotations of frames across issues” in Proceedings of the 53rd Annual Meeting of the Association for Computational Linguistics and the 7th International Joint Conference on Natural Language Processing (Volume 2: Short Papers). C. Zong, M. Strube, Eds. (Association for Computational Linguistics, 2015), pp. 438–444.

[54] J. B. Lewis ., Voteview: Congressional roll-call votes database (2022). https://voteview.com. Accessed 18 March 2022.

[55] Roper Center for Public Opinion Research. 2003. Roper iPoll. https://ropercenter.cornell.edu/ipoll/. Accessed 26 March 2022.

[56] S. Ruggles, S. Flood, R. Goeken, M. Schouweiler, M. Sobek. IPUMS USA: Version 12.0. (2022). https://www.ipums.org/. Accessed 30 March 2022.

[57] D. Card ., “Replication code and data for ‘Computational analysis of 140 years of US political speeches reveals more positive but increasingly polarized framing of immigration’ [dataset].” (2022). https://github.com/dallascard/us-immigration-speeches/. Deposited 8 July 2022. 10.1073/pnas.2120510119 PMC9351383 35905322

