# Supplementary material for: Computational analysis of 140 years of US political speeches reveals more positive but increasingly polarized framing of immigration
Source: Proc Natl Acad Sci U S A. 2022 Jul 29;119(31):e2120510119. doi: 10.1073/pnas.2120510119 (PMC9351383; doi:10.1073/pnas.2120510119)
Supplement: Supplementary File [file pnas.2120510119.sapp.pdf]

Supplementary Information for

Computational Analysis of 140 Years of U.S. Political Speeches Reveals More Positive but Increasingly Polarized Framing of Immigration

Dallas Card, Serina Chang, Chris Becker, Julia Mendelsohn, Rob Voigt, Leah Boustan, Ran Abramitzky, Dan Jurafsky

Dallas Card.
E-mail: dalc@umich.edu

This PDF file includes:

Supplementary text
Figs. S1 to S35 (not allowed for Brief Reports)
Tables S1 to S12 (not allowed for Brief Reports)
SI References

Supporting Information Text

This document contains additional details on data processing, along with additional figures, analyses, and validity checks.

Accompanying Replication Code and Data

Code and data sufficient for replicating the analyses and figures in the main paper, along with code for processing original raw data, and how to obtain it, are available at https://github.com/dallascard/us-immigration-speeches/releases/tag/v1.0

Details of Data Processing

For the Gentzkow et al data (1), all immigration speeches were tokenized with spaCy (https://spacy.io/). Because commas have been converted into periods in the raw data, we rejoin mistakenly split sentences by re-attaching any sentence that begins with a lower case letter to the preceding sentence, if it ends in a period. We also exclude all speeches listed as being from June 14, 1894, as many of these were found to be modern speeches that have been mistakenly included among the speeches from that date. Most speeches in the dataset include a named speaker, state, and party, though about 15% of speeches are missing this information, most of which are procedural (e.g., "Without objection it is so ordered").

For the speeches from the @unitedstates project (https://github.com/unitedstates/congress), we also tokenize them using spaCy, and then apply pre-processing to more closely match the format of the Genzkow data. To do this, we replace all commas with periods, drop all apostrophes, and remove all hyphens connecting words.

For the Presidential data, we first split each document into paragraphs (by splitting on newlines), and treat each paragraph as a segment. Some documents (such as transcripts of press briefings) include comments from multiple speakers. To exclude speeches by everyone except The President, we filter out blocks of text that begin with "Q." or "Q:" (question) as well as other named positions (e.g., "The Vice President:") and those that begin with a name preceded by a non-Presidential title (e.g., "Dr. [NAME]:"). For additional details, please refer to online replication code.

Although the Congressional Record data is generally of high quality, there are some errors from Optical Character Recognition (OCR), especially in earlier years. Table S1 shows, for three key terms, the most common similar tokens found in the first part of this corpus (sessions 46-70) (those that have an edit distance of one to the target). The counts of obvious OCR errors are relatively small, but we nevertheless include some common variations (e.g., "inmigration", "imnigration") as keywords when doing the initial speech selection for annotation.

Table S1. Common OCR errors for three key terms. The table shows the token counts for the most common terms with an edit distance of one from the target terms (top row) in all speeches from the 46th to 70th Congress, revealing that OCR errors are present, but relatively rare.

Term	Count	Term	Count	Term	Count
immigration	25234	chinese	17932	mexican	14868
inmigration	138	chinee	41	mexicans	1533
imnigration	86	chines	30	merican	100
imigration	48	-chinese	17	exican	37
imnmigration	43	chineso	15	mlexican	28
immnigration	38	chinese-	13	lexican	14
inmmigration	27	hinese	12	miexican	12

Details of Annotations for Relevance and Tone

In order to get a sufficiently large number of positive examples for annotation, initial keyword filters were used to identify sentences in the Congressional speeches that were potentially about immigration. These were developed through an iterative process of keyword selection, query expansion, and exploration, and encompassed prefixes relating to immigration (e.g,. "immig"), regions and nationalities (e.g., "irish"), labor (e.g., "cooly"), naturalization (e.g., "visa"), and related terms, as well as some specific keywords and bigrams (e.g. "I.N.S.", "foreign workers", "ellis island", etc.). Three separate but overlapping lists were used for three phases of immigration: early (1873-1934), mid (1935-1956), and modern (1957-2020). For the full list of terms, please refer to online replication code.

After identifying relevant sentences with keywords, segments consisting of the sentence, along with the preceding and following three sentences, were exported and aggregated into batches, which were randomly assigned to annotators, such that each segment was assigned to at least two annotators, and approximately equal numbers of sentences were were annotated by all possible pairs of annotators. Annotators read the passages and rated them as relevant to immigration or not (yes/no), and for the relevant segments, provided a judgement as to the overall tone of the passage (pro-immigration, anti-immigration, or neutral). Annotators also provided open ended free-text responses about how immigrants were being characterized in each passage, if applicable. Critically, the same set of five annotators provided the annotations for all annotated segments, meaning that consistent notions of relevance and tone were applied to the whole corpus. The numbers of annotated examples are given in the Table S2 below.

Although it is difficult to summarize all position on immigration using a simple ternary categorization scheme, (for example, someone might favor increased immigration, but oppose immigration from particular countries), annotators were told to

Table S2. Number of annotated segments for each time period and task. Note that most examples were annotated by at least two annotators. Annotations from the middle period were only used for evaluation. In addition, modern tone annotations were augmented with tone annotations from the Media Frame Corpus (2).

Label	Early (1873-1934)	Mid (1935-1956)	Modern (1957-2020)
Relevance	3786	1440	2400
Tone	1995	521	1127

treat the pro-immigration position as being favorable towards immigrants and continued or increased immigration, and anti-immigration as being unfavorable towards immigrants or favoring greater restrictions on immigration. For the purpose of relevance judgements, immigrants were considered to be any foreign born people residing temporarily or permanently in the United States, excluding discussions of internal migration, American Indians or Indigenous peoples, African slavery, or movement to or from U.S. territories and protectorates.

The chance-corrected agreement rates among the annotators for each task and subset of the data are give in table S3 below, measured using Krippendorff's alpha (which produces values in the range $[-1, 1]$, with $\alpha = 0$ indicating agreement at the level of chance. As can be seen, agreement rates are very strong for relevance, and somewhat weaker for tone, revealing that it is a more challenging task. In addition, agreement rates are broadly similar across the three time periods, indicating that the annotators did not have substantially greater difficulty annotating the tone of segments from the early time period, despite having less familiarity with the language and issues of the day.

Table S3. Chance corrected agreement rates, measured using Krippendorff's alpha, for each of the three subsets of data and two tasks, showing strong agreement for relevance, and somewhat weaker agreement for tone (1 = perfect agreement; 0 = agreement at the level of pure chance).

Label	Early (1873-1934)	Mid (1935-1956)	Modern (1957-2020)
Relevance	0.77	0.80	0.71
Tone	0.43	0.49	0.51

To aggregate the multiple annotations per segment from the annotators, we make use of a Bayesian item-response style model, which models latent item labels and latent annotator biases (see Appendix of (3)). This model simultaneously infers a probability distribution over labels (e.g., yes/no, or pro/neutral/anti) for each item and biases for each annotator, allowing us to determine the appropriate label in cases where annotators disagree. We first aggregated the relevance judgements to select all annotated segments deemed relevant. For that subset, we similarly infer a distribution over tone labels (pro, neutral, or anti). This was done separately for the three slices of annotations: early (1873-1934), mid (1935-1956), and modern (1957-2020).

The average tone by party using only the inferred labels from the annotated segments for the three time period is shown in Figure S1 below, revealing that the same trends we observe in Figure 1 (in the main paper) are also reflected in the annotated data (though with much less precision, due to the limited amount of data). As can be seen, we also annotated more data from the earliest time period, to help account for possible unfamiliarity with the language and issues of the time.

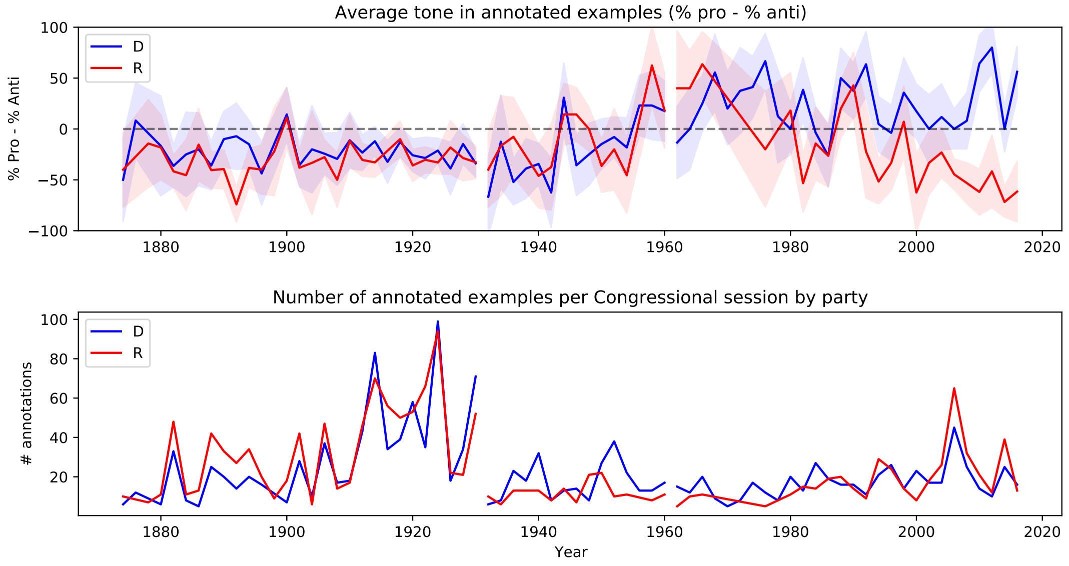

Fig. S1. Average tone (% pro - % anti) using only the annotated segments for three time periods (top), and the number of segments annotated per session of Congress (bottom), divided by party in both cases.

Details of Training and Applying Classifiers for Relevance and Tone

Using the inferred labels from the annotated segments, we then trained a pair of classifiers for both the early and modern time periods, which we chose to focus on due to the relatively small amount of immigration to the U.S. in the intervening years. For both time periods, we trained a binary classifier for relevance, and a ternary classifier for tone. To do so, we built on the `transformers` library, beginning with the pretrained `roberta-base` model provided by Hugging Face (https://huggingface.co/). We implemented a weighted classifier that incorporates the inferred label probabilities into the cross entropy loss as weights during training. For the tone classifier for the modern time period, we also augmented the annotations we collected with similar tone annotations from the immigration news articles in the Media Frames Corpus (2), which resulted in a slight increase in held out performance.

To identify relevant speeches in the full corpus, we first applied the relevance and tone classifiers to all segments which contained keywords (the pool of candidate segments for annotations). A second, smaller set of speeches was identified by identifying speeches not yet identified as being about immigration, breaking them into segments, classifying all of those segments as being relevant or not, and keeping only those speeches that contained segments classified as relevant and occurred on a day with many speeches already classified as being about immigration (see replication code for details). In this way, the relevant speeches primarily consisted of those that contained keywords, but were not limited to those.

The overall tone of each speech is obtained by counting the number of segments in a speech classified as being pro, neutral, or anti (among those classified as being relevant to immigration). In other words, although we count entire speeches which only briefly mention immigration as being *relevant* to the issue, the *tone* assigned to each speech is determined only by those parts of the speech which are judged relevant, not the unrelated parts.

For the early and later periods, we only use the predicted labels from the corresponding models. To get final labels for segments from the middle period, we used a linear interpolation of the predicted probabilities from the two models, placing all the weight on the early model in 1934 and transitioning to placing all the weight on the modern model by 1957.

To confirm that these models are comparable, we inspect the aggregate predictions made by each model individually on all of these years, as shown below. We also plot the predictions using both the Gentzkow et al data and the @unitedstates project data for the years where they overlap (the 104th to the 112th Congress) to confirm that the slight differences between these data sources are not consequential. As shown in Figures S2 and S3 below, there is nearly perfect overlap for the later models (for both relevance and tone) for the comparison between data sources, demonstrating that the minor differences between these sources are not consequential. By contrast, the difference between Model 1 and Model 2 is slightly larger. This makes sense however, given that the content of speeches about immigration changed dramatically over this time, hence the use of two different models. Nevertheless, the earliest aggregate predictions of the later model agree strongly with those of the earlier model, implying that the predictions from these two models are indeed comparable.

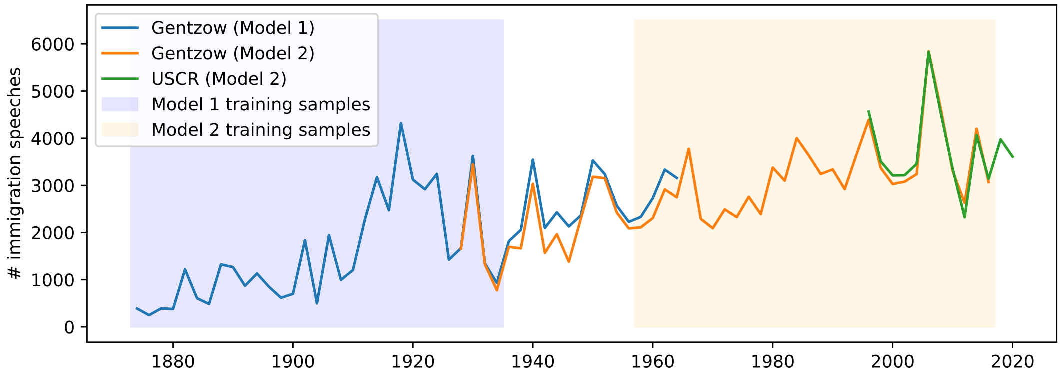

Fig. S2. Aggregate relevance predictions from the two relevance models, on both the full Gentzkow et al data and the @unitedstates project data (USCR). The blue and orange shaded areas show the data sampling periods for the first and second model, respectively. The blue and orange lines show the number of segments classified as being about immigration (per Congress) on the Gentzkow et al data by the first and second models, respectively. The green line shows the same for the predictions of the later model on the @unitedstates project data. As can be seen, there is strong agreement between these three lines, indicating that a) the two models are comparable to each other, and b) the two data sources are comparable to each other.

Overall performance estimates are provided in Tables S4 and S5 below, both as confusion matrices and overall accuracy. For the early and later time periods, we report numbers using cross-fold validation, averaging over multiple random seeds. For the middle period (which was not used for training), we simply report numbers from the final predictions of our classifiers on all annotated segments.

Accuracy on predicting relevance of immigration segments is consistently about 90% for all three time periods, including for the middle period, which was not used for training. The accuracy for identifying tone is lower, not surprisingly, as this is both a harder task, and there is less labeled data available (only the annotated segments labeled as relevant). Nevertheless, performance is again fairly consistent across time periods, achieving approximately 65% accuracy, including during the middle period. More importantly the rate of misclassifying pro-immigration segments as anti-immigration segments, and vice versa, is low, meaning that the errors will be of degree rather than kind.

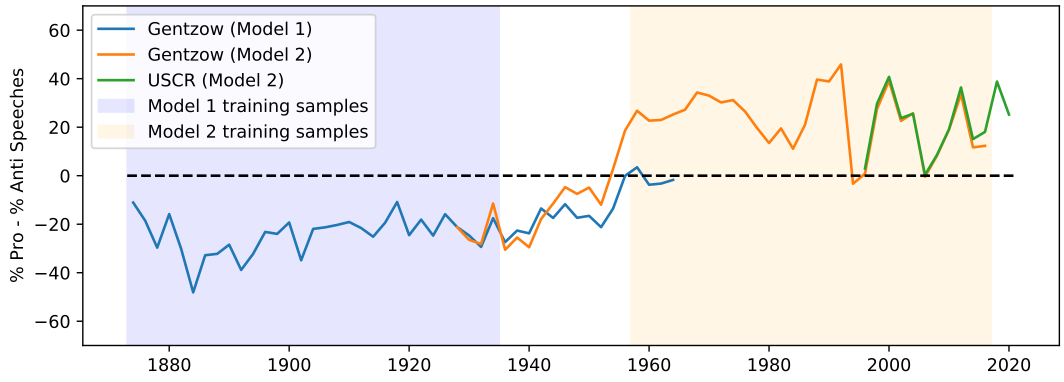

Fig. S3. Aggregate tone predictions from the two tone models, on both the full Gentzkow et al data and the @unitedstates project data (USCR). As above (Figure S2), there is near perfect agreement between the two data sources. The agreement between the two models in the middle period is slightly weaker than for relevance, but the aggregate predictions still show the same trends. In particular, the prediction of the later model match the earlier model almost perfectly at the beginning of the transition period. By contrast, the latest predictions of the earlier model are not as positive as those made by the later model, likely because there are aspects of positive immigration language in the 1960s that the earlier model was not exposed to in the data it was trained on.

In order to ensure that our results are not being excessively influenced by underlying biases in the base RoBERTa model, or differential classifier performance, we include a series of validation checks, using alternative model specifications and aggregate corrections (see Validity Checks for Tone below).

Table S4. Estimates of performance for identifying speeches as being relevant to immigration, for early, middle, and modern time periods, shown as confusion matrices and overall accuracy.

	**True / Pred**	No	Yes	**Accuracy**
	No	0.60	0.06	
**Early**	Yes	0.03	0.30	
	**Accuracy**			0.91
	**True / Pred**	No	Yes	**Accuracy**
	No	0.64	0.09	
**Middle**	Yes	0.01	0.26	
	**Accuracy**			0.90
	**True / Pred**	No	Yes	**Accuracy**
	No	0.47	0.07	
**Modern**	Yes	0.05	0.40	
	**Accuracy**			0.88

Table S5. Estimates of performance for classifying the tone of immigration speeches, for early, middle, and modern time periods, shown as confusion matrices and overall accuracy.

	**True / Pred**	Anti	Neutral	Pro	**Accuracy**
	Anti	0.26	0.10	0.03	
**Early**	Neutral	0.13	0.31	0.05	
	Pro	0.03	0.03	0.06	
	**Accuracy**				0.63
	**True / Pred**	Anti	Neutral	Pro	**Accuracy**
	Anti	0.23	0.08	0.02	
**Mid**	Neutral	0.08	0.31	0.04	
	Pro	0.02	0.07	0.15	
	**Accuracy**				0.69
	**True / Pred**	Anti	Neutral	Pro	**Accuracy**
	Anti	0.24	0.08	0.03	
**Modern**	Neutral	0.06	0.17	0.08	
	Pro	0.02	0.06	0.26	
	**Accuracy**				0.67

Identifying Procedural Speeches

Many of the speaking turns in Congress are pro forma, such as "Without objection it is so ordered". However, there is no strict definition of what counts as a "procedural speech", and at times the pro forma language appears in slightly modified form, or occurs in combination with more substantive speech. Although most such speeches are unlikely to be classified as being relevant to immigration, we still endeavor to exclude procedural speeches, to avoid misleading estimates (especially for the overall prevalence of immigration speeches). To do so, we work on the assumption that short speeches which are repeated verbatim or nearly verbatim many times should be treated as procedural.

To identify and exclude such speeches, we first exclude all speeches shorter than three words or sixteen characters (e.g., "nay"). We then train a regularized logistic regression classifier, representing text as the binarized counts of unigrams, bigrams, and trigrams (e.g. "is so ordered"). To obtain training data for such a classifier, we first identify short speeches that are repeated verbatim many times. We first simplify each speech by dropping punctuation, converting to lowercase, and converting all gaps between tokens to single spaces. We then identify speeches shorter than 200 characters, and keep those that are repeated verbatim at least 20 times as positive examples of procedural speeches, and put a random 10% of these into the training data. (We only use 10%—giving each unique text string an equal chance of being selected, no matter how many times it occurs—due to the massive number of such speeches). As negative examples, we take all speeches with more that 1000 characters (and more than twenty tokens), and break them into pieces of approximately 200 characters. We select a random 5% of these as non-procedural test instances, and add the rest to the training data. To create procedural examples for the test set, we count all speeches that occur verbatim at least ten but less than twenty times, and add each such unique speech as a procedural example to the test set.

Using this training data, we then train an L1-regularized logistic regression model, keeping all ngrams (for n equals 1, 2, or 3) that occur at least twice, choosing the regularization strength using 5-fold cross validation. The resulting model obtains over 99% accuracy and over 99% F1 on the held-out test set described above. We then apply this classifier to all speeches shorter than 400 characters, and exclude all speeches classified as procedural (approximately 8 million speeches, or 45% of the speeches in the full corpus).

Frequency of Immigration Speeches

Figure S4 shows how common the topic of immigration is over time, both in Congress (top), and in Presidential communications (bottom). The two trajectories are broadly similar, with immigration being mentioned much more under Presidents George W. Bush, Barack Obama, and Donald Trump, compared to previous Presidencies. The frequency with which it is discussed is very similar between the two parties, though Republicans spoke much more about it under President Bush, and Democrats spoke much more about it under President Trump. President Trump himself mentioned immigration far more frequently than any previous President, especially in 2017 and 2018. Overall, immigration is an even more salient issue today than it was during the debate around immigration quotas in the 1920s.

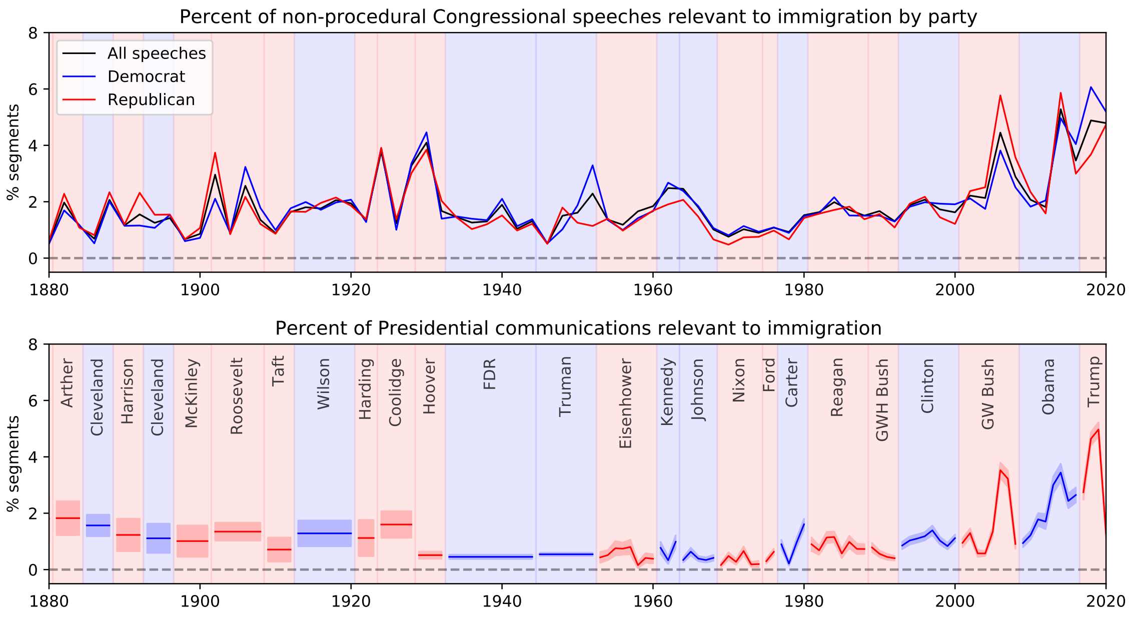

Fig. S4. Frequency of immigration speeches: in Congress (top), and by Presidents (bottom). The Presidential plots shows the percent of paragraphs classified as being about immigration (because the raw data is conveniently broken into paragraphs), whereas the Congressional plots show this as the approximate percentage of speech segments about immigration, which is estimated by dividing the total number of tokens in speech segments classified as being about immigration by the total number of tokens in each session of Congress, both overall and separately by party.

Validity Checks for Tone

Linear Models. Classifiers based on pre-trained models, such as RoBERTa, typically perform better, but introduce some amount of unknown bias (from the pre-training data). To verify that our results are not being excessively influenced by `roberta-base`, we repeat our pipeline using basic logistic regression models, operating on bag-of-words features (which avoids all issues with biases from pretraining data) and reproduce Figure 1 in the main paper based on the predictions from these simpler classifiers.

For the sake of simplicity, and to simultaneously address concerns about our use of separate models for the earlier and later parts of our data, we do this replication using only a single model for relevance, and a single model for tone. That is, we combine all annotated data, leaving out a random set of 400 segments for each, and train one logistic regression model on the combined relevance annotations, and another on the combined tone annotations. Because data is limited, we do not do extensive tuning of these models. Rather, we use what are known to be strong default choices (4): we use all unigrams and bigrams that occur at least twice in the training data, binarize all features (present or absent), and regularize the model using L1-regularization, with strength tuned using five-fold cross validation. The resulting models have similar accuracy for relevance (0.89), but slightly lower accuracy for tone (0.63). Nevertheless, the resulting time series for Congressional Speeches using the logistic regression models, shown in Figure S5, is overall extremely similar to the one based on the contextual embedding models (Figure 1 in main paper). The differences for the Presidential time series are somewhat more pronounced, though we still find that modern presidents are generally more positive than those in the past, with President Trump again being a stark outlier.

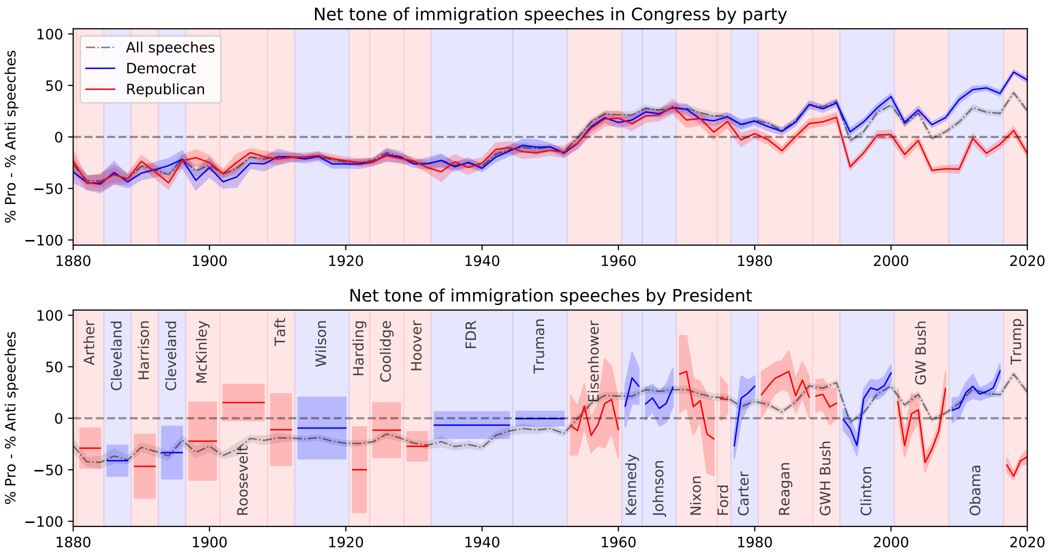

Fig. S5. Replication of Figure 1 in the main paper (average tone over time for Congress and Presidents in the top and bottom sub-panels, respectively), using logistic regression models for relevance and tone (based on unigram and bigram features), rather than models based on `roberta-base`. As can be seen, results are extremely similar to the main results in terms of all important findings.

We similarly use these models to recreate Figure 2 in the main paper, shown in Figure S6, again finding no meaningful difference from the models based on `roberta-base`, which helps to assuage concerns that biases present in the RoBERTa models could be distorting our results. (Note that for Figure S6, because we only plot sessions with at least 20 relevant speeches, there are some differences in terms of which sessions are plotted, due to slight differences in which speeches are classified as relevant).

Binary Tone. In order to address concerns about the fluctuating prominence of the "neutral" category, as well as the difficulty in interpreting our main measure of tone (% pro - % anti), we train a separate tone model, again based on `roberta-base`, but using only the annotated segments that were labeled as pro- or anti-immigration (excluding neutral). For simplicity, we train a single binary tone model for the entire time series, and then use it to classify the entire corpus, for both Congressional and Presidential speeches.

The results from this model are shown in Figure S7, plotted as the percentage of speeches over time (per party) classified as pro-immigration. Although there are some subtle differences from the figure in the main paper (specifically a greater degree of negativity in the past relative to the present), overall the patterns are remarkably similar. We generally consider this figure to be less meaningful than the results in the main paper, since it represents a mismatch between the predictions and the original annotation scheme, but the similarity of results again helps to bolster confidence in our findings.

Correcting for Tone Error Rates. To address concerns about the accuracy of our tone classifiers, and the possibility that systematic errors could be distorting our results, we make use of a correction based on Bayesian inference, which accounts for effects of both time period and party. To do so, we first estimate the prior probability of each tone label (pro, neutral, or anti) for each party in each Congressional session. This is done by averaging the labels provided by annotators for all segments

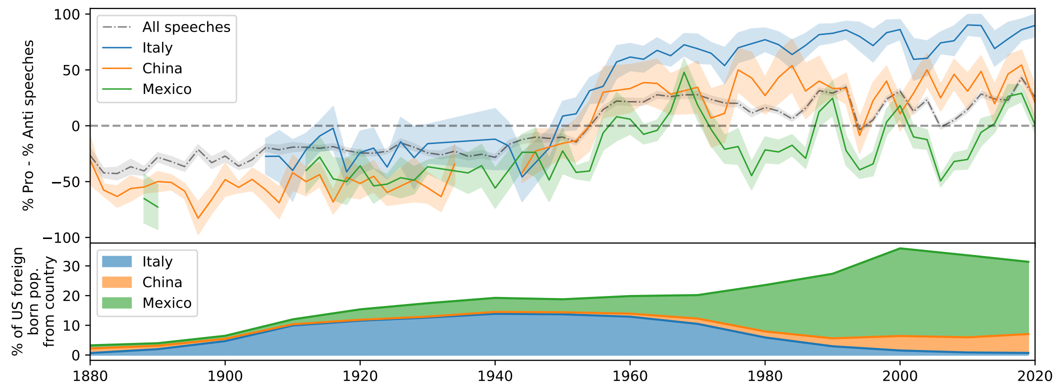

Fig. S6. Replication of Figure 2 in the main paper (average tone over time for the most frequently mentioned nationalities), using logistic regression models for relevance and tone (based on unigram and bigram features), rather than models based on `roberta-base`.

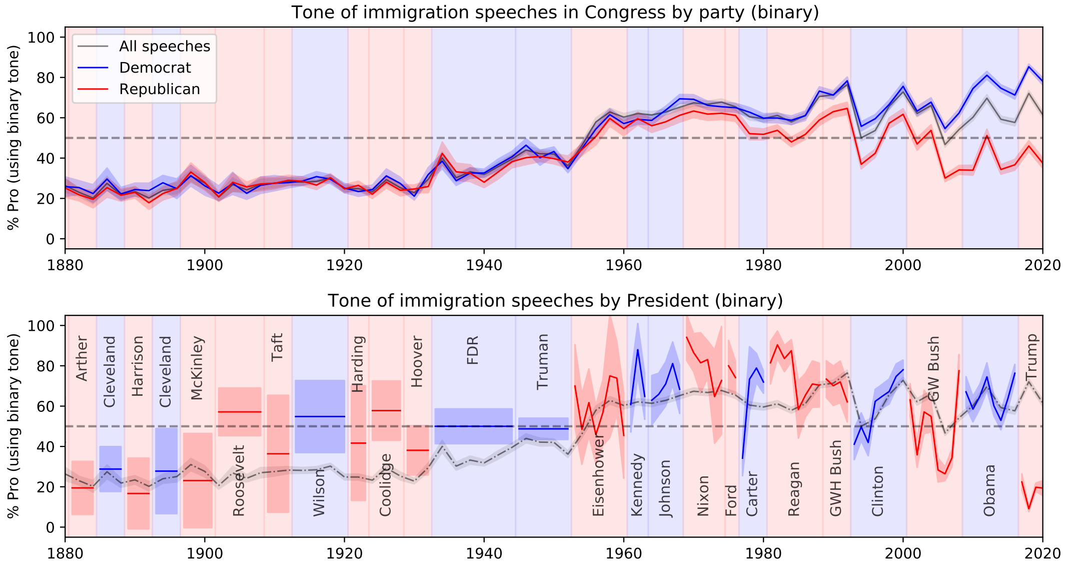

Fig. S7. Alternative to Figure 1 in the main paper using a different RoBERTa model, trained only on pro- and anti-immigration speeches, to predict tone as a binary variable. The y-axis shows the percentage of speeches per Congress classified as pro-immigration.

from within the previous or following 10 sessions of Congress, and gives us a prior label distribution per session and party, based only on the raw annotated data, i.e., $p(y \mid P, C)$, where y is the true (human-identified) tone of a speech, P is the party (Republican or Democrat), and C is the session of Congress (43–116).

We then use the predictions of our tone models to estimate confusion matrices for tone predictions, similar to what is shown in Table S5, but normalized by row, and computed per-party for the modern model.* This gives us an estimate of the probability of each predicted label, conditional on each true label, with estimates that vary depending on whether the Congressional session is from the early, mid, or modern period of annotations.† We denote these values as $p(\hat{y} \mid y, P, C)$, where $\hat{y}$ is the predicted label, and y is the true label (provided by the annotators). Because what we actually want is the probability of a true label, conditional on a predicted label (provided by the models), we invert the confusion matrix using Bayes' rule, i.e.,

$$p(y \mid \hat{y}, P, C) \propto p(\hat{y} \mid y, P, C) \cdot p(y \mid P, C),$$

and normalize by summing over all values for each predicted label.

Putting this all together, we use the inverted confusion matrix to correct the predicted tone probabilities for each segment (which generally softens them away from high probability assigned to a single label), and recreate Figure 1 from the main paper using the corrected probabilities, rather than the predicted labels (i.e., the sum of the pro-immigration probabilities minus the sum of the anti-immigration probabilities). The resulting Figure is shown in Figure S8.

Although there are some minor changes from the original time series, the overall patterns are essentially the same. Regardless, we treat this a supporting validity check, rather than our main result, in part due to potential error in estimating error rates

*For the early model, we just use a single estimate, since there is so little difference between the parties.

†The confusion matrices are not estimated per session of Congress because they require more data than the class priors.

and class priors from limited amounts of annotated data.

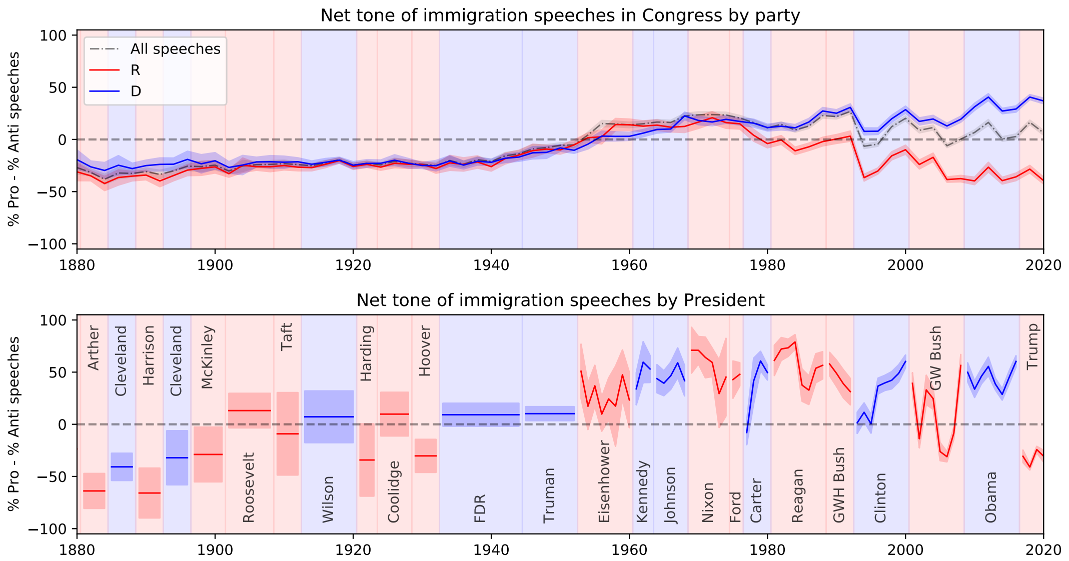

Fig. S8. Alternative to Figure 1 in the main paper, after applying a Bayesian correction to the predicted probabilities, to account for error rates in models and prior label distributions from annotation (by party and over time).

Leave-one-out Analysis. Finally, to ensure that the results are not being strongly influenced by individual members of Congress, we recompute the tone time series from Figure 1 in the main paper, leaving out each speaker in turn (for those with at least 20 immigration speeches). We plot each resulting time series in the bottom half of Figure S9, which shows that nearly all of the resulting time series are virtually identical, indicating that results are not driven by a single influential speaker.

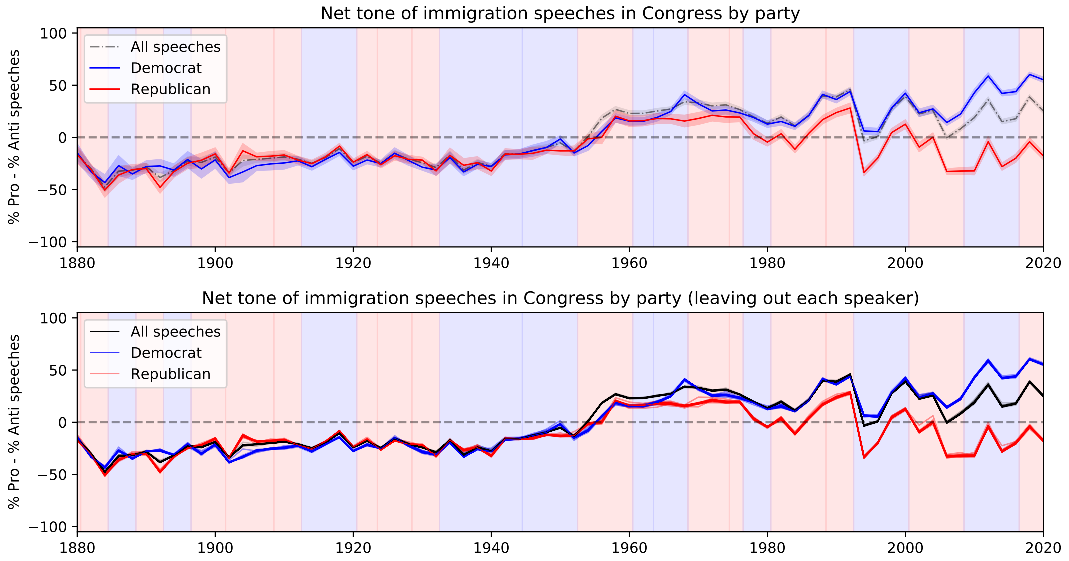

Fig. S9. The original time series from Figure 1 in the main paper (top), and the equivalent results (without bands), when leaving out each speaker in turn, demonstrating that the changes in tone are not being driven by individual members of Congress. Occasional deviations can be seen (e.g., in 2003–2004 among Republicans, where one speaker did have a relatively large negative impact), but mostly the resulting lines are nearly identical.

As a complement to this, Figure S10 shows the pattern in net tone per speaker, for all speakers in sessions of Congress where they have at least 20 immigration speeches. As can be seen, there is considerable variability in tone within each party at all points in time. In addition, we can see the most recent sessions of Congress are unprecedented, in that, except for one or two outliers, the most anti-immigration Democrat is still more pro-immigration than the most pro-immigration Republican. During the Trump administration, we find that all Democrats (among those for whom we have sufficient data), were pro-immigration in their speech, on average, whereas nearly all Republicans were anti-immigration, according to our metric, as was the case for nearly all legislators before the 1950s.

The largest outlier with respect to this finding is the one Republican who appears very pro-immigration in 2017–2018. This point represents Ileana Ros-Lehtinen (R, FL), who was the first Cuban-American elected to Congress, and co-founder of the Bipartisan Congressional Refugee Caucus (5). Although we should not place too much weight on any individual point (due to the limited number of speeches per session of Congress), a pro-immigration estimate for Representative Ros-Lehtinen is not implausible.

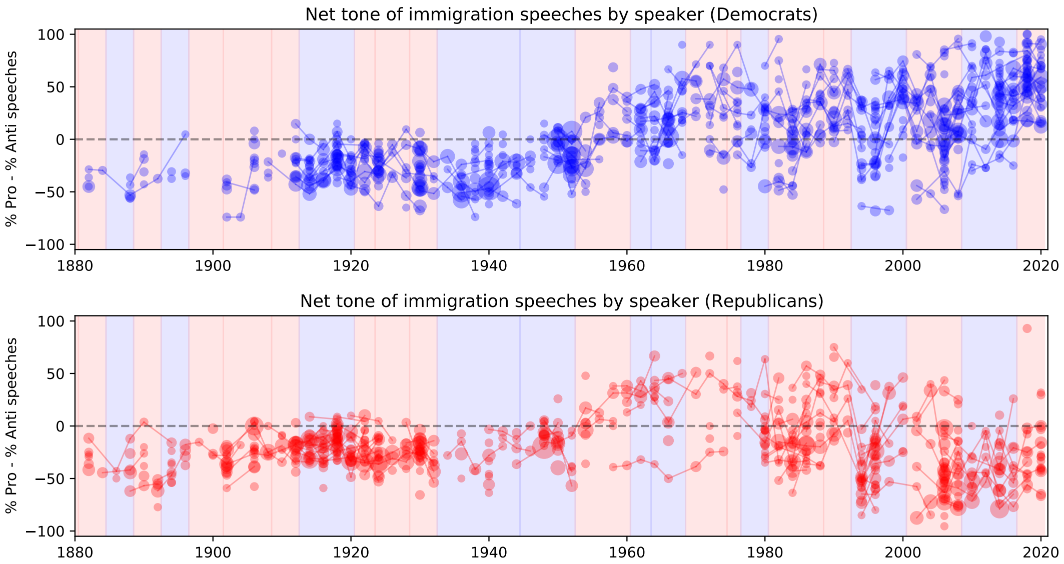

Fig. S10. Tone of immigration speeches by speaker. Each dot shows the net tone of immigration speeches by a speaker for a single session of Congress in which they have at least 20 such speeches, with size indicating the number, and lines connecting the dots for each speaker.

For the most reliable estimates per speaker, Figure S11 shows individual tone estimates (% pro - % anti) for the twenty members of Congress with the most speeches related to immigration. Nearly all of these prolific legislators appear to be close to the overall Congressional average, and in some cases appear to change their expressed attitudes over time, largely in line with evolving trends. The two most dramatic outliers relative to the average appear to be Jeff Sessions—who made an anti-immigration position a key part of his political agenda (6)—and Alan Simpson—co-architect of the 1986 Immigration Reform and Control Act, which targeted employers who hired people without authorization to work in the U.S.

Finally, figure S12 shows the percent of immigration speeches classified as pro, neutral, and anti over time, both overall (top), and by party (Democrats middle and Republicans bottom). As can be seen, most speeches are neutral over most of this time series, though these become less common over time, consistent with greater polarization. By contrast, there is a decline in negative speeches and a rise in positive speeches in the middle of the century, followed by a resurgence in anti-immigration sentiment.

Party, Region, and Chamber Model

Given the uneven geographical nature of immigration to the U.S., as well as regional realignments in party affiliation during this time period, we provide a validity check on party polarization in which we disambiguate the contributions of party and geography to changes in expressed attitudes towards immigration.[‡] To do so, we fit hierarchical Bayesian models to the predicted tone of immigration segments, building linear models with effects per year for each of party and region, as well as overall tone, with each type of offset drawn from its own hierarchical prior, which we fit using Stan (7).

In more detail, we model the tone of each speech as drawn from a normal distribution, with a mean parameter modeled as a linear function of a overall mean tone per Congress, a party offset per Congress (Democrat, Republican, or no party information), a region offset per Congress (North, South, or West), and an offset per Congress for the Senate. Each set of values are drawn from a corresponding normal prior with individual variance parameter (for party, region, and chamber), with weakly informative priors on the variance parameters (For additional details please refer to replication code).

Figure S13 shows our resulting estimate of the tone expressed towards immigration by party (top), with tone scaled between -1 and 1, as well as the estimated difference in party biases (bottom), with bands showing plus or minus 2 standard deviations based on samples from the posterior distribution. As can be seen, after accounting for regional effects, the overall expressed tone towards immigration appears to have been at its most negative extreme during the Congressional session which included 1882 (the year of the Chinese Exclusion Act, renewed in 1902), and remained consistently negative up until World War II. Expressions of pro-immigration tone then increased dramatically from approximately 1940 to the late 1960s, even when accounting for differences between parties, regions, and chambers.

[‡] For example, Chinese immigration to the U.S. during the 19th century was largely concentrated in the West Coast, whereas Cuban refugees have historically had strong connections to Florida; in addition, geographic divisions within the Democratic party over segregation eventually led to a party realignment, with Republicans capturing much of the South over the course of the 20th century.

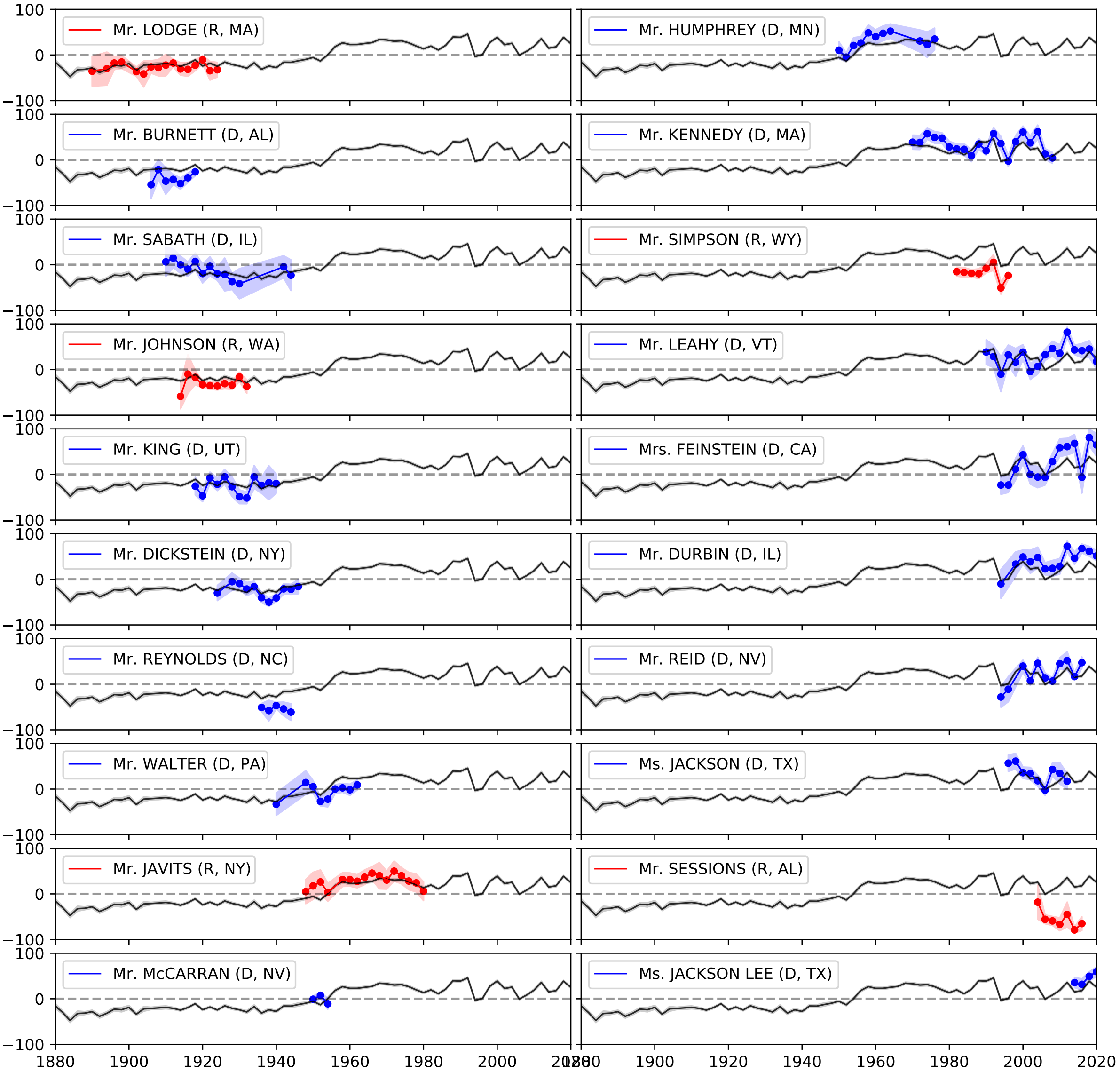

Fig. S11. The estimated attitudes towards immigration (% pro - % anti) of the 20 most prolific members of Congress (in terms of number of speeches) on the topic of immigration, overlaid on the overall trend. As can be seen, most of these mirror the overall trends, and generally remain close to the average attitude in Congress as it develops, with Jeff Sessions and Alan Simpson being the most extreme exceptions.

The attitudes expressed by both parties were pro-immigration on average during the entire 1960s and most of the 1970s, but only the Democrats have maintained a pro-immigration position until the present day. According to the output of this model, a significant difference first appeared between the parties during the 90th Congress (1967/68).[§] The parties then briefly came back into alignment under Nixon, after which the difference in party bias has grown steadily from the late 1970s until the present day, with the Republicans growing increasingly negative towards immigration, relative to the Democrats.

In terms of geography, meanwhile, the estimated biases by region (North, South, and West), are shown in Figure S14. We see that the North has had a consistent mildly pro-immigration bias over this time period, while the South had a relatively anti-immigration bias over the entire twentieth century, and the West has gradually changed from having an anti- to a pro-immigration bias, with a considerable dip from the late 1970s to the early 2000s, especially during the period of discussion of Prop 187 in California. Overall these regional biases pale in comparison, however, to the growing partisan divide shown in Figure S13 (note the difference in scale between the two figures). Finally, Figure S15 shows the estimated offset for the Senate over time, which fluctuates between slightly more pro- and anti-immigration than the House of Representatives, with no consistent difference between them.

The drop in tone coinciding with the start of Bill Clinton's Presidency, which appears for both parties and for Clinton

[§]The largest contributors to the surge in pro-immigration sentiment among Democrats at this time were Senator Ralph Yarborough of Texas, a member of the progressive wing of the Democratic party who spoke extensively on behalf of Mexican Americans, and Representative Leo Ryan of California, who spoke positively about Irish and Italian immigrants, arguing in favor of citizenship for veterans.

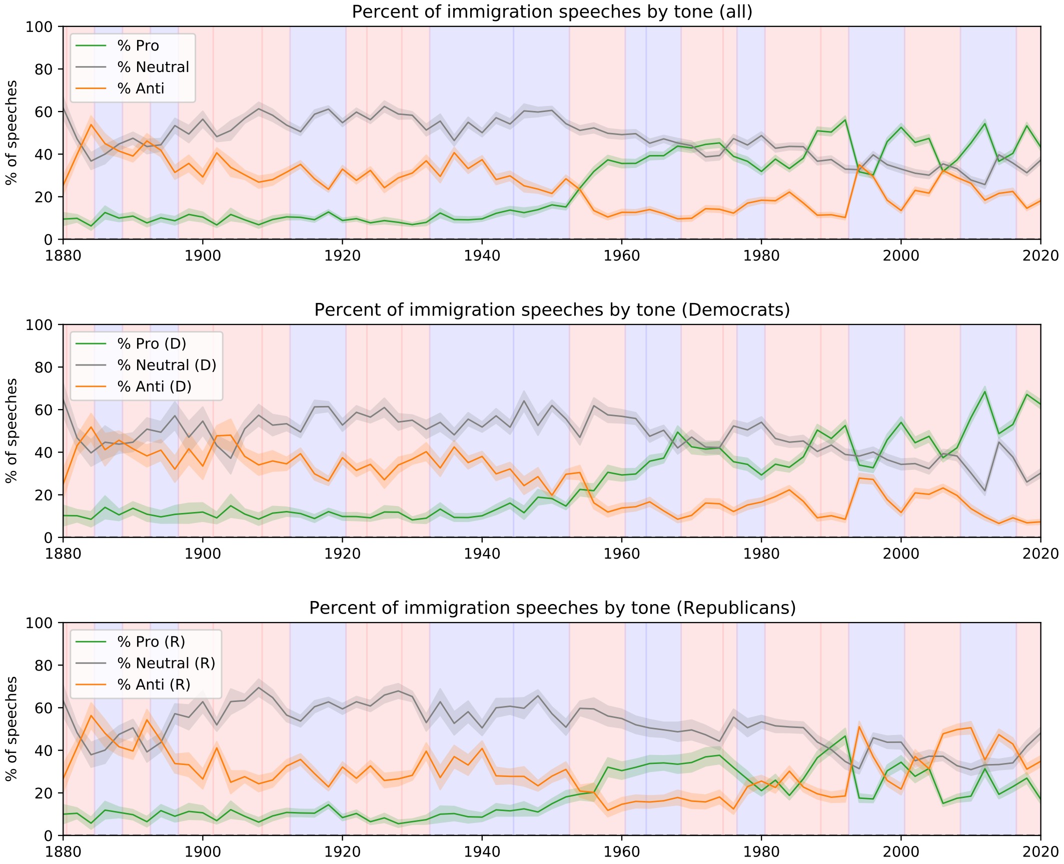

Fig. S12. Estimated percent of speeches related to immigration per year classified as being pro-immigration, neutral, or anti-immigration, plotted as a percentage of all immigration speeches (top), for speeches by Democrats only (middle), or for speeches by Republicans only (bottom).

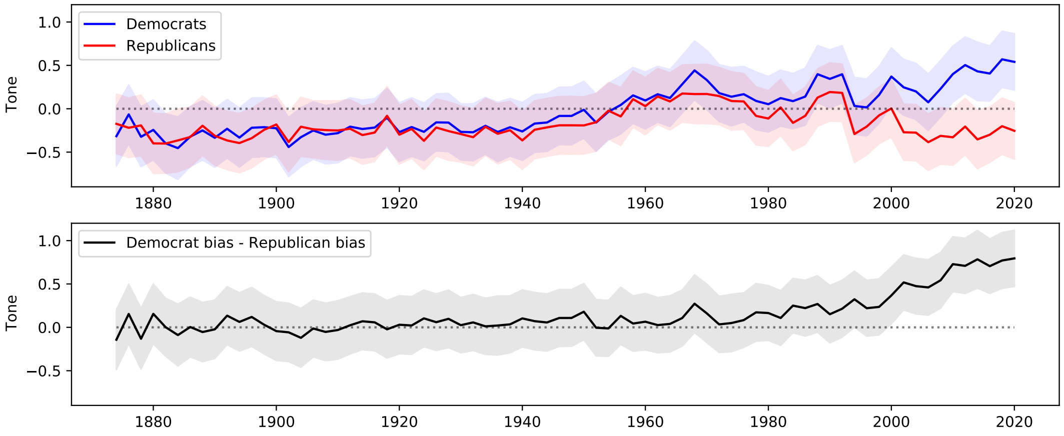

Fig. S13. Estimated tone by party over time (top), and estimated difference in party biases (bottom), after accounting for the effect of geography and chamber, with tone scaled between -1 and 1, and bands showing +/- 2 standard deviations using samples from the posterior distribution.

himself, seems to be a result of the end of the cold war, which meant a decline in sympathetic language in reference to victims and refugees from the Soviet Union, combined with a rise in anti-immigration rhetoric regarding the U.S.-Mexico border. In comparison to the preceding three sessions of Congress, the relatively most frequent terms in the 90th Congress (ignoring person names, dates, and stopwords) are “NAFTA”, “Haiti”, “crime”, “illegal”, “Mexico”, and “border”. By contrast, the most relatively infrequent terms are “Soviet”, “Israel”, “Jews”, “emigration”, “refugee”, and “freedom”.

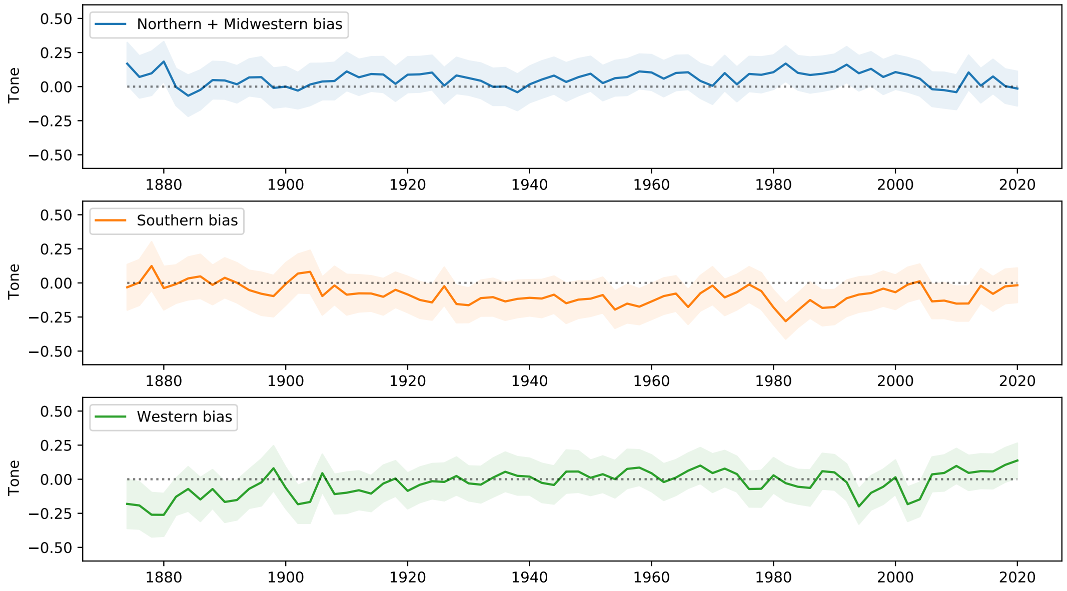

Fig. S14. Estimated regional offsets over time for the North, South, and West, respectively. Northern states are ME, MA, RI, CT, NH, VT, NY, PA, NJ, DE, OH, IN, MI, IL, MO, WI, MN, IA, KS, NE, SD, ND. Southern states are WV, VI, VA, KY, TN, NC, SC, GA, AL, MS, AR, LA, FL, TX, OK, NM, AZ. Western states are CO, WY, MT, ID, WA, OR, UT, NV, CA, AK, HI.

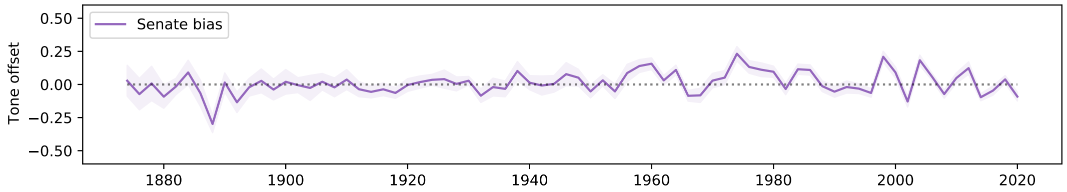

Fig. S15. Estimated Senate offset over time.

Comparison to DW-NOMINATE

The analyses of tone in the main paper primarily emphasize trends over time, and overall differences *between* the parties. It is also interesting, however, to investigate whether our measure of tone captures variation *within* parties.

To do so, we obtained ideological positions for each speaker in Congress using DW-NOMINATE, a widely-used scaling procedure to compare politicians based on their voting records (8). For each member of Congress from 1880–2020, we collected information for the first and second dimension recovered by DW-NOMINATE using Voteview data from March 2022.[¶] The first dimension captures the liberal-conservative spectrum, while the second has historically captured more differences within political parties over regions, civil rights, and lifestyle (9).

We then matched the names from the Voteview database (which are official full names) with the name of the speakers in our immigration speeches dataset. To deal with partial matches (due to partial names and OCR errors), we used the following procedure: first, we normalized all names in both the Voteview and immigration speech records to be all uppercase and converted accents and other diacritics to their ASCII representations. From our list of speakers, we then removed honorifics as well as mentions of their district or state within the speaker field (e.g. converting “Ms. Warren of Massachusetts” to “Warren”). Finally, we extract the last token from each politician in both datasets as the surname. Because many politicians have the same surname, we confine our matching of names to DW-NOMINATE scores within states and congressional sessions (e.g. restricting our search Massachusetts politicians in the 115th Congress enables us to correctly match Elizabeth Warren’s speeches to her DW-NOMINATE score instead of Lindsay C. Warren, a North Carolina representative in the 69-76th Congresses.) Finally, to handle misspellings, we use the Python `fuzzywuzzy` package to match misspelled names.[‖] All in all, we were able to match speakers for 85% of speeches in our dataset to DW-NOMINATE scores.

Among those matched speakers with at least 10 speeches relevant to immigration, we calculated their average tone, i.e., their percentage of pro-immigration speeches minus percentage of anti-immigration speeches. We stratified the speakers by party (Democrat and Republican) and time period (pre-1924, 1924-1965, and post-1965), so that we could analyze how the correlation between our tone measure and DW-NOMINATE varied across parties and over time.

[¶] https://voteview.com/data
[‖] https://pypi.org/project/fuzzywuzzy/

Our results show that the first dimension of DW-NOMINATE, which captures the liberal–conservative spectrum, has a negative relationship with our tone measure. That is, more conservative speakers within each party tend to be more anti-immigration. For example, in the post-1965 era, when the relationship is strongest, regressing immigration tone on DW-NOMINATE dimension 1 yields a regression coefficient of -0.68 (s.e. $= 0.08$) for Democrats and -0.90 (s.e. $= 0.06$) for Republicans.[**] The relationship is weaker but still negative from 1924–1965, and weakly positive before 1924, when immigration was not yet a polarizing issue.

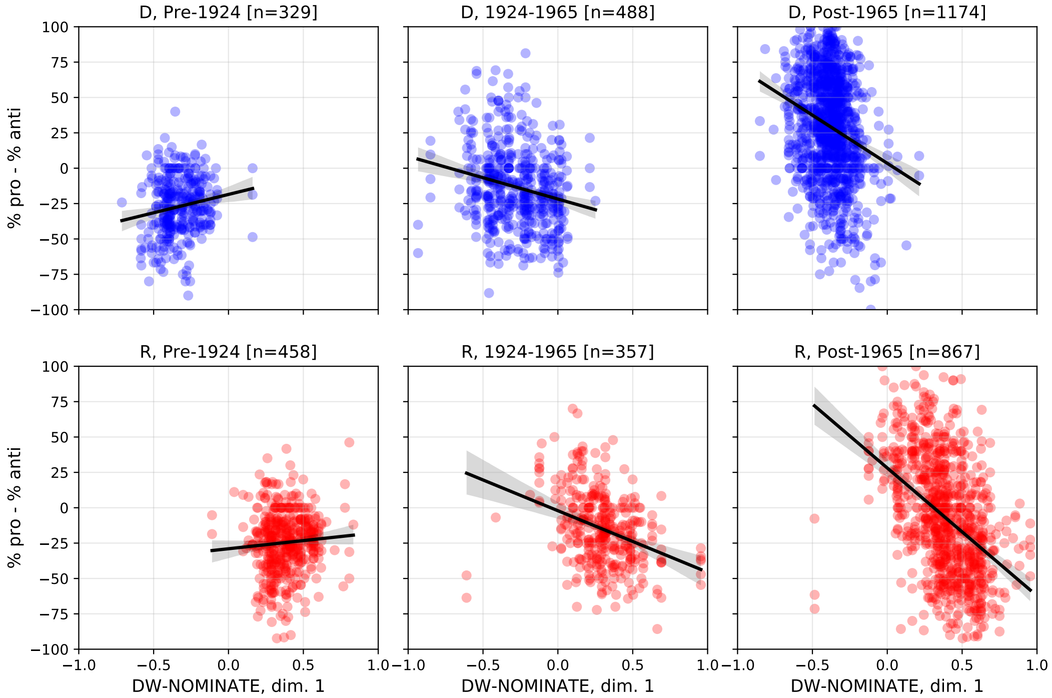

Fig. S16. Association between the mean tone of legislators with at least 10 speeches related to immigration (% pro - % anti) and the first dimension of DW-NOMINATE scores, in three time periods.

We also find that the second dimension of DW-NOMINATE has a negative relationship with our tone measure. The interpretation of the second dimension is more ambiguous; it tends to capture cross-cutting issues that are not already captured by the liberal–conservative spectrum. Like the first dimension, we find that the relationship becomes increasingly negative over time and it is stronger for the Republican party. For example, in the final post-1965 era, the regression coefficients are -0.25 (s.e. $= 0.03$) and -0.39 (s.e. $= 0.04$) for Democrats and Republicans, respectively.

These results demonstrate that our tone measure is not only correlated with political ideology, but also that it can capture variation across individuals within each party. Furthermore, this correlation becomes stronger over time, supporting the argument that immigration has become an increasingly polarized issue.

Association between Tone and Public Opinion

An important question regarding Congressional speech on immigration is to what extent positions are driven by coordinated messaging from party leadership (top-down), as opposed to individual reactions to voter opinions (bottom-up). Although we cannot fully resolve this question here, we briefly investigate the relationship between tone and public opinion over time and across states using data from Gallup.

Public opinion data. To capture public opinion, we used Gallup surveys, which we accessed from the Roper iPoll database. In order to get a standard metric with reliable data over the longest possible timespan, we focused on the question, “In your view, should immigration be kept at its present level, increased or decreased?” Since we wanted to compute immigration attitudes per state, we focused on Gallup surveys for which we had microdata; that is, individual-level responses, where we knew each individual’s response on the immigration question as well as other relevant demographics such as their home state and party affiliation. We found 12 surveys from 2000–2014 for which we had this data.

We found—as in Congressional speeches—that attitudes towards immigrants improved over time among Democrats but not among Republicans. We also found in general that more respondents wanted to keep immigration at its present levels or

[**] Typically our tone measure, as a difference of percentages, falls between -100 and 100, while DW-NOMINATE falls between -1 and 1 (as shown in Figures S16 and S17). However, here we rescaled our tone measure to the range of -1 to 1 when reporting regression coefficients, so that two measures could be on the same scale.

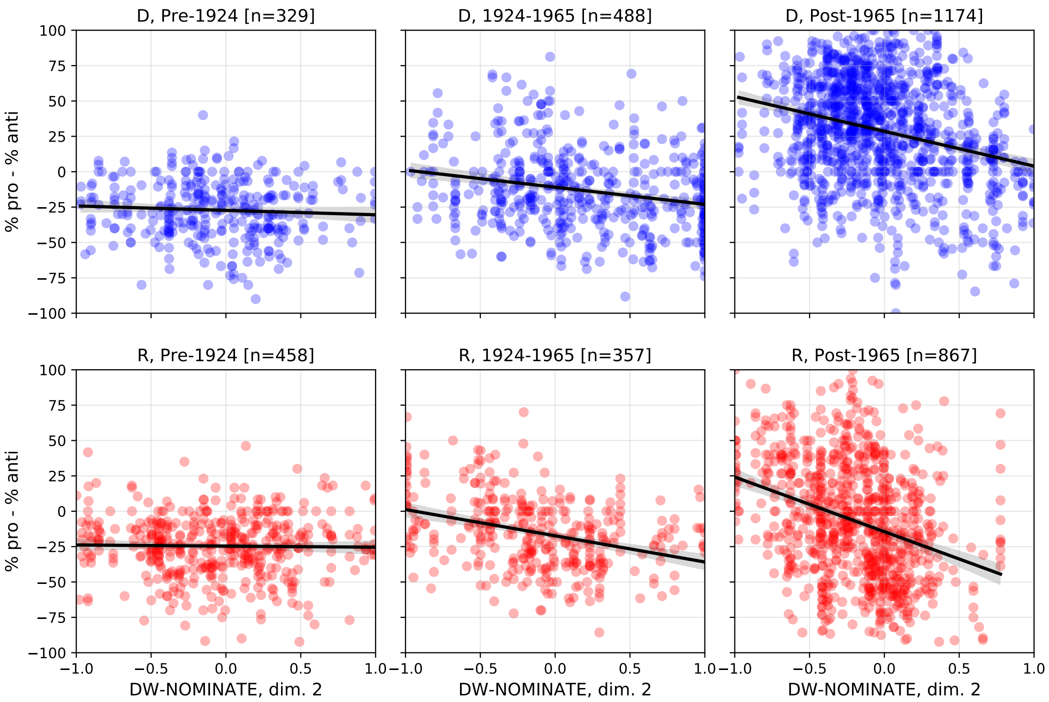

Fig. S17. Association between the mean tone of legislators with at least 10 speeches related to immigration (% pro - % anti) and the second dimension of DW-NOMINATE scores, in three time periods.

decreased, and fewer wanted levels increased. On average over the 12 surveys, 33% of respondents wanted immigration levels kept at present, 16% wanted them increased, and 42% wanted them decreased (see Figure S18).

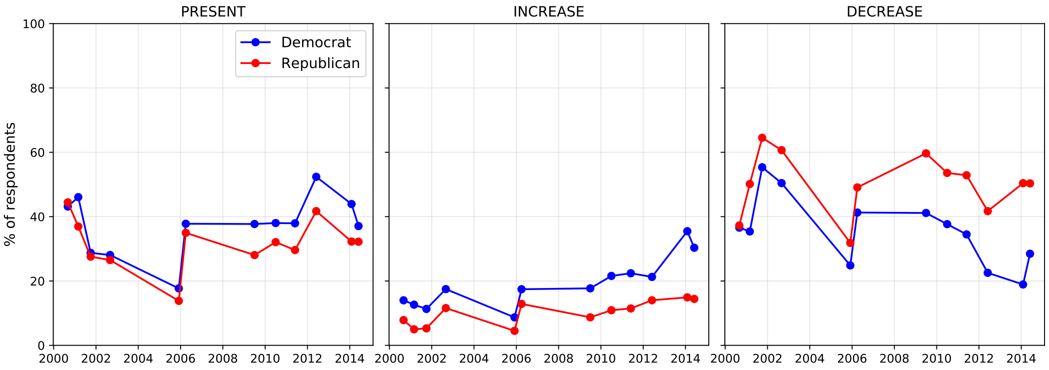

Fig. S18. Percent of respondents in each party who answered that they wanted immigration kept at present levels (left), increased (middle), or decreased (right).

Regression Analysis. We compared Congressional speeches to the public opinions data by aggregating both sources of data to the year-by-state level. In particular, for a given year and state, we started with all of the immigration speeches made in that year by members of Congress from that state, and computed the percentage of those speeches that our classifier labeled as anti-immigration. Similarly, for each year and state, we also took all of the responses from Gallup survey(s) in that year by individuals living in that state, and computed the percentage of responses that wanted immigration decreased.

We then report the relationship between the share of speeches that are anti-immigration and the share of respondents who report wanting immigration decreased in a scatter plot (Figure S19), controlling for year fixed effects. Each observation is weighted by the number of speeches in that state-year cell to address differences in precision across observations. The relationship between these two variables is positive and the coefficient (represented by the slope of the line) is 0.278 (s.e. = 0.078). In other words, within a state, as the local population reports attitudes that are more anti-immigration, political representatives from that state are measured as making more anti-immigration speeches. This correlation does not tell us the direction of the relationship between the local attitudes and political speeches—it could be that politicians are responding to changing attitudes in the electorate, or that local residents are influenced by political elites—but we find the association between these two state-level measures of attitudes toward immigration to be reassuring.

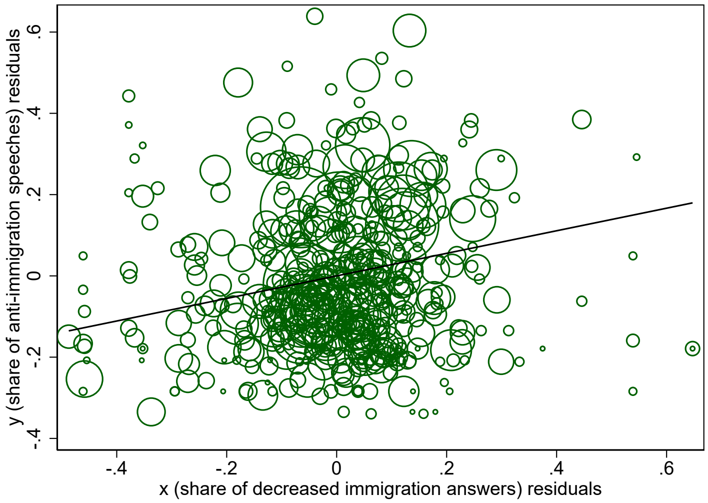

Fig. S19. Scatter plot of the relationship between the share of anti-immigration speeches and share of people wanting immigration to be decreased, after controlling for year fixed effects. Circle size indicates number of people polled at the state-year level.

Influence of C-SPAN and Elections

We conducted a supplementary analysis to test whether the divergence in attitudes toward immigration that we observed between Democrat and Republican members of Congress could be partially explained by the introduction of technology, such as C-SPAN, that allowed voters to watch Congressional speeches. If changes in Congressional speeches were driven by a combination of C-SPAN and desire to appeal to voters, we hypothesized that after the introduction of C-SPAN in 1979, we should see a difference in the tone of immigration speeches when elections were imminent versus further away.

Regression analysis. To analyze this, we focused on the House of Representatives. Representatives hold two-year terms, and elections are held in the November of every even-numbered year. This simplified our analysis, since we could straightforwardly compute the amount of time left until the next election.

In our first regression model, we aggregated immigration speeches to the level of year and party. For each year and party, we kept all of the immigration speeches given in that year by members of the House in that party. We then computed our average tone measure, i.e., the percentage of pro-immigration speeches minus the percentage of anti-immigration speeches. For each party, we regressed average tone on the interaction of whether it was pre-1979 or post-1979 and whether it was an election year (even) or non-election year (odd), with fixed effects for decades. We found for both parties that there was no significant difference in tone between on- and off-election years, either pre-1979 (before C-SPAN) or post-1979 (after C-SPAN).

In our second regression model, we conducted a very similar analysis, but instead of simply indicating whether it was an on- or off-election year, we provided as an independent variable the number of months until the next election. For example, November in an even year mapped to 1, October mapped to 2, and so on, and December to 24. In an odd year, November mapped to 13, October to 14, and so on, and December to 12. Again, we found no significant effect for either party: both pre-1979 and post-1979, the number of months until the next election did not have a significant effect on average tone.

Thus, we did not find evidence that the polarization we observed could be explained by the advent of new technology like C-SPAN and a desire to appeal to voters. In addition, Congressional Representatives seem to not be altering their tone with respect to immigration in election years, which suggests that anti-immigration attitudes are not being driven primarily by electoral cycles.

Countries, Regions, and Human Capital

To demonstrate that Figure 2 in the main paper (which plots tone over time for the three most frequently mentioned nationalities—Italian, Chinese, and Mexican) is representative of broader regional trends, we create an equivalent plot here for the corresponding regions (Europe, Asia, and Latin America) as shown in Figure S20. Specifically, we count mentions of the 45 most prominent countries, in terms of immigration to the U.S. For the sake of this figure, we take Latin America to be the Spanish-speaking countries of Central, South America, and the Caribbean, including Mexico, and include mentions of "Hispanic(s)" and "Latino(s)/Latina(s)" in identifying relevant speeches.

Although the resulting trends in tone are slightly different, with Latin America and Asia being slightly more positive than Mexico and China, and Europe as a whole being slightly more negative than Italy, resulting in smaller gap between lines, the overall pattern is consistent, with the tone in all regions rising mid-century, and mentions of immigration from Latin America remaining more negative than the other regions.

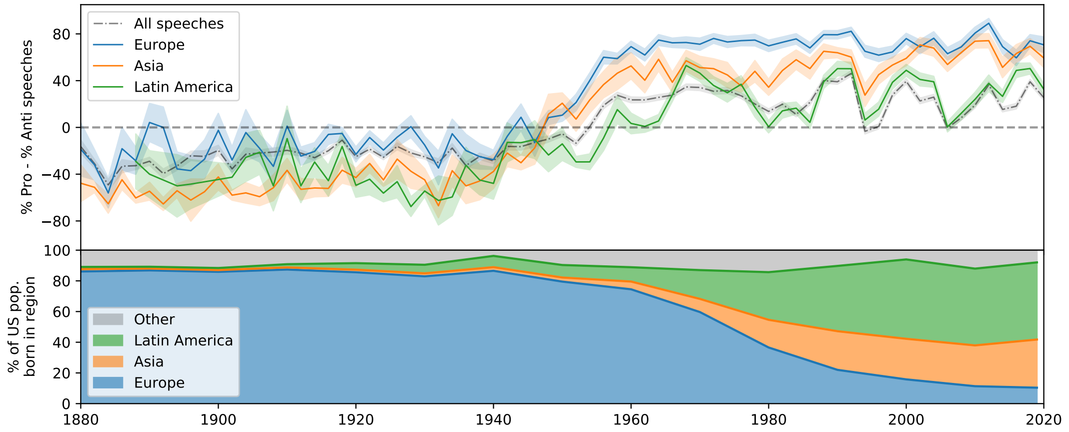

Fig. S20. Average tone of immigration speeches when considering only those that mention immigrants from three broad regions (top), and the percent of the US foreign born population from each of these regions over time (bottom).

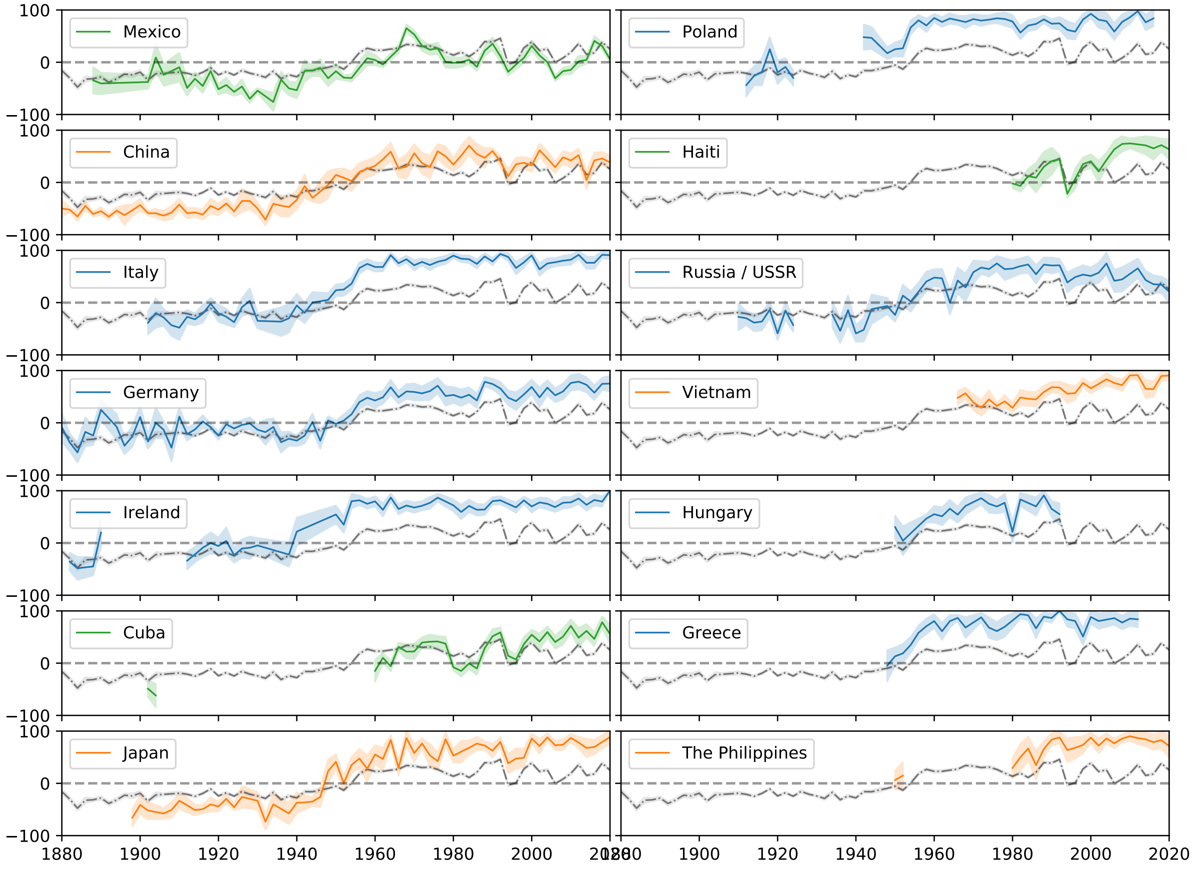

Fig. S21. Difference in frequency of pro minus anti immigration speeches for the fourteen most frequently-mentioned nationalities (excluding English, French, Indian, and Canadian), when considering only those speeches which mention a particular country or nationality. Although it is difficult to generalize from a limited number of countries, we see that most European countries groups are referred to positively by 1960s, most Asian countries by the 1980s, and countries from Central America and the Caribbean remaining negative until the 2000s or later (with Mexico remaining at or below the average tone until the present day).

Figure S21 shows the estimated tone per Congress for each of the fourteen most frequently mentioned nationalities, in order (top to bottom, then left to right), excluding English, French, Indian, and Canadian (as speeches mentioning these terms tend to include content not related to immigrants from the corresponding countries). Each time series shows the overall overall tone (% pro - % anti) for all speeches that include mention of the country or nationality. Only sessions of Congress with at least 20 relevant speeches for that nationality are shown. Bands show uncertainty estimates based on estimated proportion of pro and anti speeches and the number of relevant speeches in that session of Congress. Colors indicate the region of the

country (blue is Europe, orange is Asia, and green is Central and South America and the Caribbean). As can be seen, nearly all European countries had a mean tone that was higher than the overall tone average (for all immigration speeches) by 1960, though less so for Germany and the USSR. By contrast, Asian countries did not achieve this until approximately 1980. Haiti and Cuba only became more positive than the average in the 2000s, and Mexico is still at or below the overall average today. The corresponding mention frequencies are shown in Figure S22.

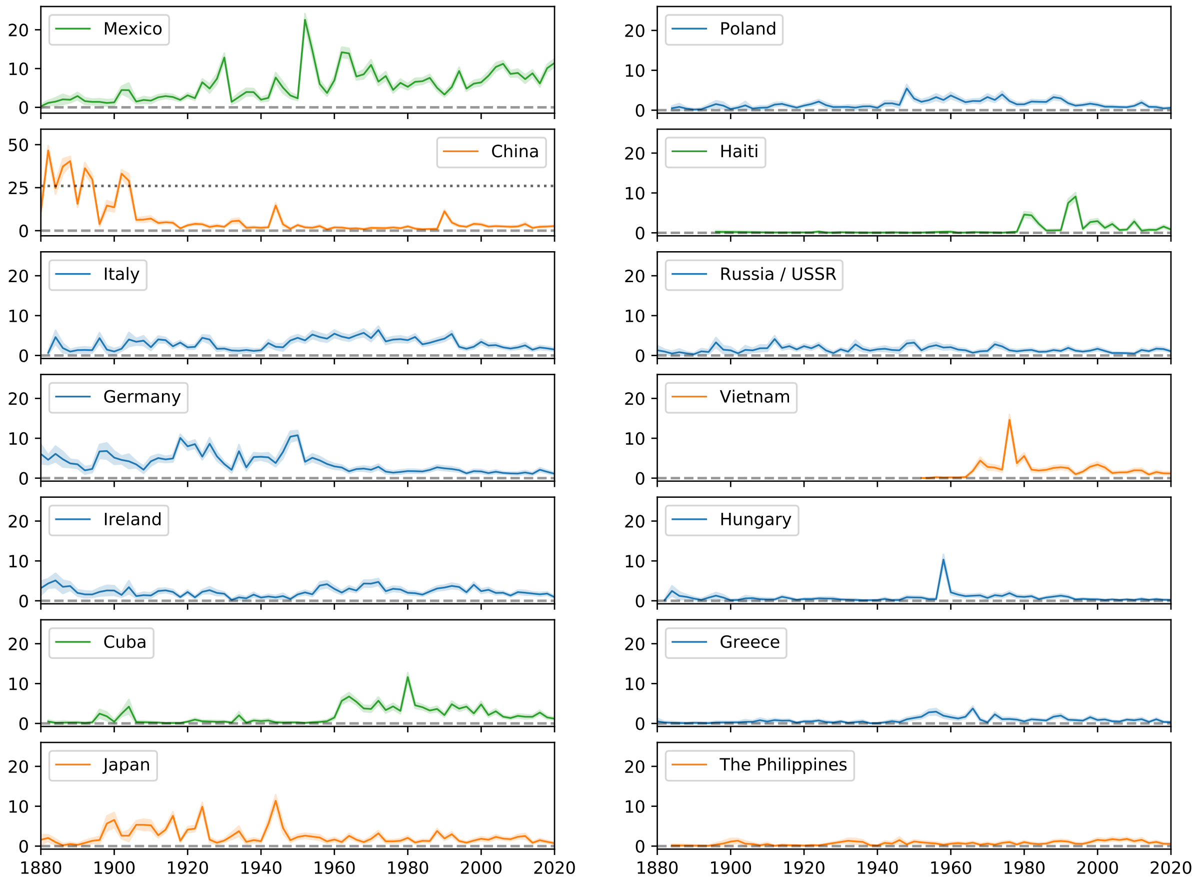

Fig. S22. Mention frequency over time (as a percentage of speeches) for each of the fourteen most frequently-mentioned nationalities within immigration speeches (i.e., number of immigration speeches mentioning a country or nationality as a percentage of the total number of immigration speeches). Color indicates region (Europe, Asia, or Central America and Caribbean). Note that the plot for China (one below the upper left) uses a different scale (with the comparable scale shown as a dotted line), as its maximum mention frequency is twice as high as the country with the next highest maximum frequency (Mexico).

Regression Analysis. In order to check whether these differences in tone can be explained by factors other than nationality, such as human capital or the number of people emigrating from particular places, we include an additional analysis based on micro-data from the U.S. Census, accessed via the IPUMS website.[††] Specifically, we include (a) the share of the population composed of immigrants from these 14 countries of origin, and (b) the socio-economic status of these groups from 1880 to 2020.[‡‡] We use the Duncan Socio-Economic Status Index (SEI) for part (b), a measure that is based on occupational data.

We report results from a regression with 14 countries of origin-by-decade observations. In each case, we calculate the share of speeches about that country-of-origin in that decade that are pro-immigration or anti-immigration. Our dependent variable is % pro - % anti, as in our main results (e.g., Figure 1). As explanatory variables, we include: (a) country-of-origin fixed effects, (b) decade fixed effects, and (c) two additional attributes of the country-of-origin by decade (= share of the population and socio-economic index). The country-of-origin fixed effects produce sensible results: speeches about immigrants from Europe are the most positive, and speeches about immigrants from the Americas are the most negative. Speeches about immigrants grow more positive over time (see Table S6).

We find that, controlling for these country-of-origin and decade fixed effects, groups that are becoming more numerous (% of the population is rising) are spoken about more negatively, as are groups that are becoming higher in socio-economic status (SEI is rising). The first pattern is quite sensible: we would expect that groups that are growing in size become more salient and perhaps more worrying to the electorate. The second pattern is an interesting new finding and would need to be probed

[††] https://www.ipums.org/

[‡‡] Note that there is no Census data available for 1890.

more in future work. One explanation could be that groups are spoken about very positively when they are refugees and perceived to be in need of help (e.g., Cubans, Vietnamese) and this positive speech diminishes as the groups become perceived to be made up of more "economic" migrants over time. Exploring these hypotheses in greater detail is beyond the scope of this paper, but the patterns that emerge are interesting and worthy of further study.

	% pro - % anti
% foreign population	-0.007**
	(0.003)
Average Duncan Socioeconomic Index	-0.012*
	(0.006)
**Country**	
Cuba	-0.040
	(0.07)
Germany	0.250***
	(0.06)
Greece	0.263***
	(0.05)
Haiti	-0.218**
	(0.11)
Hungary	0.109
	(0.09)
Ireland	0.321***
	(0.05)
Italy	0.241***
	(0.08)
Japan	0.123*
	(0.07)
Mexico	-0.270**
	(0.11)
Philippines	0.221***
	(0.07)
Poland	0.272***
	(0.07)
Russia	0.073
	(0.06)
Vietnam	0.199**
	(0.09)
Constant	-0.360***
	(0.12)
Decade fixed effects	Yes
Country fixed effects	Yes
R^2	0.89
N	157

Table S6. Data is at the country and decade level. Each column shows the difference between the share of pro- and anti-immigration speeches regressed on the share of the foreign population and the average Duncan socioeconomic index by decade and country. The omitted country category is China. Standard errors are in parentheses. * $p < 0.1$ ** $p < 0.05$, *** $p < 0.01$.

Immigration Topics

Figure S23 shows a set of 40 topics discovered using Latent Dirichlet Allocation (10), plotted in terms of the mean document proportions over time. As can be seen, some are procedural (e.g., "act, section, amendment"), some group together nationalities (e.g., "chinese, treaty, china, government, japanese"), some reflect aspects of the immigration debate that are relatively focus in time (e.g., "education, school, students"), and some represent enduring issues (e.g., "tax, percent, budget"). Most however, are relatively localized in time. Because of this, we choose to make use of semi-automatically constructed frames (tagged lexicons) for measuring the prevalence of immigration frames across the entire time period (see below).

Curating Immigration Frames

In order to deepen our analysis of the language used in relation to immigrants, we constructed a set of fourteen immigration "frames", i.e., thematic groups of words used in association with immigrants, and measured their prevalence across parties, across ethnic groups, and over time.

In this section, we discuss how we chose these fourteen frames to focus on, and how we constructed word lists for each frame. Our approach consisted of three steps: (1) we applied computational methods to uncover all words that were used

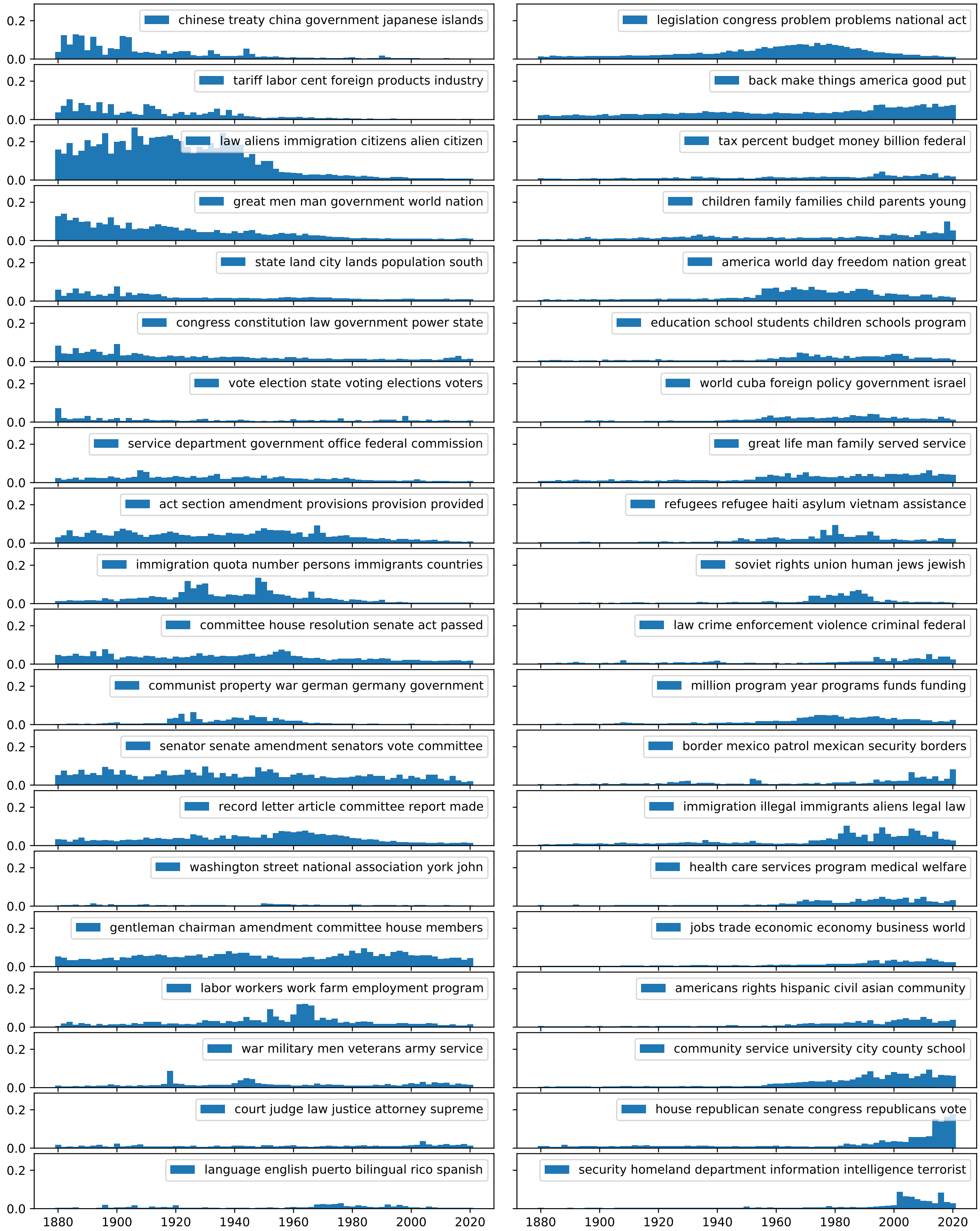

Fig. S23. Forty topics discovered using LDA, plotted in terms of average document proportions over time.

significantly more in reference to immigrants as opposed to generic people mentions (e.g, "man", "woman", etc.); (2) based on a combination of automatic word clustering methods and hypotheses from the literature on immigration, we identified fourteen relevant frames; (3) all authors on this paper manually annotated the immigrant-associated words to label each one with the frame(s) that they belonged to.

Identifying immigrant-associated words. In the first step, our goal was to automatically identify all words used in association with immigrants, i.e., words that were used significantly more frequently to modify immigrant terms than general person terms. First, we constructed two groups of "anchor" terms, one of immigrant terms (e.g., "immigrant", "emigrant") and one of generic terms related to people (e.g., "person", "man", "woman"), which also included the immigrant terms; we provide a full list of these anchor terms in Table S7.

Table S7. Anchor terms used to identify mentions of immigrants and generic mentions of people

Group	Terms
Immigrants	immigrant, emigrant, foreigner, alien, refugee
People	man, woman, male, female, boy, girl, brother, sister, husband, wife, father, mother, son, daughter, parent, child, relative, adult, person, friend, resident, neighbor, worker, non-resident, inhabitant, farmer, laborer, merchant, manufacturer, lawyer, supervisor, slave, servant, consumer, owner, assistant, pauper, applicant, passenger, teacher, employer, inspector, student, employee, workman, writer, doctor, professor, immigrant, foreigner, pioneer, emigrant, alien, refugee, settler, widow, sailor, engineer, surgeon, miner, trader, cook, poet, earner, traveler, peasant, tenant

To identify modifying words, we applied part-of-speech and dependency parsing to all of the sentences in the Congressional speeches. For a given anchor term, we collected all adjectives, verbs, and nouns that appeared in the speeches with certain dependency relations to the anchor term. For example, we collected all adjectives that were adjectival modifiers of the anchor term, such as "illegal" in "illegal immigrants". In Table S8, we list and provide examples of the dependency relations we included for adjectives, verbs, and nouns. Using this approach, we collected two corpora, C_i and C_p, that consisted of all of the words that appeared in one of these dependency relations relative to an immigrant anchor term or a person anchor term, respectively. Note that C_i is a subset of C_p because the immigrant terms were a subset of the person terms.

Table S8. Dependency relations used in identifying referring terms.

Part-of-speech	Dependency path to anchor	Example
Adjective	**XX** –amod→ ANC	**poor** immigrants
	**XX** –amod→ YY ←amod– ANC	**insane** alien paupers
Verb (subject)	ANC –nsubj→ **XX**	immigrants **come**
	**XX** –relcl→ ANC	immigrants who are **pouring**
	**XX** –acl→ ANC	immigrants **arriving**
Verb (object)	ANC –dobj→ **XX**	**shut** out immigrants
Noun	ANC –pobj→ YY –prep→ **XX**	**education** of immigrants
	ANC –amod→ **XX**	alien **flags**
	ANC –compound→ **XX**	emigrant **passengers**

To identify terms that were significantly associated with immigrants, we compared the relative frequency of words in C_i versus C_p. Formally, for a given term w (defined by a lemma and a part-of-speech), we computed its relative "background" frequency $f_p(w) = c_p(w)/N_p$, where $c_p(w)$ is the count of the term in the person corpus C_p and N_p is the total number of words in C_p. These terms are defined analogously for the immigrant corpus as f_i, c_i, and N_i. Then, we computed the probability of observing $c_i(w)$, the count of the word in the immigrant corpus, as a random sample from Binomial$(N_i, f_p(w))$. If this probability p_w was low, this would indicate that the word was appearing significantly more frequently than we would expect if $f_i(w) > f_p(w)$, or significantly less frequently than we would expect if $f_i(w) < f_p(w)$, in relation to immigrants as opposed to people in general.

We applied these methods to two representative periods of Congressional speeches, 1880-1929 (sessions 46-70) and 1965-2020 (sessions 89-116). For each period, we identified immigrant associations as the words that met the following criteria: (1) its relative frequency $f_i(w)$ in the immigrant corpus was higher than its relative frequency $f_p(w)$ in the person corpus, (2) its probability p_w was below 0.1, (3) it appeared at least 10 times in C_i and C_p. With these criteria, we were left with 70 adjectives, 138 verbs, and 160 nouns from the first period, and 156 adjectives, 289 verbs, and 331 nouns from the second period, with 898 unique words (lemma and part-of-speech) in total. In Figure S24, we also visualize a subset of these words—the "strongest" associations—for which $f_i(w)$ was at least 5 times as large as $f_p(w)$.

Identifying immigrant frames. In our second step, our goal was to construct frames from among the immigrant-associated words. We first approached this automatically, using a combination of word embedding and clustering techniques. In order to learn word embeddings that were specific to the context of the Congressional speeches, we trained our own word embeddings on the Congressional speeches using word2vec (11). As input to the word2vec model, we provided all of the immigration speeches identified above.[§§] We fit 100 dimensional word embeddings using the gensim python package with the default word2vec parameter settings (window size of 5, CBOW algorithm, etc.) using `gensim` (https://radimrehurek.com/gensim/).

[§§]The data used for this step (prior to manually classifying the framing terms) were actually based on a slightly earlier version of our classifiers, applied to a slightly different representation of the data, though the resulting set of terms is very similar.

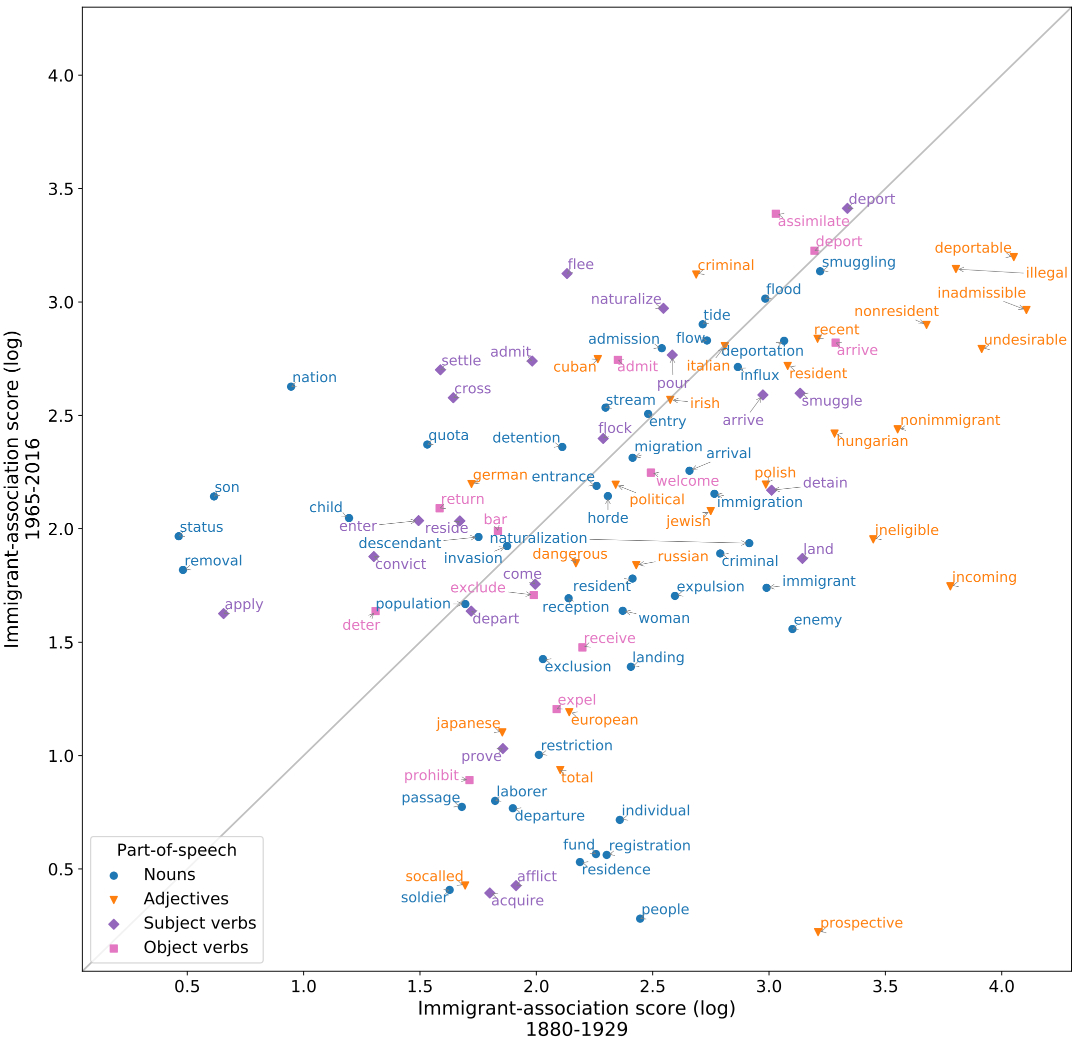

Fig. S24. Most immigrant-associated words from two contrasting periods. The score is the log of the frequency of the word in the immigrant corpus, $f_i(w)$, divided by the frequency of the word in the person corpus, $f_p(w)$, as defined in Curating Immigration Frames, such that higher scores are terms that are more associated with immigrants. The x-axis shows the score in the earlier time period, and the y-axis the same score in the later period. From the 898 words that we kept (see Table S8 for filtering criteria), we plot the words whose scores are at least 5 for one or both periods. The grey line indicates $y = x$; falling above the line (e.g., the verb "flee") means that the word was more associated with immigrants in the later period, falling below the line (e.g., the adjective "undesirable") means that the word was more associated with immigrants in the earlier period.

Then, we gathered the word embeddings for each of the immigrant-associated words output in the previous step. To identify potential themes among these words, we ran k-means clustering on their word embeddings, with a range of possible cluster numbers ($k = 10, 20, 30, 40$). Since k-means can get stuck at local minima (i.e., fail to find the globally optimal clustering), for each k, we ran the algorithm 50 times with random initialization, and kept the results that achieved the lowest within-cluster sum of squared errors. Through a qualitative review of the resulting word clusters, we found that using $k = 30$ struck the right balance between finding detailed themes and producing decently-sized clusters with more than a handful of words. Furthermore, we found that this approach was able to identify many coherent word groups, such as clusters related to numbers, masses and water metaphors, family, exclusion, crime, legality, funding, employment, and nationalities.

However, the automatic clusters were imperfect; for example, there was still one very large cluster of over 130 words that were "left over" without any obvious theme, and there was an overemphasis in the automatic clusters on part-of-speech tags, such that several clusters were entirely verbs or adjectives (this was due to how word embeddings capture context in sentences). So, we decided to manually construct our own set of frames, but we used the themes surfaced by the automatic clustering to inform the frames that we chose. We also allowed our choices to be informed by themes discussed in the literature on immigration, and free-text comments provided by the annotators. Ultimately, we settled on fourteen frames, many of which overlapped with the automatic clusters: *Contributions*, *Victims*, *Family*, *Culture*, *Deficient*, *Crime*, *Threat*, *Economic*, *Labor*, *Legality*, *Quantity*, *Flood/Tide (Water)*, *Exclusion*, and *Migration*.

Grouping words into frames. In our last step, our goal was to annotate each of the immigrant-associated terms from step 1 with the frame(s) from step 2, so that ultimately, each frame would correspond to a set of immigrant-associated terms. For each term, the annotator was required to choose at least one frame for the word (with a 15th option for "Other"), and could optionally choose a second frame as well if they felt that the term belonged to multiple frames. When labeling a word, the annotator was given the term's lemma and part-of-speech (e.g., "flood" (verb)), and used their judgment about the word and its probable contexts, along with knowledge of the discourse around immigration, to make their determination.

Each of the 9 researchers on our team annotated a random sample of half or more of the 898 words. As a result, the large majority of words had at least 5 annotations, and every word had at least 3. Then, for each word and frame, we added the word to the frame's word set if more than half of its annotators agreed that this word belonged to that frame. This meant that a word could end up in multiple frames; for example, the noun "terrorist" ended up in both the *Crime* and *Threat* frames. Using this word, 403 of the immigrant-associated words were added to at least one of the fourteen frames. *Exclusion* (n=49) was the largest resulting frame and *Deficient* (n=14) was the smallest.

Many of the frames that emerge from this process show a clear overlap with previously attested aspects of immigration rhetoric, and capture a mix of positive (e.g. *Contributions*), neutral (*Economics*, *Migration*), and negative (e.g., *Threats*), as well as the frequently deployed metaphors of a "flood" or "tide" of immigrants (e.g., "flood", "flow", "drain", etc.). The full list of words, separated into their respective frames, are listed in Table S9.

Party and Group Comparison with Expanded Frames

Because our findings could be sensitive to the specific set of terms included in each frame, we provide two types of validity checks. First, all relevant figures provide the resulting values obtained (minimum and maximum) if we leave out each word in turn, demonstrating that, in almost all cases, no one word is solely responsible for the associations.

Second, to ensure that we have not missed any important terms in constructing these frames, we again use word vectors to find additional terms related to each frame, and redo our analysis. To find these additional terms, we again use word2vec (11) to train word vectors, this time using the immigration speeches, along with a random 20% of the rest of the corpus. For each frame, we identify up to 20 additional terms to add to that frame.

To get these related terms, we find the lemmas that are most similar to the average of the vectors for the manually-curated terms for a given frame (ignoring parts of speech). We then add the most similar terms (keeping all frequently occurring parts of speech for that term) if a) a term is among the most similar terms for a given frame; b) it is more similar to that frame than any other frame; and c) it is not already part of any of the manually constructed frames.

The full list of additional terms is given in Table S10. Figures S25 and S26 reproduce Figures 4 and 5 in the main paper after expanding the frame lexicons to include these automatically identified terms. As can be seen, using the expanded lexicons makes no meaningful difference to the trends we have observed.

Finally, we include an alternative to Figure 4 in the main paper, where we compare modern mentions of European immigrants to mentions of immigrants from all Latin American countries (which we define for the sake of this figure as Spanish-speaking countries of Central America, South America, and the Caribbean, plus Mexico), including mentions of Hispanic(s) and Latino(s)/Latina(s), rather than just immigrants from Mexico.

As can be seen in Figure S27, the results are similar, with *Family*, and *Victims* still being more associated with Europeans, and *Labor*, *Exclusion*, *Crime*, and *Legality* still being associated more with non-Europeans. However, with this comparison, we find no significant difference in the use of dehumanizing language, which further reinforces that Mexico holds a unique position within modern immigration discourse.

Quantifying Usage of Dehumanizing Metaphors

Methodological details. To identify more subtle dehumanizing metaphors, beyond the explicitly used framing of a "flood" or a "tide" of immigrants, we focus on a set of metaphors previously discussed in the literature on immigration, and make use of

Table S9. Curated word lists for frames. Letters in parentheses indicate part of speech (n = noun, v = verb, a = adjective). Note that hyphens between words were dropped in preprocessing the speeches, hence the appearance of terms like "selfsufficiency (n)".

Frame	Curated Terms
Contributions	ability (n), attract (v), build (v), contribute (v), contribution (n), desirable (a), enlist (v), enrich (v), friendly (a), gain (v), genuine (a), hardworking (a), hero (n), highskilled (a), industrious (a), lawabide (v), loyal (a), patent (n), qualified (a), selfsufficiency (n), sturdy (a), veteran (n), worthy (a)
Crime	absconder (n), apprehend (v), arrest (n), arrest (v), bootlegger (n), capture (v), convict (v), criminal (a), criminal (n), criminalize (v), detain (v), detainee (n), felon (n), fugitive (a), gang (n), illegal (a), imprison (v), imprisonment (n), incarcerate (v), incarceration (n), inmate (n), jail (v), noncriminal (a), offense (n), parole (n), parole (v), prosecute (v), prosecution (n), smuggle (v), smuggler (n), smuggling (n), steal (v), terrorist (a), terrorist (n), unlawful (a), violent (a)
Culture	americanization (n), assimilate (v), assimilation (n), background (n), christian (a), citizenship (v), culture (n), diverse (a), diversity (n), ethnic (a), flag (n), foreigner (n), heritage (n), integrate (v), integration (n), language (n), nation (n), race (n), society (n), tradition (n)
Deficient	aidsinfected (a), cheap (a), diseased (a), drunk (a), ignorant (a), illiteracy (n), illiterate (a), insane (a), insane (n), objectionable (a), slacker (n), undesirable (a), unfit (a), unskilled (a)
Economic	agricultural (a), allocation (n), bank (n), budget (n), buy (v), cash (n), cent (n), coin (v), compete (v), competition (n), corporation (n), cost (v), economic (a), finance (v), fund (n), funding (n), investor (n), owner (n), ownership (n), pay (v), property (n), purchase (v), reimbursement (n), sale (n), specie (n), tax (n), tax (v), taxpaye (v), taxpayer (n), workforce (n)
Exclusion	admissibility (n), allow (v), ban (n), ban (v), bar (n), bar (v), cap (n), ceiling (n), criterion (n), debar (v), deny (v), deport (v), deportable (a), deportation (n), deported (a), detain (v), deter (v), eligibility (n), excludable (a), exclude (v), exclusion (n), expel (v), expulsion (n), forbid (v), inadmissible (a), inspect (v), interdict (v), interdiction (n), limit (n), limit (v), limitation (n), overstay (v), prevent (v), prohibit (v), prohibition (n), quota (n), reject (v), removable (a), removal (n), requirement (n), restrict (v), restriction (n), screen (v), screening (n), shut (v), stop (v), unauthorized (a), vet (v), vetting (n)
Family	ancestor (n), boy (n), child (n), daughter (n), descendant (n), descendent (n), family (n), familybased (a), familysponsored (a), generation (n), girl (n), grandchild (n), granddaughter (n), grandparent (n), grandson (n), household (n), husband (n), kid (n), marriage (n), marry (v), neighbor (n), neighborhood (n), orphan (n), parent (n), relative (a), relative (n), son (n), spouse (n), wife (n)
Flood/Tide	absorb (v), absorption (n), drain (v), fill (v), flood (n), flood (v), flow (n), flow (v), inflow (n), influx (n), outflow (n), pour (v), spill (v), stream (n), stream (v), surge (n), tide (n), trickle (n), wave (n)
Labor	agricultural (a), compete (v), competition (n), crewman (n), employ (v), employee (n), employer (n), employment (n), employmentbased (a), farmworker (n), hire (v), hiring (n), laborer (n), miner (n), nurse (n), tailor (n), unskilled (a), worker (n), workforce (n)
Legality	adjudication (n), amnesty (n), applicant (n), application (n), authorization (n), authorize (v), citizenship (n), citizenship (v), eligibility (n), eligible (a), exemption (n), familysponsored (a), hearing (n), identification (n), illegal (a), inadmissible (a), ineligible (a), law (n), lawful (a), legal (a), legalization (n), legalize (v), legalized (a), legitimate (a), license (n), naturalization (n), naturalize (v), overstay (v), permanent (a), prosecute (v), qualify (v), registered (a), registration (n), sponsor (n), sponsor (v), status (n), unauthorized (a), undocumented (a), unlawful (a), unnaturalized (a), verification (n), visa (n)
Migration	arrival (n), arrive (v), boatload (n), come (v), coming (n), convoy (n), cross (v), depart (v), departure (n), depot (n), destination (n), displacement (n), emigrate (v), enter (v), entrance (n), entry (n), exodus (n), flight (n), immigrate (v), incoming (a), land (v), landing (n), leave (v), migrate (v), migration (n), move (v), movement (n), passage (n), passenger (n), port (n), reenter (v), relocate (v), relocation (n), resettle (v), resettlement (n), resettling (n), return (n), return (v), route (n), seaman (n), settle (v), settlement (n), ship (v), shipload (n), station (n), transport (v), travel (v)
Quantity	additional (a), boatload (n), bulk (n), bunch (n), cap (n), count (n), count (v), counting (n), crowd (n), entire (a), estimate (n), estimate (v), estimated (a), flock (v), fraction (n), horde (n), increase (v), majority (n), many (a), mass (n), masse (n), million (n), more (a), most (a), much (a), number (n), number (v), percentage (n), proportion (n), shipload (n), statistic (n), sum (n), thousand (n), total (a), total (n), twothird (n)
Threats	aidsinfected (a), anarchist (n), apprehension (n), attack (n), attack (v), belligerent (n), blame (v), combatant (n), danger (n), dangerous (a), diseased (a), enemy (n), gang (n), horde (n), infect (v), invasion (n), overrun (n), problem (n), terrorist (a), terrorist (n), threaten (v), violent (a)
Victims	acceptance (n), afflict (v), aid (n), aid (v), amnesty (n), assist (v), assistance (n), assisted (a), asylum (n), battered (a), compassion (n), deserve (v), desperate (a), escape (v), exploit (v), feed (v), flee (v), help (v), helpless (a), hope (v), hopeful (a), humanitarian (a), impoverished (a), languish (v), orphan (n), penniless (a), persecute (v), persecution (n), plight (n), prejudice (n), refuge (n), refugee (n), relief (n), rescue (v), reward (v), sanctuary (n), shelter (v), struggle (n), suffering (n), survivor (n), tragedy (n), unfortunate (a), victim (n), welcome (v)

Table S10. Terms added to each of the frames using automated expansion as a validity check.

Frame	Automatic Expansion Terms
Contributions	energetic (a), enterprising (a), enterprising (n), entrepreneurial (a), intelligent (a), intelligent (n), motivate (n), motivate (v), patriotic (a), patriotic (n), productive (a), productive (n), productive (v), resourceful (a), selfreliant (a), selfrespecting (n), talented (a), thrifty (a), thrifty (n), welleducated (a)
Crime	adultery (n), apprehending (a), felonious (a), felony (n), habitual (a), indictable (a), informer (n), kidnap (v), kidnaping (n), kidnapping (n), larceny (n), manslaughter (n), murder (n), murder (v), murderer (n), offender (n), perjury (n), perpetrator (n), rapist (n), trumpedup (a)
Culture	amalgamation (n), americanism (n), anglosaxon (n), caste (n), caucasian (a), civilization (n), cultural (a), cultural (n), folklore (n), individuality (n), lineage (n), linguistic (a), mosaic (a), mosaic (n), multicultural (a), multiethnic (a), nationhood (n), pluralism (n), richness (n), tapestry (n)
Deficient	addicted (a), degraded (a), depraved (a), idiot (n), idiotic (a), illegitimate (a), immoral (a), infected (a), institutionalized (a), lazy (a), lunatic (a), lunatic (n), obese (a), overweight (a), pauper (n), pauperism (n), unclean (a), uneducated (a), unemployable (a), unwanted (a)
Economic	capitalization (n), depreciation (n), financing (n), fnma (n), investment (n), loan (n), loan (v), loans (n), mortgage (n), mortgage (v), passthrough (n), payment (n), payments (n), premium (a), premium (n), sales (n), savings (n), subsidy (n), taxfree (n), taxfree (v)
Exclusion	apply (n), apply (v), banning (n), circumvent (v), discourage (n), discourage (v), disqualify (v), inhibit (v), nullify (v), penalize (v), permit (n), permit (v), preclude (n), preclude (v), prohibiting (n), proscribe (v), remove (v), restricted (a), revoke (v), waive (v)
Family	aunt (n), bride (n), brother (n), cousin (n), daughterinlaw (n), father (n), father (v), grandfather (n), grandfather (v), grandmother (n), mother (n), mother (v), motherinlaw (n), nephew (n), niece (n), parents (n), sibling (n), soninlaw (n), stepfather (n), stepmother (n)
Flood/Tide	avalanche (n), deluge (n), deluge (v), drift (n), drift (v), flooding (n), hemorrhage (n), hemorrhage (v), infusion (n), inundate (v), inundation (n), melt (n), melt (v), overflow (n), overflow (v), seepage (n), swarm (n), swarm (v), tidal (a), torrent (n)
Labor	apprentice (n), artisan (n), bricklayer (n), electrician (n), employers (n), helper (n), journeyman (n), labor (n), labor (v), laborers (n), lowwage (n), mechanic (a), mechanic (n), parttime (n), seasonal (a), skilled (a), wages (n), workers (n), workman (n), workmen (n)
Legality	admissible (a), admission (n), certificate (n), certificate (v), certification (n), conditional (a), disqualification (n), ineligibility (n), noncitizen (n), nonquota (n), nonresident (a), nonresident (n), probationary (a), qualifying (n), readmission (n), registry (n), residence (n), residency (n), revocation (n), vis (n)
Migration	admittance (n), checkpoint (n), commute (n), commute (v), crossing (n), embarkation (n), emigrant (n), entering (n), evacuate (v), ingress (n), landed (a), locate (v), overland (n), repatriation (n), sail (n), sail (v), sailing (n), trek (n), unload (v), voyage (n)
Quantity	aggregate (a), aggregate (n), aggregate (v), oneeighth (a), oneeighth (n), onefifth (a), onefifth (n), onefourth (a), onefourth (n), onehalf (a), onehalf (n), onehalf (v), onequarter (n), onesixth (n), onetenth (a), onetenth (n), onethird (a), onethird (n), onethird (v), percent (n), upwards (a)
Threats	aggressor (n), alqaida (n), cowardly (a), deadly (a), hostile (a), hostile (n), infiltration (n), insidious (a), isis (n), jihadist (n), menace (n), menace (v), murderous (a), onslaught (n), sabotage (n), sabotage (v), terror (n), terrorism (n), terrorize (v), threat (n)
Victims	desperation (n), destitute (a), destitute (n), destitution (n), downtrodden (a), downtrodden (n), hapless (a), impoverishment (n), misery (n), oppressed (a), oppression (n), povertystricken (a), povertystricken (v), privation (n), starving (n), succor (n), torment (n), torment (v), underprivileged (a), wartorn (a)

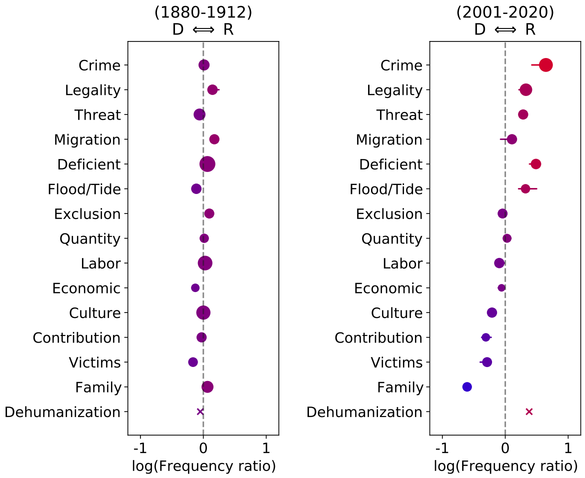

Fig. S25. Comparison of usage of frames by parties (reproducing the analysis in Figure 3 in main paper), when using the expanded frame lexicons combining words from Tables S9 and S10.

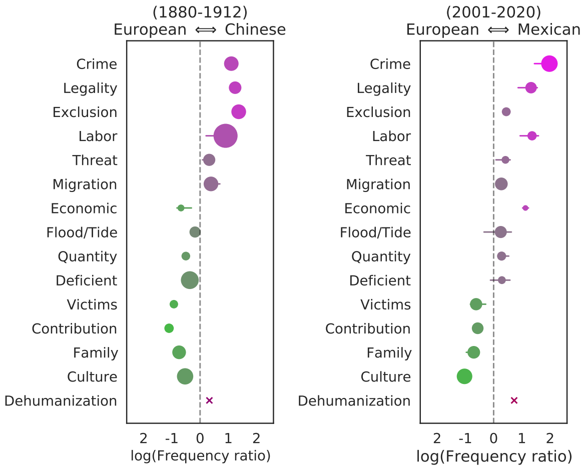

Fig. S26. Comparison of usage of frames based on groups mentioned (reproducing the analysis in Figure 4 in main paper), when using the expanded frame lexicons combining words from Tables S9 and S10.

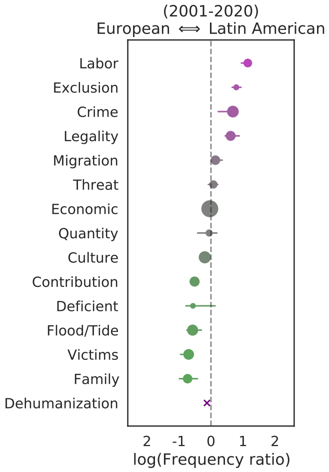

Fig. S27. Comparison of usage of frames based on groups mentioned (reproducing the analysis in Figure 4 in main paper), when comparing mentions of immigrants from Europe to immigrants from Mexico plus Spanish-speaking countries in Central America, South America, and the Caribbean, rather than just Mexico.

contextual embedding models to measure the extent to which mentions of immigrants "sound like" each of several metaphorical category.

The basic idea of this method is illustrated in Figure S28. Contextual embedding models, such as BERT (12), are trained to predict the identity of randomly masked words based on the surrounding context. Here, we repurpose the model by intentionally masking entire mentions of immigrants (which could be, for example "aliens" or "Mexican nationals", etc.), and computing the probability—according to the model—that each word in its vocabulary would serve as a replacement for the mask. By adding up the probabilities for a set of words which we have previously identified as being representative of particular categories, we get an estimate of how much a particular mention suggests the corresponding metaphor. In Figure S28, for example, the reference to "dumping" something "into this country" suggests that words in the *Cargo* category would be likely replacements.

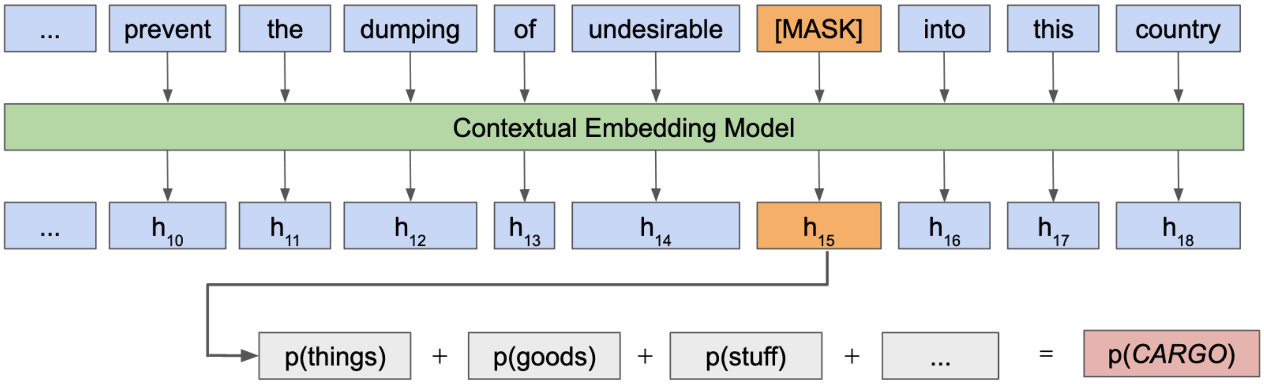

Fig. S28. Schematic depiction of our method for measuring metaphorical language, here showing an example which activates the *Cargo* category.

Target terms. Because BERT has a relatively constrained vocabulary (of approximately 30,000 tokens, representing both whole words and word pieces), we can easily identify the tokens in the vocabulary that are appropriate for each category. Beginning with an initial set, we use static embeddings to look for semantically similar terms, and then limit the list to those that are in the BERT vocabulary as whole words. The full set of terms we use as targets for each of the metaphorical categories are given in Table S11.

The table also includes a set of random control terms. To choose these, we counted the occurrence of all words that occur as nouns in the Congressional Record (after parsing it with spaCy), and restricted the possible set to those words that occur at least 1000 times, and those that exist as whole words in the BERT vocabulary. We then selected a random set of 50, excluding all terms that had previously been used in identifying immigrants, nationalities, or other metaphorical categories. Finally, we noted that the resulting random list included the word "humans". Since this term would account for most of the probability mass for the *Random* category, we exclude this term, although leaving it in leads to similar results.

Trends over time. Applying these to all mentions of immigrants (see Sections Methods and Materials in main paper), we can compute the average probability for each category across mentions by party in each session of Congress. The raw log probabilities for all metaphorical categories decline over time. However, in order to correct for unrelated factors which might explain this decline (e.g., due to older data being less similar to the data that BERT was trained on, or factors related to the Congressional Record itself), we also make use of a set of random control terms to correct for this, as described above.

In more detail, to estimate the prevalence of contexts which cue these metaphors over time, we compute the average log probability assigned to all terms in each category at each point in time (across contexts), and divide by the number of terms in the metaphorical category. We then repeat this for the terms in the *Random* category. Finally, we plot the log of the ratio between these two, which is equivalent to the log of the first minus the log of the *Random* category, as shown in Figure S29.

More formally, for a set of N contexts (mentions of immigrants), we compute the relative log probability for metaphor m as,

$$\text{relative log prob} = \log\left(\frac{\sum_{i=1}^{N}\sum_{w\in W_m} p(w \mid c_i)}{N\cdot|W_m|}\right) - \log\left(\frac{\sum_{i=1}^{N}\sum_{w\in W_r} p(w \mid c_i)}{N\cdot|W_r|}\right),$$

where c_i is the ith context, W_m is the set of words associated with metaphor m, $|W_m|$ is the number of terms in that category, W_r is the set of terms in the *Random* category, and $p(w \mid c_i)$ is the probability assigned to word w in context c_i with the masked mention.

After correcting for changes in random terms, we see in Figure S29 that in fact there is no significant increase or decrease in dehumanizing metaphors over time. In addition, we can see that the *Animal* and *Cargo* words are the most prominent. By contrast, the *Vermin* terms are actually less likely than random terms, on average, though we still see that they are significantly more likely in speeches by Republicans than Democrats in the past two decades.

Table S11. Target words used for each of our metaphorical categories

Metaphor	Terms in BERT vocabulary
Animals	animal, animals, beast, beasts, brute, cattle, cow, cows, dog, dogs, herd, herds, hog, horse, horses, livestock, pig, pigs, sheep
Cargo	thing, things, object, objects, cargo, goods, merchandise, item, items, commodities, packages, products, baggage, shipment, shipments, stuff, material
Disease	disease, diseases, virus, viruses, infection, infections, illness, illnesses
Flood/Tide	flood, flooding, floods, ocean, oceans, river, rivers, stream, tide, tides, water, waters, wave, waves
Machines	machine, machines, machinery, equipment, apparatus, appliances, hardware, engine, engines, tool, tools, device, devices
Vermin/Pests	rat, rats, worm, worms, bug, bugs, parasite, parasites, insect, insects, pest, flea, rodents
Random	adoption, aerial, agricultural, amtrak, announcements, antenna, brave, cadet, captures, carroll, champaign, charley, ecosystem, excuses, exit, french, freshman, goal, headache, inter, knock, liberty, lifeboat, london, manifest, mrs, multimedia, narcotics, nitrate, orr, ow, parliamentary, plantation, proof, protect, provider, ready, reese, revolutionaries, ribbons, san, sanders, satisfaction, scope, series, sucker, superstructure, whig, whiskey

In addition, if we do the same comparison between groups (e.g., Republicans vs. Democrats) as we did for the dehumanizing metaphors, but using the *Random* category, we find that all comparisons are either not significant or show the opposite sign of the differences observed between parties and groups. Thus, we are confident that the observed differences are real, and report them without correction.

Examples. To provide intuition about the types of mentions found using this method, examples of sentences which cue the *Animal* metaphor are given in Table S12. Note that some examples also illustrate the presence of OCR errors in older data (see Methods and Materials in main paper).

Validation. To further validate that this method is picking up on types of language that humans would also see as indicating the presence of particular metaphorical categories, we collect annotations on a small number of examples for the *Animal* category. Using the full set of relevant mentions in the context, we sort them by combined log probability from the model for all animal terms, and draw a stratified set of 84 examples from across the distribution. We oversample the upper part of the distribution, as most examples have relatively low probabilities assigned to these words.

As a comparison set, we also identify all mentions of the target words in the *Animal* category in speeches about immigration, and embed all of these in the same way, again computing the probability assigned by the model to words in the category. Using the same stratification, we draw a sample of 42 words from these examples.

Four authors from this paper annotated these examples, with each example being annotated by two annotators, assigning an equal number to all possible pairings. Annotators were only shown the context and the [MASK] token, not the word or span that had been masked. The task was to identify all contexts where an animal term would be a plausible replacement for the [MASK] token, and for which there was some contextual cue that made it a plausible replacement. Overall agreement among annotators was 0.59 using Krippendorff's alpha.

For both the immigrant mentions, and the embeddings of literal animal terms, there is a strong correlation between average human rating (1 = yes, animal is a plausible replacement; 0 = no) and log probability from the model ($r = 0.73$ and 0.63, respectively, using Pearson correlation). Figure S30 shows a boxplot of log probabilities, plotted in terms of average human rating, for both immigrant mentions and animal terms. With the exception of a few outliers, those examples identified by humans as cueing the animal metaphor also have high probability from the model, and vice versa.

Looking at the list of examples given in Table S12, we note that some of the animal examples seem to be picking up on mentions of farms and ranches. In order to ensure that our results are not confounded by differences in how the two parties talk about agriculture in relation to immigration, we investigate these mentions in more detail.

To better understand what terms are driving our measurements of mentions of immigrants as suggestive of an *Animal* metaphor, we take all of the mentions of immigrants from the past two decades, and train a basic bag-of-words logistic regression model to differentiate between those that are found to be relatively highly indicative of this metaphor (the top quartile) and the rest. The resulting model shows that some of the most heavily weighted words are indeed related to agriculture, with the most heavily weighted among the agricultural terms being "agricultural", "dairy", "agriculture", and "farm". We then check the frequency of these terms within the contexts of immigration mentions from this time period among those speeches by

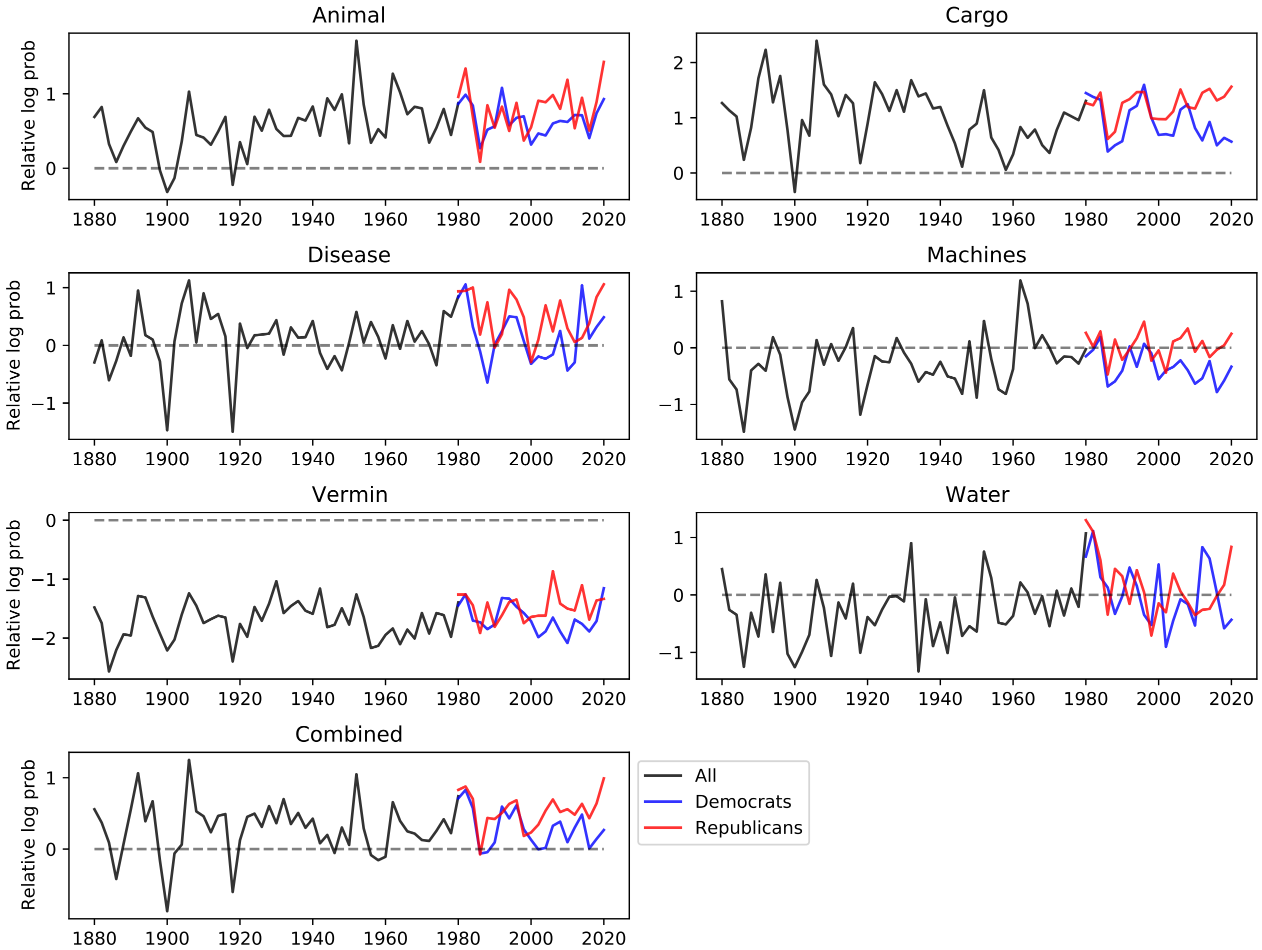

Fig. S29. Average relative log probabilities for each dehumanizing metaphor (as well as overall) for mentions of immigrants over time. Black lines show overall logged average probability prior to the period of polarization; red and blue lines show the averages for Republicans and Democrats, respectively. All lines represent the log of the average probability per word in the metaphorical category relative to the average probability per word for a set of random terms. For most metaphors, probabilities are significantly higher for Republicans (relative to Democrats) in the past two decades, but there is no significant increase or decrease in the overall probability of dehumanizing metaphors over time.

Table S12. The 12 contexts (and corresponding mention terms) most strongly suggestive of the *Animal* metaphor. Cumulative probability is the sum of probabilities assigned by the model to all words in the *Animal* category (see Table S11).

Cumulative probability	Year	Masked term	Context
0.97	1906	immigrants	in the breeding of our live stock of every description . and it would be just as unreasonable to claim that we will not lower american standards by admitting to our country [MASK] that are of lower standards than ours as it is to assert that the breeding of the thoroughbred kentucky horses will not be injured by breeding them with texas mustangs .
0.90	1961	migrants	still another instance of inadequate reporting is brought to my attention in the murrowfriendly production which referred to the regulation of transportation of produce and cattle . and then goes on to say thatonly six states have laws providing for the safe transportation of [MASK] within their borders .
0.87	1939	aliens	the destruction of these homes by a ruthless government . cruel separation of families . and the herding of these [MASK] in stockades is pictured .
0.82	1963	Mexican nationals	it was enacted at that time in order to provide effective control procedures for the movement of [MASK] into the farmlands in the united states .
0.80	1947	Cuban men	it happened to b - general weyler . who was herding [MASK] . women . and children in concentrados .
0.71	1952	wetbacks	the time required to prepare evidence . travel possibly hundreds of miles to a district immigration office to secure a warrant and return to make a search . would give ample time for the offending farm or farms to move their [MASK] out or lend them to a neighboring farm long before the enforcement officer could hope to put the warrant into the limited effect possible .
0.70	1882	Jews	mr . speaker . it was left for that broadminded ___ who wrote so splendidly the protestant history of england to say . when the jewish disability act was under discussion in the english par] iament . that for ages it had been the custom to call these men cursed [MASK] . dogs of ___ . but that the nation which could boast of isaiah among its poets and the maccabees aiong its generals could not be derided by any englishspeaking people .
0.70	2010	illegals	rancher john ladd counted some 350 [MASK] on his san jose ranch over a period of 18 days before this newspaper interview .
0.70	1952	aliens	between these two large farming operations . thousands of [MASK] have been taken over the last year from this area alone .
0.68	2019	migrants	for months . communities in texas have requested help in feeding . transporting . and sheltering these [MASK] .
0.66	1996	aliens	it establishes a positive framework to prevent illegal [MASK] from feeding at the public trough .
0.64	2002	illegals	border rancher george morgan encounters thousands of [MASK] crossing his ranch on a wellused trail .

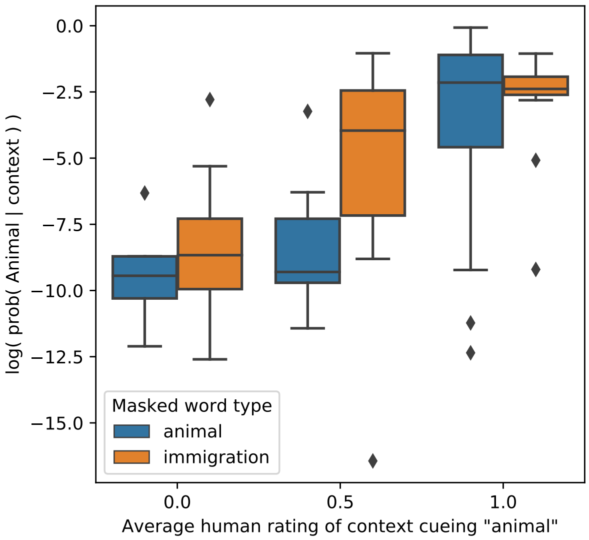

Fig. S30. Boxplot of cumulative log probabilities for terms in the *Animal* category (y-axis) divided into three categories based on human annotations (average of two binary ratings, where 1 = animal is a plausible replacement). Colors further divide those into examples where the masked token was originally a mention of immigrants (orange/right) vs those that were originally an animal token (blue/left). As expected, the model predicts that animal terms are more likely as replacements for examples where human annotators also think that an animal term is appropriate, for both types of examples.

Democrats and Republicans. For the sake of completeness, we consider the four terms mentioned above, along with singular and plural forms of "farm", "farmer", "ranch", and "ranchers". The mention frequencies by party are shown in Figure S31.

As can be seen, there is indeed a difference between the parties in terms of how frequently they refer to some of these terms in the context of immigration. However, the differences are somewhat symmetric, with Democrats referring more to farms and farmers, and Republicans referring more to ranches and ranchers. Moreover, among the most highly weighted among these terms, two are more frequently used by Democrats ("farm" and "dairy"), and two are more frequently used by Republicans ("agriculture" and "agricultural"). As such, we do not believe that the difference between the parties in terms of how much their mentions of immigrants cue the "animal" metaphor is driven primarily by a difference in their respective references to certain agricultural terms.

To be doubly sure, we re-run the metaphorical analysis after masking out all of the agriculture-related words listed in Figure S31 (i.e., replacing those tokens with "[MASK]". Although the numbers change slightly, this does change any of our conclusions. In particular, rounded to the nearest decimal, the resulting probability ratios (for the combined dehumanization metric) remain the same: 1.4 for Chinese:European in the early time period, 1.9 for Mexican:European in the past two decades, and 1.6 for Republicans:Democrats in the past two decades, with no significant difference between the parties in the earlier time period.

Finally, to verify that the observed differences in the use of dehumanizing metaphors are not excessively influenced by the fact that BERT was trained on modern data, we repeat the analysis using HistBERT—a version of BERT that has been fine-tuned to historical data, covering the entire 20th Century (13). Although this produces slightly different values, the results are essentially unchanged. The corresponding ratios when using HistBERT are 1.3 for Chinese:European ($p = 0.0019$), 2.2 for Mexican:European ($p < 0.001$), and 1.6 for modern Republican:Democrat ($p < 0.001$), with no significant difference between parties in the earlier time period.

Temporal Analysis of Frames

Although our use of immigration frames in the main paper is restricted to testing differences between parties and groups, they also allow us to study broader changes over time in how immigration is discussed. To do so, we plot the combined frequency of terms associated with each frame (with the appropriate part of speech tags), both in speeches about immigration, and in all non-procedural speeches (Figure S32).

Although the comparison is imperfect (because non-procedural speeches that are not about immigration represent a variety of different types of speech, not just those about comparable issues), we use the relative frequency, measured using pointwise mutual information (PMI), to measure how salient each frame was to the issue of immigration over time (Figure S33). To make the PMI scores comparable across sessions of Congress (which differ in the total number of tokens), we normalize these scores by dividing by the PMI score for the term "immigration", which almost never occurs except in speeches that have been classified as being about immigration (hence the use of "Scaled PMI" in these figures). We also use PMI to show the divergence between the parties on framing over time (Figure S34) and the overall trends for each party (Figure S35). Figures S33 and S34 again use the technique of leaving out each word in turn, and keeping the minimum and maximum values, in this case plotted as bands around the main line.

Over the past 150 years, in speeches about immigration, we find significant increases in the raw frequency of terms associated

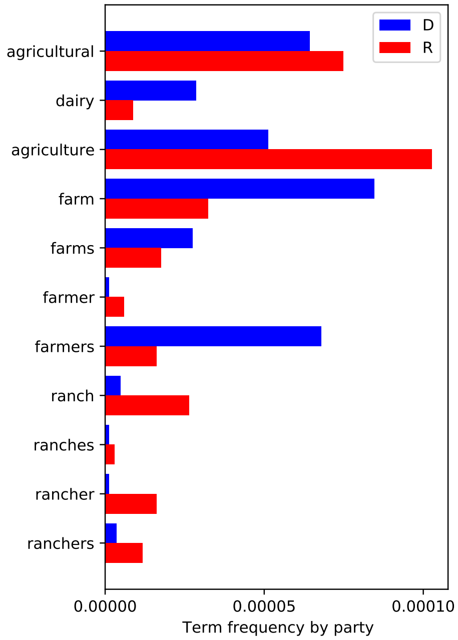

Fig. S31. Mention frequencies, by party, of agriculture-related words, in segments mentioning immigration from the past two decades. As can be seen, there is a difference between the parties, but this is not likely to explain the differences in our dehumanization metric, given that Republicans mention ranching more, whereas Democrats mention farming more.

with *Victims*, *Family*, *Legality*, *Quantity*, *Crime*, *Threats*, and *Contributions* (all significant at the $p < 0.001$ level; see Figure S32). For many of these frames, however, the frequency of the associated terms have also become more common in all non-procedural speeches. As a result, the only frames that show a significant increase in their association with immigration (as measured using PMI) are *Crime*, *Legality*, and *Victims*. Although each of these frames has always had some association with immigration, these associations have grown much stronger over time.

For *Legality*, this was driven partly by the rise in mentions of "illegal immigrants", but also relates to the expansion of the immigration bureaucracy, including issues related to visas and naturalization. The term of "illegal" also contributes to the growing association with *Crime*, but this association also depends on the popularly expressed notion that immigrants are bringing "drugs", committing "crimes", and connected to "terrorism".

By contrast, the only frame for which there has been a significant decline in raw frequencies within immigration speeches is *Deficient*, which was once extremely common due to references to immigrants said to be "illiterate", "diseased", or "undesirable". However, several frames show a significant relative decline in their association with immigration (measured using PMI), including *Deficient*, *Culture*, *Threats*, *Family*, *Labor*, *Contributions*, and *Economics*, as the terms associated with these frames have become relatively more frequently used in non-immigration speeches.

As expected, nearly all frames show a positive association with immigration during this time, because of how they were constructed (beginning with terms which referred to immigrants more frequently than generic person mentions). The one exception to this is *Economics*. Although economic terms are common in speeches about immigration, they are yet more frequent in non-immigration speeches, and this effect is stronger now than in the past, hence the significant decline in this association. For the differences between parties, these results largely match those that are shown in Figure 3 in the main paper. As shown in Figure S34, however, we can see that all of these differences have grown stronger over time since the 1980s. Those frames which show significant relative increases among Republicans since 1980 are, *Deficient*, *Crime*, and *Flood/Tide*. Those which show significant relative increases among Democrats over the same time period are *Family* and *Contributions*.

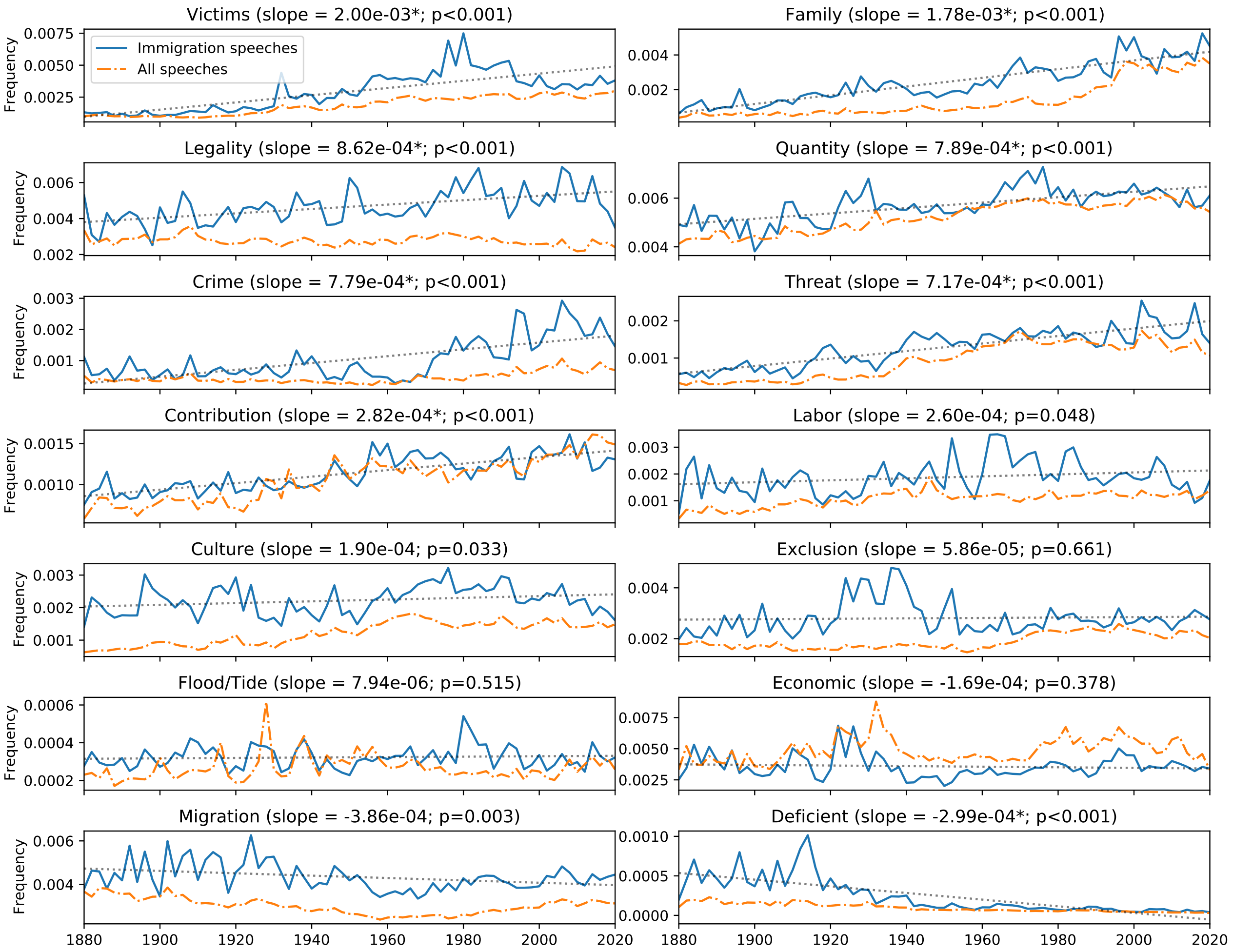

Fig. S32. Cumulative frequency of terms associated with each of our fourteen frames, both within speeches about immigration (blue solid line), and in all speeches (orange broken line). The dashed line and p-values in titles refer to the slope of a linear regression fit to the frequency within immigration speeches over time. As an illustrative example, note that the *Economics* frame is common within speeches about immigration, and has remained so over the entire 150 year time period, but is actually mentioned more frequently in all speeches, especially since the 1920s. By contrast, terms related to crime have surged in frequency in speeches about immigration, with a far less dramatic increase in overall frequency across all speeches. Note that each plot is scaled individually such that detailed variation is visible.

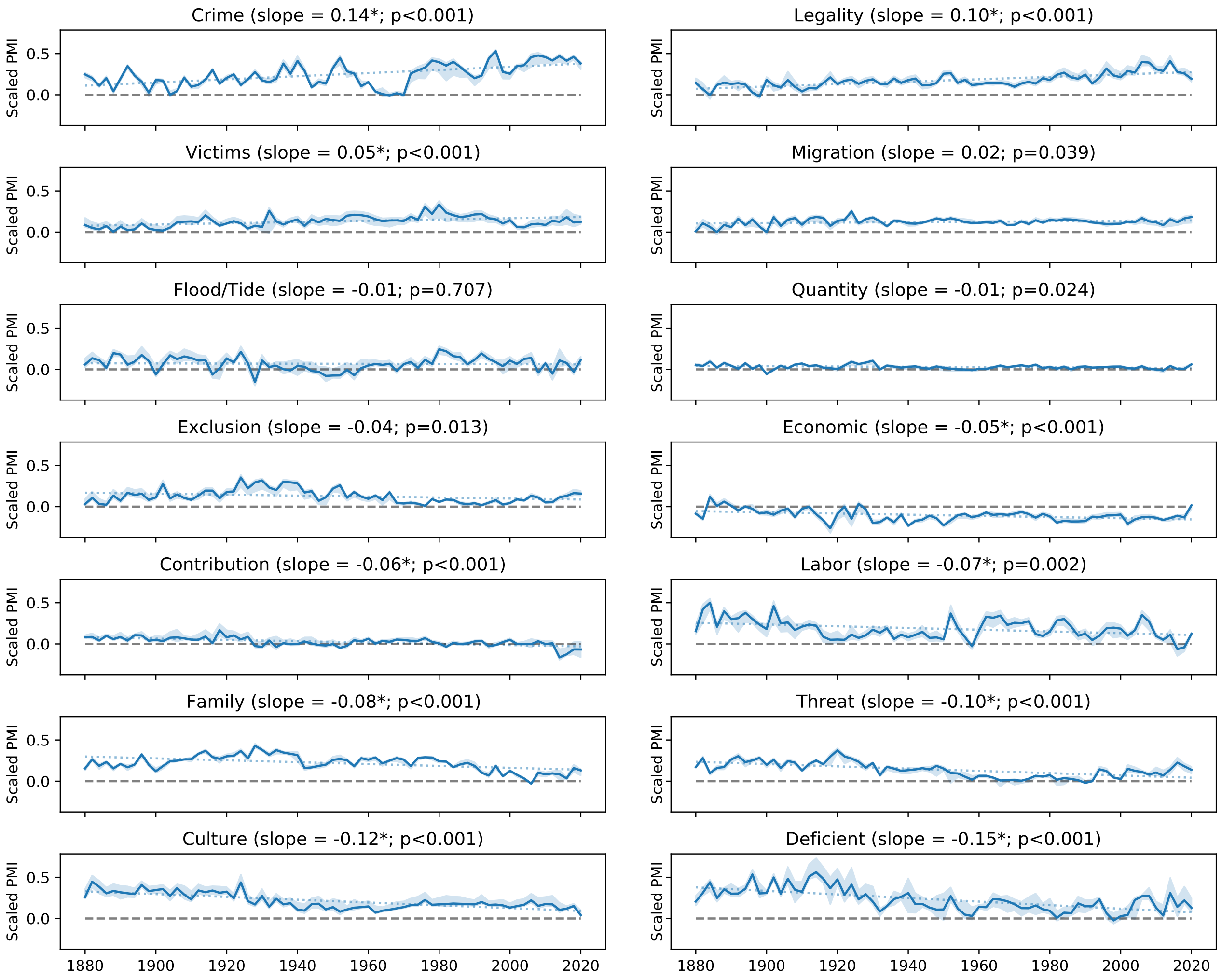

Fig. S33. Scaled PMI using all speeches, with slopes estimated over the entire time period from 1880-2020. Slopes are scaled such that a slope of 1.0 would be equivalent to moving from 0 to 1 in PMI over the entire time series. Asterisks indicate significant changes after applying a Bonferroni correction (with uncorrected p-values listed). Shaded bands indicate the area from minimum to maximum scaled PMI obtained from re-computing values when excluding out one term at a time from the terms associated with a given frame (showing that no single term has a massive effect on the slope, though significance would change in some cases if we took the maximum p-value). Note that all plots are scaled consistently and are comparable in absolute terms.

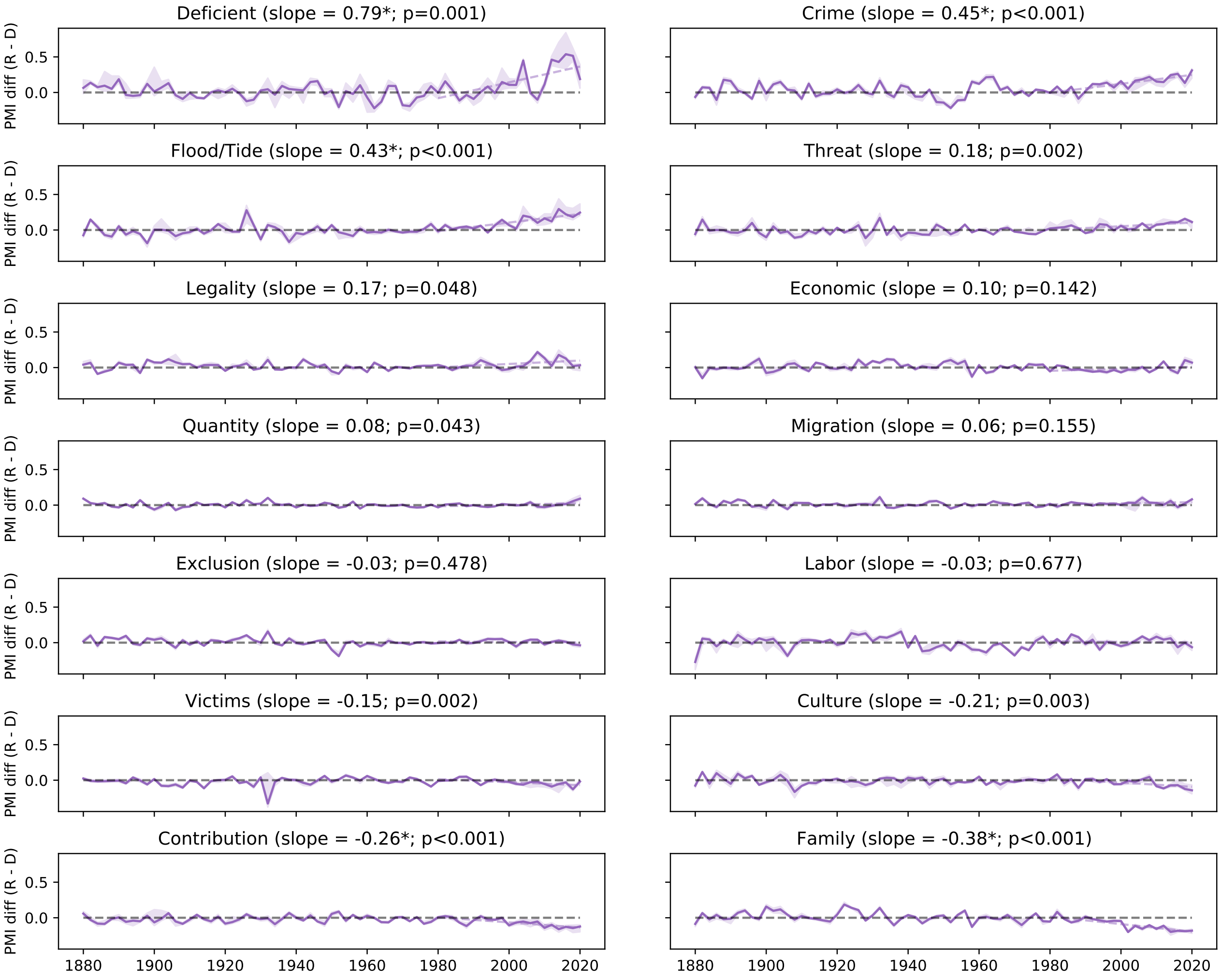

Fig. S34. Differences in scaled PMI (Republicans - Democrats), with slopes estimated over the period of partisanship from 1970-2020. Slopes are scaled such that a slope of 1.0 would be equivalent to moving from 0 to 1 in PMI over the entire time series. As above, asterisks show statistical significance after Bonferroni correction, and bands show the minimum and maximum scaled PMI when leaving out one word at a time.

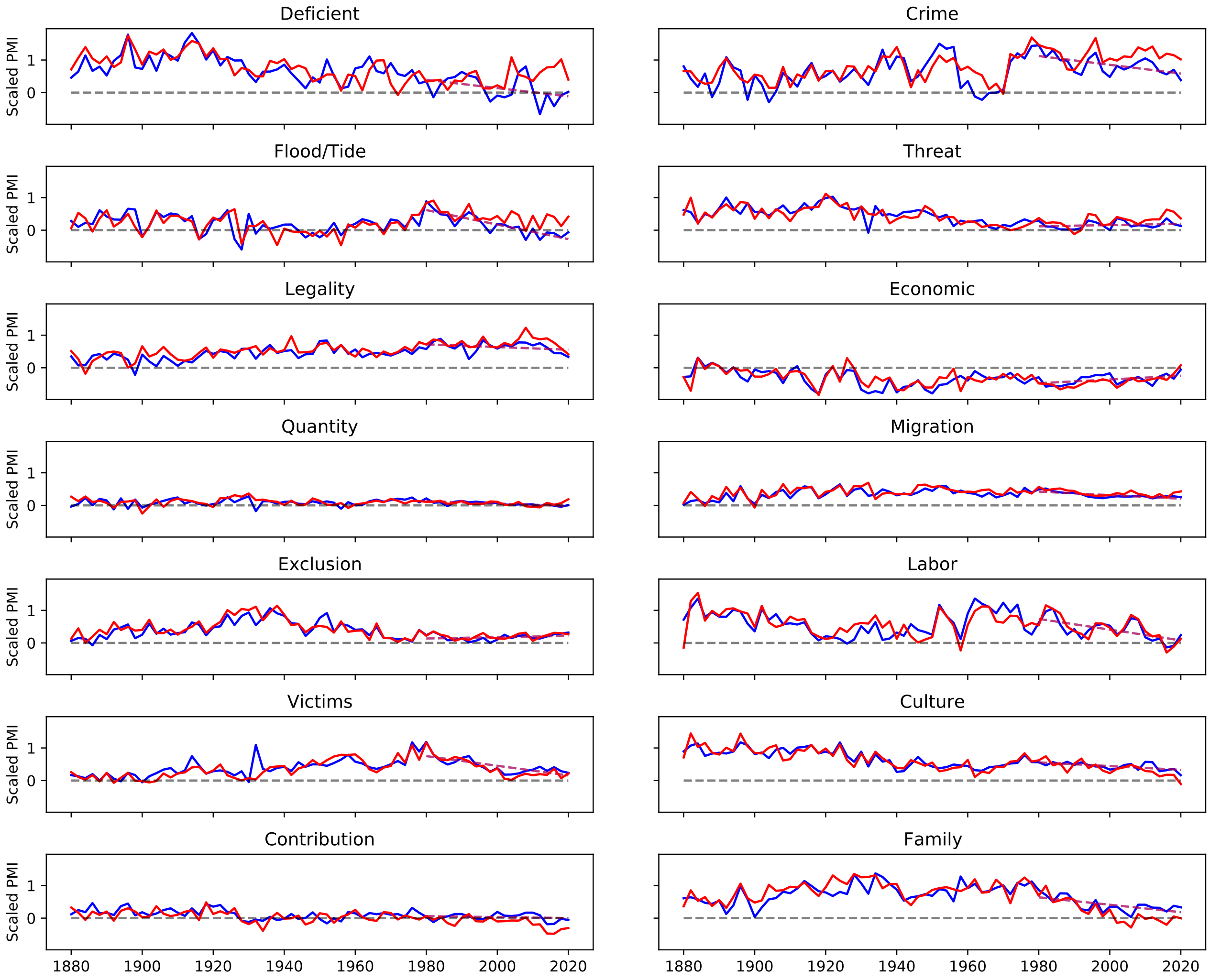

Fig. S35. Scaled PMI computed separately by party.

References

1. M Gentzkow, JM Shapiro, M Taddy, *Congressional Record for the 43rd-114th Congresses: Parsed Speeches and Phrase Counts.* (Stanford Libraries, Palo Alto, CA), (2018).
2. D Card, AE Boydstun, JH Gross, P Resnik, NA Smith, The media frames corpus: Annotations of frames across issues. in *Proceedings of ACL.* (2015).
3. Y Luo, D Card, D Jurafsky, Detecting stance in media on global warming. in *Findings of the Association for Computational Linguistics: EMNLP 2020.* (2020).
4. S Wang, C Manning, Baselines and bigrams: Simple, good sentiment and topic classification. in *Proceedings of the ACL.* pp. 90–94 (2012).
5. A Noorani, Retiring Miami Rep. Ros-Lehtinen's set an example for tackling immigration issues. *Miami Her.* (2018) Accessed 2022-05-07.
6. J Preston, The immigration crisis Jeff Sessions leaves behind. *The Marshall Proj.* (2018).
7. SD Team, Stan modeling language users guide and reference manual 2.27 (2019).
8. R Carroll, JB Lewis, J Lo, KT Poole, H Rosenthal, Measuring bias and uncertainty in DW-NOMINATE ideal point estimates via the parametric bootstrap. *Polit. Analysis* **17**, 261—275 (2009).
9. JB Lewis, et al., Voteview: Congressional roll-call votes database. *https://voteview.com* (2022).
10. DM Blei, AY Ng, MI Jordan, Latent dirichlet allocation. *J. Mach. Learn. Res.* **3**, 993–1022 (2003).
11. T Mikolov, I Sutskever, K Chen, GS Corrado, J Dean, Distributed representations of words and phrases and their compositionality. in *Advances in Neural Information Processing Systems*, eds. CJC Burges, L Bottou, M Welling, Z Ghahramani, KQ Weinberger. (Curran Associates, Inc.), Vol. 26, (2013).
12. J Devlin, MW Chang, K Lee, K Toutanova, BERT: Pre-training of deep bidirectional transformers for language understanding. in *Proceedings of NAACL.* (2019).
13. Wenjun Qiu, Y Xu, HistBERT: A pre-trained language model for diachronic lexical semantic analysis. *arXiv:2202.03612* (2022).
